# Comparison of anonymization techniques regarding statistical reproducibility

**DOI:** 10.1371/journal.pdig.0000735

**Published:** 2025-02-03

**Authors:** David Pau, Camille Bachot, Charles Monteil, Laetitia Vinet, Mathieu Boucher, Nadir Sella, Romain Jegou

**Affiliations:** 1 Medical Evidence and Data Science Unit, Roche, Boulogne-Billancourt, France; 2 Informatics Department, Roche, Boulogne-Billancourt, France; 3 Department of Statistics, Keyrus Life Science, Nantes, France; 4 Institut Roche, Roche, Boulogne-Billancourt, France; Fundación Progreso y Salud: Junta de Andalucia Consejeria de Salud y Familias Fundacion Progreso y Salud, SPAIN

## Abstract

**Background:**

Anonymization opens up innovative ways of using secondary data without the requirements of the GDPR, as anonymized data does not affect anymore the privacy of data subjects. Anonymization requires data alteration, and this project aims to compare the ability of such privacy protection methods to maintain reliability and utility of scientific data for secondary research purposes.

**Methods:**

The French data protection authority (CNIL) defines anonymization as a processing activity that consists of using methods to make impossible any identification of people by any means in an irreversible manner. To answer project’s objective, a series of analyses were performed on a cohort, and reproduced on four sets of anonymized data for comparison. Four assessment levels were used to evaluate impact of anonymization: level 1 referred to the replication of statistical outputs, level 2 referred to accuracy of statistical results, level 3 assessed data alteration (using Hellinger distances) and level 4 assessed privacy risks (using WP29 criteria).

**Results:**

87 items were produced on the raw cohort data and then reproduced on each of the four anonymized data. The overall level 1 replication score ranged from 67% to 100% depending on the anonymization solution. The most difficult analyses to replicate were regression models (sub-score ranging from 78% to 100%) and survival analysis (sub-score ranging from 0% to 100. The overall level 2 accuracy score ranged from 22% to 79% depending on the anonymization solution. For level 3, three methods had some variables with different probability distributions (Hellinger distance = 1). For level 4, all methods had reduced the privacy risk of singling out, with relative risk reductions ranging from 41% to 65%.

**Conclusion:**

None of the anonymization methods reproduced all outputs and results. A trade-off has to be find between context risk and the usefulness of data to answer the research question.

## Author summary

Anonymization is a processing of personal data to generate anonymous synthetic data. This project aimed to evaluate the capability of anonymization methods in maintaining reliability and utility of scientific data for research purposes, by comparing the impact of four different anonymization methods. The comparison of 4 anonymization methods is the novelty of this work and provides insight into the statistical and scientific value that remained. We found that no anonymized data reproduced accurately all results and these methods are not currently powerful enough for use in regulatory submissions. We also found that there is a lack of standardized privacy metrics to comprehensively evaluate and compare the effectiveness of anonymization techniques. As one size does not fit all, we strongly recommend the design of anonymization algorithms to be align with the data reuse objectives (e.g. open data, internal reuse or reuse by a unique external researcher).

## Introduction

The use of secondary data and data sharing are becoming key drivers for real-world evidence generation, allowing the healthcare ecosystem to gain more medical insights. Patient-level data collected in clinical trials are generally pseudonymized personal data under the General Data Protection Regulation (GDPR) [1], and therefore have legal and ethical obligations associated with it. Currently, for a secondary data exploitation of a pseudonymized clinical dataset, patients must be re-informed about the secondary data usage (with a new information notice, patient consent and/or patient portal if applicable), and obtain approval from local data protection in accordance with GDPR Article 6. This process is time-consuming and difficult for all parties involved.

Anonymization is defined by the French data privacy Commission Nationale de l’Informatique et des Libertés (CNIL) [2] as a processing activity that consists of using methods to make impossible any identification of people by any means in an irreversible manner. Anonymization is a method of reducing the privacy risks associated with patient-level clinical trial data, once anonymized data records are no longer considered personal data. As a result, the GDPR principles of data protection do not apply to anonymous information. Anonymization enables data to be re-used and, subject to certain safeguards, allows data to be shared while maintaining patient privacy. Furthermore, anonymization allows data to be retained beyond the legal limits. In 2014, the Article 29 Data Protection Working Party (WP29), which was replaced by the European data protection board when the GDPR came into force, published an opinion on anonymization techniques [3] to evaluate the effectiveness and limits of such techniques based on three criteria (singling out, linkability and inference), described in statistical methods.

As mentioned by Olatunji et al. in 2022 [4], numerous privacy models and techniques have been proposed in the literature to achieve anonymization, and a comprehensive review of anonymization models and techniques is provided. However, anonymization algorithms may lead to significant information loss which can prevent researchers from conducting relevant statistical analyses.

**The objective of this project** was to evaluate the capability of privacy protection methods in maintaining the reliability and utility of scientific data for research purposes, by comparing the impact of different anonymization methods on the statistical and scientific value of the data. We also seek to provide recommendations before considering using anonymization. The comparison of 4 anonymization methods is the novelty of this work.

## Project data

The aim of the initial study was to describe the epidemiology and the therapeutic management of 315 patients treated for early breast cancer among 57 sites in France. Data extraction was performed in September 2019. The raw data were based on the Standard Data Tabulation Model (SDTM) format [5], including information on demographic characteristics, diagnosis, surgery, pathological complete response, adjuvant treatments, patients follow-up and information on patients lost to follow-up.

## Material and anonymization methods

Many companies offer anonymization tools and services with promising value to ease data reuse (vendor). It should be noted that anonymization is not a process that can be applied homogeneously to every situation: depending on the use case, the level of data alteration can be different and amplified.

**Fig 1:**
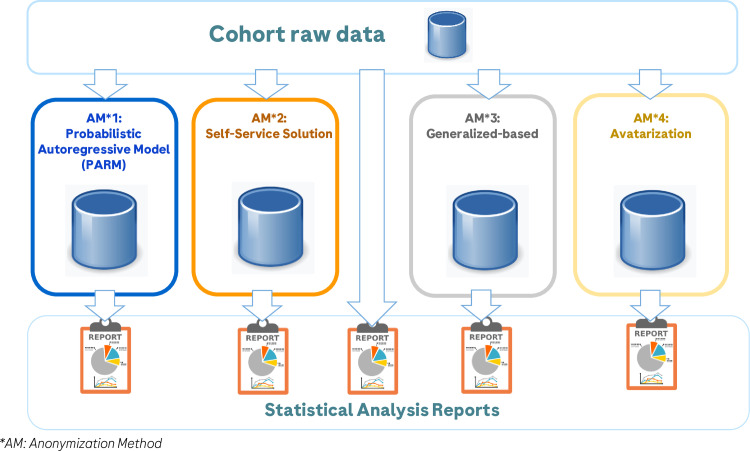
Data flow.

**To answer the project objective, four vendors using different Anonymization Methods (AM) or tools (** **Fig 1** **) were selected to determine the impact of their anonymization on the statistical and scientific value of the data while preserving data privacy.**

Each vendor chose the method they thought most appropriate to answer project objectives:

AM1: The first method was an open-source Python model [6] for generating synthetic data based on probabilistic autoregressive models (PARM).
AM2: The second method (self-service solution) was a tool developed from scratch for this project to provide anonymization features following the techniques recommended by the WP29 (3). The tool is intended to be used directly by the data owner, preferably with the support of a GDPR expert.
AM3: The third method (generalization-based) was a service provided by a data protection specialist company that integrated regulatory obligations, data protection rules, societal expectations and ethical issues into secondary data use projects.
AM4: The fourth approach was based on a patented algorithm called avatarization [7], which involves projecting individuals into a multi-dimensional space using a dimensionality reduction technique. Subsequently, the method comprises three steps for each individual: i) identifying the k-nearest neighbors in the projected space, ii) generating a new instance in this lower-dimensional space (avatar), and iii) projecting the avatar back into the original space while preserving the original encoding.

The second (self-service solution) and third (generalization-based) methods are more straightforward and easier to implement as they are based on simple privacy preserving techniques applied variable by variable (techniques that consist in applying functions such as aggregation, noise addition, permutation, hashing, top and bottom coding to remove, mask or provide less detail on personal data), while the other two are based on more complex algorithms that take into account high dimensional space. It is important to note that the study protocol and the data dictionary were shared with each vendor, providing them with the same level of prior information about the input data. **Each vendor was given the freedom to apply the degree of alteration they deemed appropriate for each variable**. In order to maximize the usability of the anonymized data and to perform the same statistical analyses as on the raw data, each vendor was required to maintain the same variable structure (name and format) as the raw data.

## Ethics statement

This research was based on data from a French real-world retrospective study collected from patient’s electronic medical records. The cohort study was approved by the Institutional Review Board/Independent Ethics Committee on May 03, 2018, prior study set-up. All the patients received written information before any trial-related activities were carried out. The patients were not reinformed about the four anonymization techniques applied to the data, as the same statistical analysis was conducted on the anonymized data as was originally planned in the initial research.

## Statistical methods and level of evaluation of the anonymization data

The analyses were first generated on the raw data, considered as the reference, and then reproduced on each of the four anonymized data. Eleven variables (see S1 File) were derived in order to facilitate further statistical output and modeling.

The following analysis were then carried out on raw and anonymized dataset:

Descriptive statistics of patient and disease characteristics (mean, standard deviation, median, quartiles for continuous variables and proportion for categorical variables).

Descriptive statistics stratified by adjuvant treatment.

Logistic regression model [8] to identify predictive factors for pathological complete response (pCR) among baseline characteristics by estimating the odds ratio (OR) with its 95% confidence interval (95% CI) and associated p-value.

Survival analysis to identify predictive factors for progression-free survival (PFS) by estimating the hazard ratio (HR) with its 95% CI and associated p-value using the cox model. This analysis included a Kaplan-Meier curve and a Cox model.

Correlation matrix to assess associations between nine variables (p-value from chi-squared or fisher’s exact test between categorical variables and global effect p-value from the analysis of variance (ANOVA) between continuous and categorical variables).

Five statistical reports (one for raw data and four for each AM) included a total of 87 individual items each, which were used as a basis to evaluate the impact of anonymization on the statistical and scientific value of the data.

**Four levels of evaluation were used to assess the impact of anonymization** on the variables and statistical analyses initially carried out on the raw data:

**Level 1 (replication score) refers to the number of variables or statistical analyses that could be replicated** by each anonymized data. The level 1 total replication score ranges from 0 item (no statistical item could be replicated) to 87 items (all statistical items could be replicated), standardized on a scale of 0–100%, which is the sum of the six level 1 sub-scores, categorized by type of analysis: data transformation (range: 0–11), descriptive statistics (0–38), treatment-related descriptive statistics (0–9), survival analysis (0–11), logistic analysis (range: 0–9), and correlation matrix (0–9). A binary outcome was derived for each item, indicating whether it was replicable or not (yes versus no).

**Level 2 (accuracy score) refers to the number of statistical analyses that provides similar results** compared to the raw analysis, as outlined below by type of analysis:

Descriptive statistics for 47 variables (including nine treatment-related variables): Difference in mean for continuous variables and difference in proportion for categorical variables, with the associated 95% CI estimated using a bootstrap method [9,10]. The rules for determining similarity were as follows: The absolute difference between the raw result and each anonymized result was less than 5% for proportion and mean, or the 95% CI of the difference from the raw result included zero.

For survival analysis, Kaplan-Meier curves were examined to determine whether the global effect p-value was still significant at 15% for the nine univariate Cox models and the multivariate Cox model.

For logistic analysis, we assessed whether the global effect p-value was still significant at 15% for the eight univariate models and the multivariate model.

For the correlation matrix analysis, we assessed whether the chi-square/fisher’s exact test or anova type 3 test p-values for each variable were still significant at 5%.

The level 2 total score ranges from 0 (no statistical results were replicated and similar) to 76 (all statistical results were replicated and similar), standardized on a scale of 0–100%, and is the sum of the five level 2 sub-scores, categorized by type of analysis: descriptive statistics (range: 0–38), treatment-related descriptive statistics (0–9), survival analysis (0–11), logistic analysis (range: 0–9), and correlation matrix (0–9). For each item a binary outcome was derived, indicating whether the item was replicated and similar or not (yes versus no).

**Level 3 (alteration level) assessed the degree of data alteration and the relationships** between variables in each anonymization solution as described below.

The **degree of alteration** was measured by:

The **Hellinger distance** [11] was used to measure the similarity between the probability distribution of the raw data and each anonymized data. The score ranges from 0 (no difference) to 1 (maximum distance). Eighteen source variables representing patient and disease characteristics were included in this analysis.

**Data availability** was assessed by calculating the difference in the number of patients with no missing data between the raw data and each anonymized data for the 48 descriptive statistical variables (including the 47 descriptive variables and the Kaplan-Meier censoring variable). A difference of less than 5% was considered similar.

The **relationship between variables** was evaluated by:

The **correlation coefficient** was calculated using spearman’s method [12], and correlograms were generated to visualize the relationship between each pair of a selected set of variables treated as ordinal rank-based measures. Thirteen source variables representing patient and disease characteristics were used, resulting in a total of 78 correlation coefficients. A difference of less than 0.1 points between the raw spearman coefficient and each anonymized method was considered to be similar.

A **multiple correspondence analysis (MCA)** [13,14] was conducted using 12 source variables representing disease characteristics to identify and represent underlying structural differences between raw and anonymized data.

**Level 4** (**WP29 compliance**) **evaluated the privacy according to WP29 criteria**, as follow:

**Singling out**, as it should not be possible to isolate an individual in the data, we identified the number of times that a patient was available in a modality of qualitative variables and estimated a relative risk reduction of singling out.

**Linkability**, as it should not be possible to link different data sets concerning the same individual, we estimated the risk of re-identification of a patient participating in the cohort using a quantitative approach [15], with external national epidemiological data sources. We estimated acquaintance’s probabilities, which is the risk of re-identification depending on the level of knowledge (knowledge ranging from “a patient having breast cancer in France” to “a patient having an early breast cancer in France who underwent surgery”).

**Inference**, as it should not be possible to deduce new information about an individual with a high degree of certainty, we used a ℓ-diversity privacy approach [16]. As the main idea behind ℓ-diversity is that the values of sensitive attributes are well represented in each modality, we estimated a k-anonymity probability threshold, with k depending on the number of modalities of each variable. We determined the number of times that a modality of qualitative variables with at least five modalities was above the threshold. We estimated a relative risk reduction of inference.

All analyses were conducted using R4.1.3 software.

## Results

315 patients were included in the cohort and analyzed in this project, the raw mean age was 52.2 years (SD:12.6), Scarff-Bloom-Richardson (SBR) grade II (45.9%), SBR grade III (49.8%), invasive ductal carcinoma (92.8%) and 41.9% of patients had a pathological complete response (pCR) at surgery. The cohort descriptions before anonymization (raw) and after each anonymization method are shown in Table 1 and the results per level are summarized in Table 2.

**Table 1:** Summary of baseline characteristics.

Characteristic	Raw (reference) N = 315	PARM N = 315	Self-service N = 315	Generalization-based N = 315	Avatarization N = 315
Age at adjuvant treatment (years)					
Mean (SD)	52.18 (12.64)	NC	52.56 (12.63)	NC	52.23 (11.82)
Median (Q1;Q3)	52.0 (43.0; 60.5)	NC	52.0 (43.0; 61.0)	NC	52.0 (43.0; 60.5)
Missing	12	315	12	315	12
BMI (kg/m^2^) n/N (%)					
<25	156/311 (50.2%)	98/315 (31.1%)	185/311 (59.5%)	148/311 (47.6%)	146/313 (46.6%)
[25–30]	82/311 (26.4%)	114/315 (36.2%)	32/311 (10.3%)	92/311 (29.6%)	117/313 (37.4%)
≥30	73/311 (23.5%)	103/315 (32.7%)	94/311 (30.2%)	71/311 (22.8%)	50/313 (16.0%)
Missing	4	0	4	4	2
BMI (kg/m^2^)					
Mean (SD)	25.85 (5.97)	28.44 (7.40)	5x10^7^ (9x10^8^)	25.84 (5.92)	25.58 (4.58)
Median (Q1;Q3)	24.0 (21.0; 29.0)	27.0 (23.5; 31.5)	21.0 (1.0; 34.5)	25.0 (21.0; 29.0)	25.0 (22.0; 28.0)
Missing	4	0	4	4	2
Histology at diagnosis, n/N (%)					
Invasive ductal carcinoma	284/306 (92.8%)	275/306 (89.9%)	284/306 (92.8%)	284/306 (92.8%)	291/306 (95.1%)
Invasive lobular carcinoma	10/306 (3.3%)	9/306 (2.9%)	10/306 (3.3%)	10/306 (3.3%)	9/306 (2.9%)
Mixed carcinoma	1/306 (0.3%)	4/306 (1.3%)	1/306 (0.3%)	1/306 (0.3%)	0
Other	9/306 (2.9%)	12/306 (3.9%)	9/306 (2.9%)	9/306 (2.9%)	6/306 (2.0%)
Unknown	2/306 (0.7%)	6/306 (2.0%)	2/306 (0.7%)	2/306 (0.7%)	0
Missing	9	9	9	9	9
pCR status at surgery, n (%)					
Yes	132 (41.9%)	108 (34.3%)	132 (41.9%)	132 (41.9%)	127 (40.3%)
No	183 (58.1%)	207 (65.7%)	183 (58.1%)	183 (58.1%)	188 (59.7%)
Adjuvant treatment, n (%)*					
Trastuzumab (Herceptin)	305 (100%)	25 (10.3%)	305 (100%)	305 (100%)	304 (99.7%)
Tamoxifene	81 (26.6%)	34 (14.0%)	81 (26.6%)	81 (26.6%)	81 (26.6%)
Letrozole	40 (13.1%)	28 (11.5%)	40 (13.1%)	40 (13.1%)	40 (13.1%)
Anastrozole	21 (6.9%)	34 (14.0%)	21 (6.9%)	21 (6.9%)	20 (6.6%)
Exemestane	5 (1.6%)	26 (10.7%)	5 (1.6%)	5 (1.6%)	5 (1.6%)
Other hormonotherapy 1	4 (1.3%)	25 (10.3%)	4 (1.3%)	3 (1.0%)	3 (1.0%)
Other	3 (1.0%)	38 (15.6%)	3 (1.0%)	6 (2.0%)	2 (0.7%)
Carboplatine	1 (0.3%)	35 (14.4%)	1 (0.3%)	0	0
Docetaxel	1 (0.3%)	41 (16.9%)	1 (0.3%)	0	0
Epirubicine	1 (0.3%)	26 (10.7%)	1 (0.3%)	0	0
Paclitaxel	1 (0.3%)	33 (13.6%)	1 (0.3%)	0	0
Other hormonotherapy 2	0	17 (7.0%)	0	0	0
5-FU	0	22 (9.1%)	0	0	0
Navelbine	0	28 (11.5%)	0	0	0
Cyclophosphamide	0	27 (11.1%)	0	0	0
Doxorubicine	0	25 (10.3%)	0	0	0

*One patient can have reported several adjuvant treatment types.

N = total number of individual patients in the data.

**Table 2:** Summary of the evaluation scores by level for each anonymization method.

Level	PARM	Self-service	Generalization-based	Avatarization
**Level 1 sub-score (by to type of analysis)**				
Data transformation (n = 11 variables derived)	45.5%	100%	81.8%	100%
Descriptive statistics (n = 38 variables summarized)	89.5%	100%	92.1%	100%
Descriptive statistics – treatment-related (n = 9 variables summarized)	66.7%	44.4%	88.9%	100%
Survival analysis (n = 11 models compiled)	0%	100%	81.8%	100%
Logistic regression (n = 9 models compiled)	77.8%	100%	77.8%	100%
Correlation matrix (n = 9 variables assessed)	77.8%	100%	77.8%	100%
**Level 1 total replication score** (n = 87 items)	67.8%	94.3%	86.2%	100%
**Level 1 descriptive statistics sub-score by variable format**				
Continuous variables (n = 16)	68.7%	81.2%	87.5%	100%
Categorical variables (n = 31)	93.5%	93.5%	93.5%	100%
**Level 1 sub-score according to descriptive/modeling**				
Descriptive statistics (n = 48)	83.3%	89.6%	91.7%	100%
Statistical modeling (n = 28)	50.0%	100%	78.6%	100%
**Level 2 sub-score (by type of analysis)**				
Descriptive statistics (n = 38 variables summarized)	31.6%	79.0%	86.8%	68.4%
Descriptive statistics – treatment-related (n = 9 variables summarized)	0%	33.3%	88.9%	88.9%
Survival analysis (n = 11 models compiled)	0%	100%	72.7%	63.6%
Logistic regression (n = 9 models compiled)	55.6%	100%	77.8%	77.8%
Correlation (n = 9 variables assessed)	0%	66.7%	44.4%	0%
**Level 2 total score** (n = 76 statistical items)	22.4%	77.6%	79.0%	63.2%
**Level 2 descriptive statistics sub-score by variable format**				
Continuous variables (n = 16 variables summarized)	0%	31.3%	87.5%	81.3%
Categorical variables (n = 31 variables summarized)	38.7%	90.3%	87.1%	67.7%
**Level 2 sub-score according to descriptive/modeling**				
Descriptive statistics (n = 48)	25.0%	70.8%	87.5%	70.8%
Statistical modeling (n = 28)	17.9%	89.3%	64.3%	50.0%
**Level 3**				
Probability distribution				
Hellinger distance = 0	0	11/18 (61.2%)	8/18 (44.5%)	0
Hellinger distance >0 and <0.1	12/18 (66.7%)	1/18 (5.5%)	8/18 (44.5%)	13/18 (72.2%)
Hellinger distance >0.1 and <1	5/18 (27.8%)	2/18 (11.1%)	1/18 (5.5%)	5/18 (27.8%)
Hellinger distance = 1	1/18 (5.5%)	4/18 (22.2%)	1/18 (5.5%)	0
Same number of patients in output	14/48 (29.2%)	43/48 (89.6%)	40/48 (83.3%)	42/48 (87.5%)
Correlation matrix	38/66* (57.6%)	67/78 (85.9%)	66/66* (100%)	65/78 (83.3%)
Multiple correspondence analysis				
Euclidean distances from Raw on the first 2 dimensions	0.25	0.03	0.17	0.08
Ellipse area	1.74	4.81	4.81	4.61
**Level 4**				
Singling out (raw = 15.5%)	5.5%	9.1%	7.3%	6.4%
Singling out relative risk reduction	64.5%	41.2%	52.9%	58.8%
Linkability		No linkage of individual anonymised patient data to individual raw patient data possible.		
		Linkability globally assessed for a patient participating in the cohort.		
Inference (raw = 51.3%)	48.7%	35.9%	38.5%	30.8%
Inference relative risk reduction	5.0%	30.0%	25.0%	40.0%

*Age at adjuvant treatment initiation not available.

Level 1 score = number of variables or statistical analyses that could be replicated using each privacy-preserving anonymized data, as produced for the raw analysis, without evaluating the accuracy of the reproduced variables or analyses. The level 1 total replication score ranges from 0 item (no statistical item could be reproduced) to 87 items (all statistical items could be reproduced), which is standardized on a scale of 0–100%. The level 1 total score is the sum of the six level 1 sub-scores, categorized by type of analysis: data transformation (range: 0–11), descriptive statistics (0–38), descriptive statistics treatment-related (0–9), survival analysis (0–11), logistic analysis (range: 0–9), and correlation matrix (0–9).

Level 2 score = number of statistical analyses that could be replicated using each privacy-preserving anonymized data, with similar results to the raw analysis. The level 2 total score ranges from 0 (no statistical results were replicated and similar) to 76 (all statistical results were replicated and similar), standardized on a scale of 0–100%. The level 2 total score is the sum of the five level 2 sub-scores, categorized by type of analysis: descriptive statistics (range: 0–38), descriptive statistics treatment-related (0–9), survival analysis (0–11), logistic analysis (range: 0–9), and correlation matrix (0–9).

Level 3 assessed the degree of alteration and the relationships between variables in each anonymization data versus the raw data.

Level 4 evaluated the privacy according to WP29 criteria (singling out, linkability and inference).

**Fig 2:**
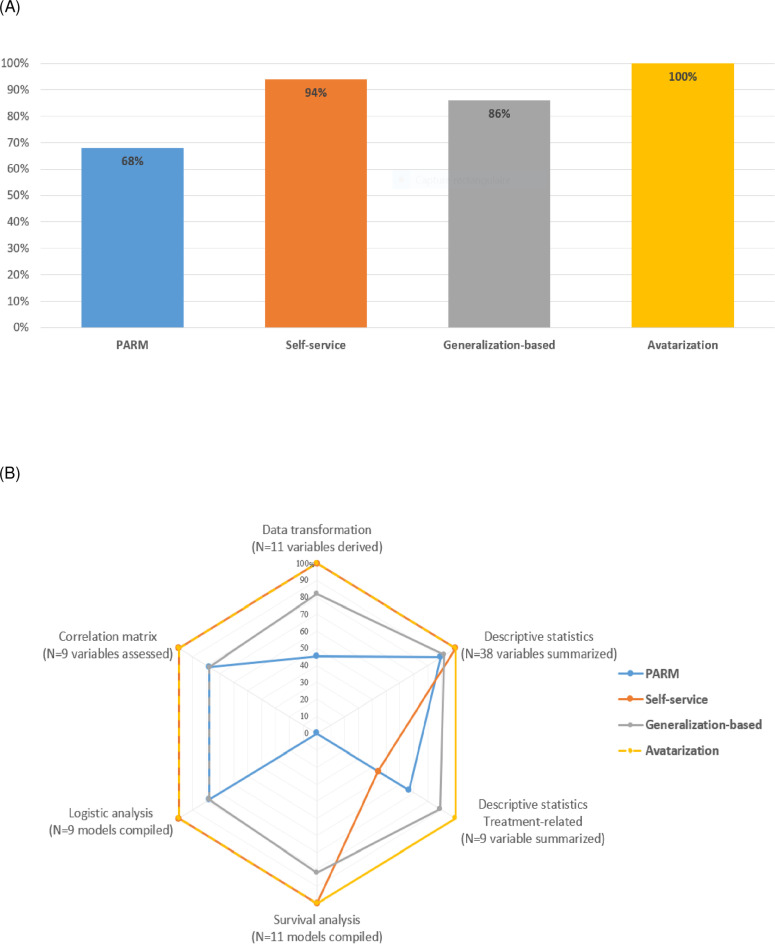
Level 1 replication score by anonymization method. (A) Level 1 total score = Number of variables or statistical analyses that could be replicated utilizing each privacy-preserving anonymized data, as produced for the raw analysis without evaluating the accuracy of the reproduced variables or analyses. Level 1 total replication score ranges from 0 item (no statistical item could be reproduced) to 87 items (all statistical item could be reproduced), which is standardized on a scale of 0–100%. (B) Level 1 sub-score are categorized by type of analysis: data transformation (range 0–11), descriptive statistics (0–38), descriptive statistics treatment-related (0–9), survival analysis (0–11), logistic regression analysis (0–9) and correlation matrix (0–9).

**The level 1 total replication score**, which assesses the ability to replicate derived variables or statistical analyses in anonymised data, **ranged from 67.8% to 100% depending on the anonymization method (Fig 2A**):

Avatarization was the only method that maintained the ability to replicate all derivations and statistical analyses (level 1 total replication score is 100%).

For the self-service method, 37% of variables were not modified (decisions made variable by variable by a dedicated multidisciplinary team) in order to preserve statistical value, resulting in 94.2% of items being replicated.

The generalization-based approach achieved a score of 86.2%, despite the fact that the date of birth was removed, which degraded the results of the data transformation and associated statistical analyses.

The PARM approach had the lowest score with 67.8% of items replicated, probably due to the high level of data curation required before and after using the algorithm.

**Level 1 replication sub-scores by type of analysis, are shown in Fig 2B**. Reproducing regression models (with level 1 sub-scores ranging from 78% to 100%) and survival analysis (with level 1 sub-scores ranging from 0% to 100%) using anonymized data proved to be the most challenging for some anonymization methods. This is due to the need for numerous data transformations, including derived variables such as age and survival time, which were not consistently available. Specifically, the level 1 data transformation sub-scores were 81.8% and 45.5% for the generalization-based and PARM approaches, respectively.

**Fig 3:**
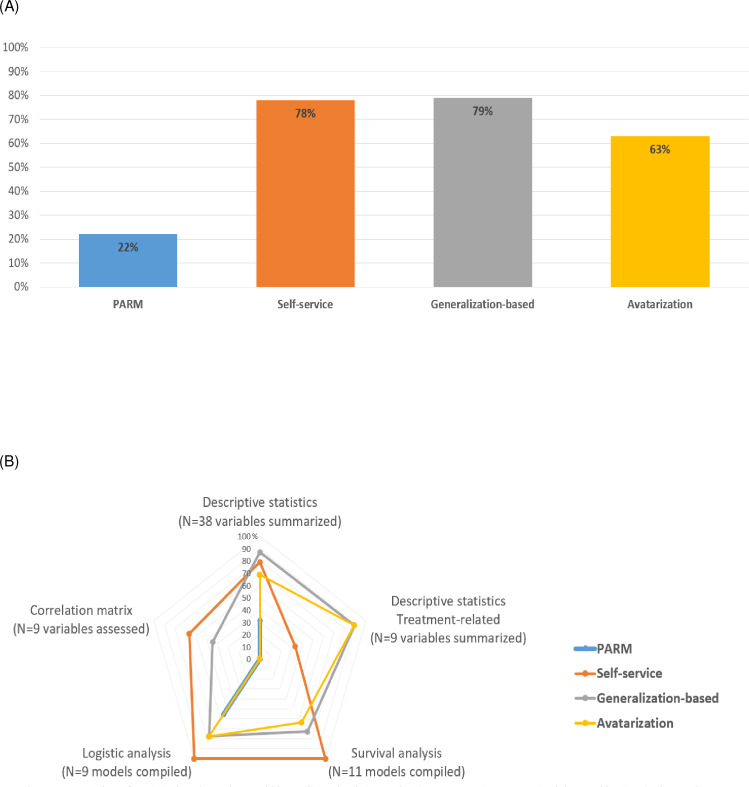
Level 2 score by anonymization method. (A) Level 2 score = Number of statistical analyses that could be replicated utilizing each privacy-preserving anonymized data and having similar results to those from the raw analysis. Level 2 total replication score ranges from 0 item (no statistical item were replicated and similar) to 76 items (all statistical results were replicated and similar), standardized on a scale of 0–100%. (B) Level 2 sub-score are categorized by type of analysis: descriptive statistics (0–38), descriptive statistics treatment-related (0–9), survival analysis (0–11), logistic regression analysis (0–9) and correlation matrix (0–9).

**Level 2 total accuracy score**, which summarizes the differences in results for the replicated statistical analyses between raw and anonymised data, **are shown in Fig 3A**. **The level 2 sub-scores according to each type of analysis are shown in Fig 3B**:

The PARM approach obtained a level 2 total score of 22.4%, reflecting comparable results in only 17 statistical results out of a total of 76 items. Specifically, 31.6% (12 out of 38) for descriptive statistics and 55.6% (5 out of 9) for logistic models showed similarity. With regard to adjuvant treatment, the PARM algorithm had treatments evenly distributed across all modalities, resulting in dissimilarities in treatment description (as shown in Table 1).

**Fig 4:**
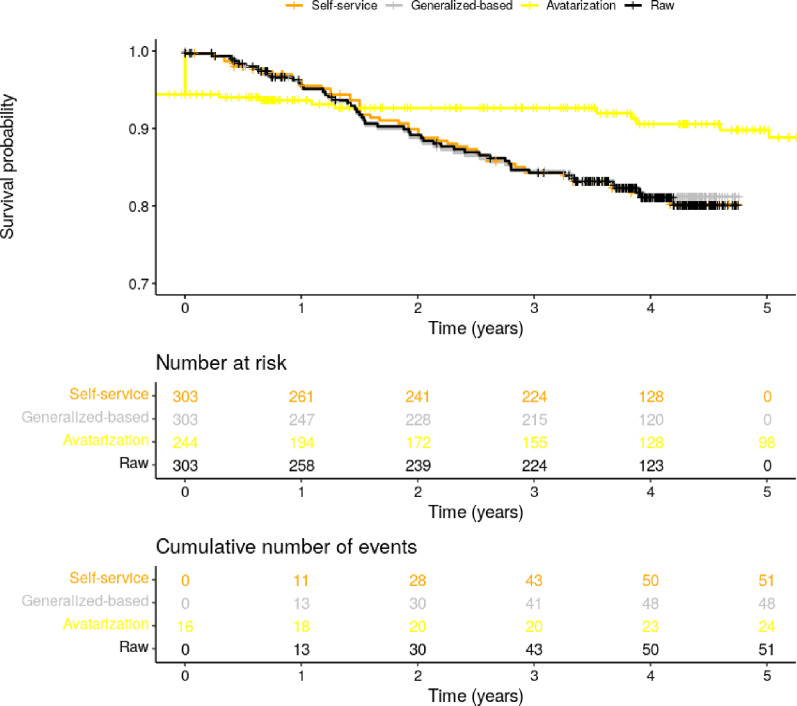
Progression-free survival (PFS) Kaplan-Meier curves by anonymisation method. The Kaplan-Meier curves for progression-free survival (PFS) are presented, along with the cumulative number of patients at risk by year and the cumulative number of patients having experienced an event by year. Note that the PARM method is not displayed in the figure, as the time-to-event calculation was not feasible (the reference start date was empty in the anonymised data).

The avatarization method had a score of 63.2%, with 7 out of 11 (63.6%) similar statistical results for survival analysis but the K-M curves did not fit the expected results. Fig 4 shows the K-M curves generated for each anonymization method, except for the PARM method for which the survival analysis could not be replicated due to missing survival start dates.

The self-service approach had a score of 77.6%, with 100% of similar statistical outputs for survival analysis and logistic models. This result is due to the fact that the source variables used for these analyses were not modified during the anonymization process to preserve the statistical value. Some variables used for treatment-related descriptive analyses were removed from the anonymized data, resulting in the lowest sub-score for this method (33.3%).

The generalization-based approach had the highest score of 79.0%, with 41 out of 47 similar outputs for descriptive statistics. The lowest dimension score was observed for the correlation matrix with a score of 44.4%, mainly due to some data aggregation of disease classification variables.

**Fig 5:**
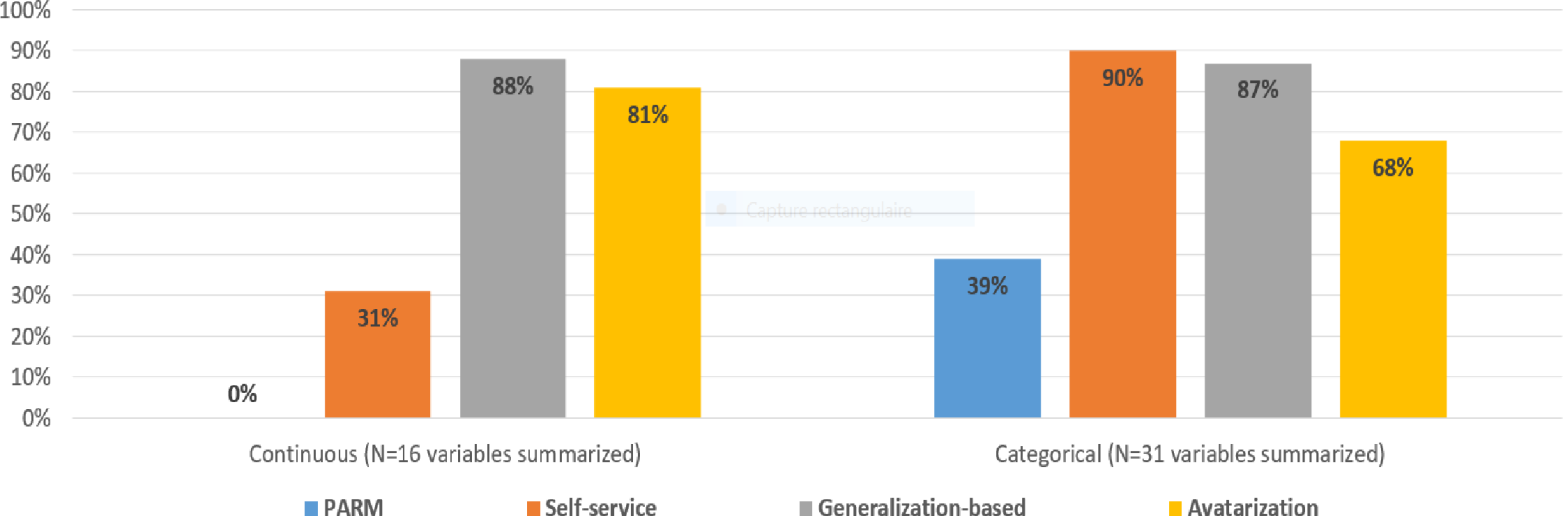
Level 2 descriptive statistics sub-scores by type of variable and anonymization method (%).

Level 2 descriptive statistics sub-scores were also outlined based on the type of variable for the descriptive analyses, including 16 continuous variables and 31 categorical variables. The generalization-based approach achieved a score of 87.5% for continuous variables and 87.1% for categorical variables. The avatarization method achieved 81.3% for continuous variables and 67.7% for categorical variables. The self-service approach achieved 31.3% for continuous variables and 90.3% for categorical variables. The PARM approach achieved 0% for continuous variables and 38.7% for categorical variables (Fig 5). These findings revealed that the preservation of the scientific value of the data for continuous variables was more challenging for the PARM and self-service methods than for the others.

For example, the median (Q1:Q3) Body Mass Index (BMI) ranged from 21 (1:35) to 27 (24–32) in the anonymised data compared to 24 (21:29) in the raw data. When BMI was divided into categories, BMI <25 kg/m^2^ ranged from 31% to 59% compared to 50% for the raw data. The different methods resulted in important differences in the estimation of many variables, as shown in Table 1.

The Kaplan-Meier curves for progression-free survival (PFS) are presented, along with the cumulative number of patients at risk by year and the cumulative number of patients having experienced an event by year. Note that the PARM method is not displayed in the figure, as the time-to-event calculation was not feasible (the reference start date was empty in the anonymised data).

**Level 3 (alteration level)** assessed the extent of data alteration.

**Probability distribution plots** were generated for selected variables, and the Hellinger distance score for variables between the raw and each anonymized solution was summarized using a heat map (see S1 File). The self-service method had a mean score of 0.25. Specifically, 11 variables had a distance score of 0, as these variables were not modified to preserve the statistical value and 4 variables had a score of 1, attributed to abnormal values due to the noise added by this method. The PARM obtained a mean score of 0.15, the generalization-based approach a mean score of 0.09, and the avatarization a mean score of 0.08. It is worth noting that the avatarization method was the only method without extreme scores of 0 and 1 (see Table 2 for a summary of all level scores).

In terms of **data availability**, the self-service method had 43 out of 48 (89.6%) variables with a similar number of patients, the avatarization method had 42 out of 48 (87.5%), the generalization-based approach had 40 out of 48 (83.3%), and the PARM had 14 out of 48 (29.2%). The discrepancy in the avatarization method is only due to variables with a limited number of patients (less than 30), while for generalization-based and self-service the main reason is the presence of empty variables.

**Fig 6:**
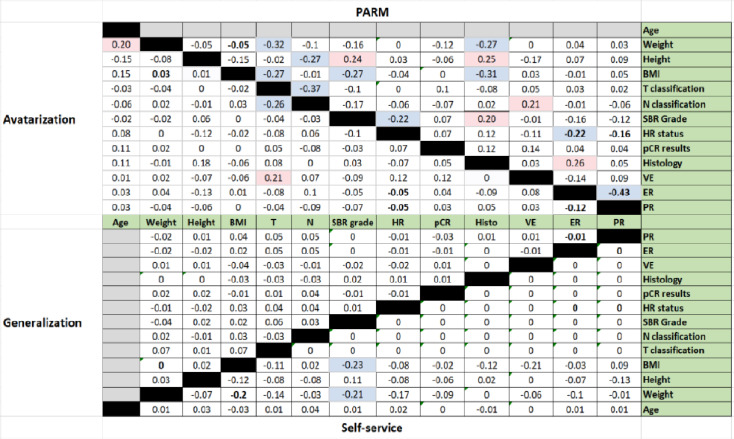
Correlation coefficient difference between each anonymization data and the raw data.

Level 3 also assessed the **relationship between variables** (correlation coefficient and MCA):

The self-service method demonstrated a high degree of similarity for 67 out of 78 Spearman correlation coefficients, corresponding to 85.9% compared to the raw correlation. The avatarization method reported a similar result for 65 out of 78 coefficients (83.3%), the generalization-based approach for 66 out of 78 coefficients (81.8%), and the PARM for 38 out of 78 coefficients (39.4%). Fig 6 illustrates the difference in correlation coefficient between each anonymized data and the raw data.

**Fig 7:**
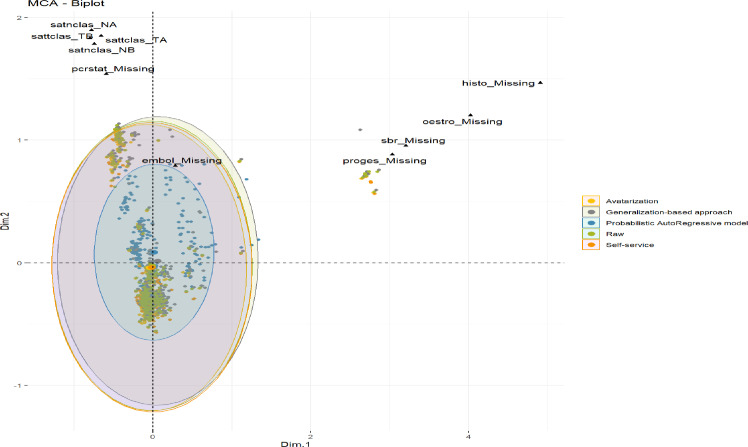
Multiple correspondence analysis (MCA) per anonymization method. Multiple correspondence analysis (MCA) was performed using 12 source variables representing disease characteristics to detect and represent underlying structure differences between raw and each anonymized data (missing data kept as a category for each source variable). This figure is representing the projection of each data (original data in green dots, avatarization data in yellow dots, generalization data in grey dots, self-service data in orange dots) on the first two dimensions of the MCA, showing the original data overlapped with the avatarization, generalization and sefl-service methods, except for the PARM method for which the dots are not aligned. Each data is summarized with its 95% confidence interval around the center of each anonymization method, represented by an ellipse on the two first MCA dimensions. Dim.1 = MCA dimension 1. Dim. 2 = MCA dimension 2. Black shapes show the modalities having the most effect.

By projecting the raw and each anonymized data into the space defined by the first two dimensions derived from the 12 disease characteristic source variables (Fig 7), the MCA showed the extent of each data alteration. The avatarization, generalization-based and self-service approaches showed that the original structure and strong data associations were retained (data overlapped on the Fig 7), in contrast to the PARM method, which significantly altered the data.

**Level 4 (WP29 compliance)** evaluated privacy risks as defined in the WP29 2014 opinion on anonymization techniques, as singling out, linkability and inference.

**For singling out,** out of 219 modalities in the raw data, we observed 34 times a modality with 1 patient (15.5% of all modalities in the data), the self-service method 20 (9.1%), the generalization-based approach 16 (7.3%), the avatarization 14 (6.4%) and the PARM 12 (5.5%). Compared to the raw data, the associated relative risk reductions were 41%, 53%, 59% and 65%, respectively.

**In order to assess linkability,** we considered the global breast cancer landscape in France. In 2018, the incidence of breast cancer recorded 58 459 patients [18]. Approximately 13% of patients are diagnosed with an advanced stage of breast cancer, and surgery is planned in 80% of cases for patients with non-metastatic disease [17,18]. Therefore, approximately 40 687 patients with early breast cancer in France underwent surgery. From the 315 patients included in the cohort, the probability of an inadvertent re-identification if someone with access to the data recognizes someone in the data set, using the following adversary background:

Knowing that a woman in France (25 000 000 women) participated in the cohort, the associated acquaintance probability [19] was 0.0009.

Knowing that a woman with breast cancer in France (n = 228 252/ 5-year prevalence) participated in the cohort, the acquaintance probability was 0.096.

Knowing that a woman with breast cancer in France in 2018 (n = 58 459) participated in the cohort, the acquaintance probability was 0.329.

Knowing that a woman with breast cancer in 2018 who underwent a surgery in France (n = 40 687) participated in the cohort, the acquaintance probability was 0.425.

**For inference,** three parameters with at least five modalities were used: histology of the initial diagnosis had five modalities (k = 5), the k-anonymity probability threshold (1/k) was set at 20%, 6.25% for adjuvant treatment and TNM classification. The number of cases where a modality fell below the threshold was 19 out of 39 (48.7%) for the PARM, 15/39 (38.5%) for the generalization-based approach, 14/39 (35.9%) for the self-service, 12/39 (30.8%) for the avatarization method. When compared to the raw data, the associated relative risk reduction was 5%, 25%, 30% and 40%, respectively.

## Discussion

Whenever possible, data transformations (especially between dates for duration) should be anticipated and performed prior to anonymization. However, as highlighted by James et al. [20], data transformation can remove potentially identifying features from the data, which could affect the utility of the data. This requires a good understanding of the data structure and relationships between variables, which is particularly important when working with longitudinal data.

When planning a time-to-event analysis, it is essential to pay close attention to the variables required for the survival analysis (e.g. date of last contact, date of lost to follow-up, date of event and date of index). The identification of these events of interest prior to anonymization is therefore crucial to perform the necessary derived parameters of survival analysis.

Regarding the four anonymization methods evaluated:

The PARM approach had the lowest scores in all levels and dimensions. This method does not seem to be suitable for longitudinal data. Furthermore, the data structure was completely different from the original data.

The avatarization method had the best preservation of the data structure with the highest score in level 1 (100%), allowing all the expected statistical analyses to be performed. Similar results were found for 34 out of 47 variables (72.3%) for descriptive statistics, however low levels of statistical relevance were observed for survival analysis and correlation between variables.

The self-service method had the highest level of accuracy in statistical results, however 37% of variables were not modified to preserve the statistical value, which raises concern about compliance with GDPR. In addition, the absence of a privacy report with the tool makes it difficult to assess the level of data privacy protection.

The generalization-based approach provided good preservation of the data structure and accurate statistical results, despite the major modification of the birth date which caused degradation of the scores at levels 1 and 2.

Synthetic data generation [20] or other anonymization methods need to consider the development of standardized reports to address concerns about re-identification and to assess utility measurements, according to the criteria of the WP29 (2). In addition to the WP29 criteria, each vendor should also consider the regulatory and ethical implications of their tool/algorithm.

Machanavajjhala et al. showed that k-anonymized data is susceptible to strong attacks due to the lack of diversity in the sensitive attributes. They introduced ℓ-diversity, which provides stronger privacy guarantees [21]. **As it is challenging to assess the loss of utility associated with anonymization methods, a balance must be found between privacy and utility** (without compromising privacy). The level of alteration used in anonymization algorithms should be aligned with the data reuse objectives in question (e.g. open data, internal reuse and reuse by a unique external researcher).

### Limits

Limitations of the project include the selection of anonymization methods, with only four evaluated. Further research could include methods such as Generative Adversarial Networks (GANs) [22]. Another limitation is that without clear requirements on WP29 anonymization indicators, it is difficult to evaluate easily if an anonymized data is safe. Different methods to evaluate compliance exist, such as re-identification methods for categorical variables using a distance-based and probabilistic record linkage [23] or optimization of k-anonymization algorithms as described by Bayardo et al. [24]. However, no consensus has yet been reached on the accepted or approved methods and/or thresholds of anonymization. For level 3, we also evaluated alternative methods for quantifying similarity, including Kullback-Leibler divergence, Hamming distance and Jaccard index. However, these were not retained as they did not align with the variable structure of the data. For singling out in level 4, it could have been beneficial to estimate the probability of re-identification of an individual [16] using quasi-identifier variables such as age and height. However age was not available in two out of four of our anonymized data.

As the project is primarily focused on a single data and limited to four anonymization techniques, raising questions over the generalizability of the findings. There is also a lack of standardized privacy metrics to comprehensively evaluate and compare the effectiveness of the anonymization techniques. Another limitation of our research is that it was limited to replication. In 2016, Scwalbe et al. [25] assessed the statistical challenges in assessing and fostering the reproducibility of scientific results, they defined the following:

The ability of a researcher to replicate the results of a previous study using the same materials as the original researcher. A second researcher could use the same raw data to generate the same analysis files and perform the same statistical analysis to try to obtain the same results.

The replicability as the ability of a researcher to replicate the results of a previous study, using the same procedures but collecting new data.

## Conclusions and recommendations using anonymization for a secondary cohort research

This work provides insight into the statistical and scientific value of real-world data that remained after four different anonymization methods. Descriptive analyses are easier to reproduce, while more complex statistical methods should necessitate a detailed evaluation of the variables of the data before proceeding to anonymization.

Data transformation should be anticipated before anonymization and alterations have to be adjusted for each variable and use case. If the objective is to describe a cohort, minimal requirements must be performed before anonymization. On the other hand, if the objective is to develop a statistical model, it is essential to maintain correlations after anonymization. **A trade-off must be performed between data risk, context risk and data utility to answer the research objective.**

A lack of knowledge in statistical methods and analysis to be performed at the time of anonymization can lead to unreliable results [26]. Therefore, we recommend that researchers identify major analysis describing statistical methods that will enable them to answer secondary research objectives before anonymization is performed. **Standardized privacy metrics should be developed to accurately evaluate the anonymization level of a data**. Currently, anonymization methods are not powerful enough for use in regulatory submissions. However, other potential use cases could be investigated, such as evaluating a subset of a database to better understand which types of analysis that can be performed afterwards on the full raw data. Prior to anonymization, it is essential to have a good understanding of the database structure ensuring that the relationship between variables is maintained. It is also important to have a thorough understanding of the planned analysis, as any new or exploratory analysis not initially planned might not be suitable using an anonymized data. **Once the anonymization is complete, a report should include sufficient information regarding the level of alteration to ensure data utility is preserved for planned analysis. It should also contain a comprehensive privacy risk assessment.**

## Supporting information

S1 File Supplementary data. (ZIP)
