# Supplementary material for: Comparison of anonymization techniques regarding statistical reproducibility
Source: PLOS Digit Health. 2025 Feb 3;4(2):e0000735. doi: 10.1371/journal.pdig.0000735 (PMC11790161; doi:10.1371/journal.pdig.0000735)

KADOR PoC Anonymisation – Analysis report on the generalization anonymized data

A FRENCH RETROSPECTIVE STUDY DESCRIBING THE EPIDEMIOLOGY AND THE THERAPEUTIC MANAGEMENT OF PATIENTS TREATED BY HERCEPTIN® BASED NEOADJUVANT TREATMENT FOR HER2-POSITIVE EARLY BREAST CANCER

Mathieu Boucher

2022-10-25

Table of Contents

[**1 Analysis of study conduct 1**](#_heading=)

[1.1 Patient Disposition 1](#_heading=)

[Table 1.1.1 Summary of patient disposition - Full Analysis Set Population 1](#_heading=)

[Table 1.1.2 Latest news - Among patients not being followed in the site - Full Analysis Set Population 2](#_heading=)

[Table 1.1.3 Cause of death - Among dead patients - Full Analysis Set Population 4](#_heading=)

[Table 1.1.4 Time from diagnostic to progression - Among patients having experienced progression of the disease since the beginning of adjuvant therapy - Full Analysis Set Population 5](#_heading=)

[Table 1.1.5 Dates available - Full Analysis Set Population 6](#_heading=)

[**2 Baseline characteristics 7**](#_heading=)

[2.1 Demographics and baseline disease characteristics 7](#_heading=)

[Table 2.1.1 Summary of demographics and baseline disease characteristics - Full Analysis Set Population 7](#_heading=)

[Table 2.1.2 Summary of demographics and baseline disease characteristics by pCR result - Full Analysis Set Population 12](#_heading=)

[**3 Surgery and pCR 17**](#_heading=)

[3.1 Surgery 17](#_heading=)

[3.1.1 Summary of surgery - Among patients with at least one surgery - Full Analysis Set Population 17](#_heading=)

[3.2 pCR 18](#_heading=)

[3.2.1 Summary of pCR - Full Analysis Set Population 18](#_heading=)

[**4 Adjuvant treatments 20**](#_heading=)

[Table 4.1 Summary of adjuvant treatments - Among subjects with at least one adjuvant treatments - Full Analysis Set Population 20](#_heading=)

[Table 4.2 Summary of adjuvant treatments by adjuvant treatment - Among subjects with at least one adjuvant treatments - Full Analysis Set Population 21](#_heading=)

[Table 4.3 Time between surgery and adjuvant treatment - Among subjects with at least one adjuvant treatments - Full Analysis Set Population 34](#_heading=)

[Table 4.4 Summary of adjuvant treatments by pCR status - Among subjects with at least one adjuvant treatments - Full Analysis Set Population 35](#_heading=)

[Table 4.5 Summary of adjuvant treatments by adjuvant treatment by pCR status - Among subjects with at least one adjuvant treatments - Full Analysis Set Population 36](#_heading=)

[Table 4.6 Time between surgery and adjuvant treatment by pCR status - Among subjects with at least one adjuvant treatments - Full Analysis Set Population 47](#_heading=)

[**5 Efficacy Analyses 48**](#_heading=)

[5.1 Time to event analyses 48](#_heading=)

[Table 5.1.1 Summary of time from herceptin adjuvant treatment to PFS, overall and by pCR result - Kaplan-Meier estimation - Among subjects with herceptin adjuvant treatment start date available - Full Analysis Set Population 48](#_heading=)

[Table 5.1.2 Survival probabilities of time from herceptin adjuvant treatment to PFS, overall and by pCR result - Kaplan-Meier estimation - Among subjects with herceptin adjuvant treatment start date available - Full Analysis Set Population 49](#_heading=)

[Table 5.1.3 Summary of time from herceptin adjuvant treatment to PFS - Kaplan-Meier curve - Among subjects with herceptin adjuvant treatment start date available - Full Analysis Set Population 50](#_heading=)

[Table 5.1.4 Summary of time from herceptin adjuvant treatment to PFS by pCR result - Kaplan-Meier curve - Among subjects with herceptin adjuvant treatment start date available - Full Analysis Set Population 51](#_heading=)

[**6 Exploratory Analyses 52**](#_heading=)

[6.1 Predictive factors for PFS 52](#_heading=)

[Table 6.1.1 PFS - Univariate Cox proportional hazard analysis - Among subjects with herceptin adjuvant treatment start date available - Full Analysis Set Population 52](#_heading=)

[Table 6.1.2 PFS - Multivariate Cox proportional hazard analysis - Among subjects with herceptin adjuvant treatment start date available - Full Analysis Set Population 55](#_heading=)

[6.2 Predictive factors for pCR result 56](#_heading=)

[Table 6.2.1 pCR result - Univariate analysis - Full Analysis Set Population 56](#_heading=)

[Table 6.2.2 pCR result - Multivariate analysis - Full Analysis Set Population 59](#_heading=)

[6.3 Predictive factors for PFS and pCR result 60](#_heading=)

[Table 6.3.1 Correlation matrix - Full Analysis Set Population 60](#_heading=)

[Figure 6.3.2 Correlation coefficient matrix - Full Analysis Set Population 61](#_heading=)

# 1 Analysis of study conduct

## 1.1 Patient Disposition

### Table 1.1.1 Summary of patient disposition - Full Analysis Set Population

| Characteristic | All (N = 315) |
| --- | --- |
| Follow-up duration (years) |  |
| Nobs | 214 |
| Mean (SD) | 4.48 (0.77) |
| Median (Q1;Q3) | 4.6 (4.3; 4.9) |
| Min - Max | 1.1, 5.3 |
| Missing | 101 |
| Is the patient still being followed in the site (as of December 31, 2018)?, n/N (%) |  |
| Yes | 240/294 (81.6%) |
| No | 54/294 (18.4%) |
| Missing | 21 |
| Time from diagnostic to surgery (months) |  |
| Nobs | 246 |
| Mean (SD) | 6.77 (3.48) |
| Median (Q1;Q3) | 6.4 (5.8; 7.2) |
| Min - Max | 4.2, 57.6 |
| Missing | 69 |
| Has there been any progression of the disease since the beginning of adjuvant therapy, n/N (%) |  |
| Yes | 43/290 (14.8%) |
| No | 247/290 (85.2%) |
| Missing | 25 |
| Follow-up duration (years) = (Last consultation date/Death date – initial diagnosis date of breast cancer + 1) / 365.25 | |
| Time from diagnostic to surgery (months) = (Surgery date – initial diagnosis date of breast cancer) / (365.25/12) | |

### Table 1.1.2 Latest news - Among patients not being followed in the site - Full Analysis Set Population

| Characteristic | All (N = 54) |
| --- | --- |
| Latest news |  |
| The patient is dead | 17/52 (32.7%) |
| The patient is lost to follow-up | 31/52 (59.6%) |
| Other reason | 4/52 (7.7%) |
| Missing | 2 |

### Table 1.1.3 Cause of death - Among dead patients - Full Analysis Set Population

| Characteristic | All (N = 17) |
| --- | --- |
| Cause of death |  |
| Disease progression | 10/15 (66.7%) |
| Other reason | 5/15 (33.3%) |
| Missing | 2 |

### Table 1.1.4 Time from diagnostic to progression - Among patients having experienced progression of the disease since the beginning of adjuvant therapy - Full Analysis Set Population

| Characteristic | All (N = 43) |
| --- | --- |
| Time from diagnostic to progression (years) |  |
| Nobs | 35 |
| Mean (SD) | 2.24 (1.02) |
| Median (Q1;Q3) | 2.0 (1.5; 3.0) |
| Min - Max | 0.8, 4.4 |
| Missing | 8 |
| Time from diagnostic to progression (years) = (Date of the first progression of the disease – initial diagnosis date of breast cancer) / 365.25 | |

### Table 1.1.5 Dates available - Full Analysis Set Population

| Characteristic | All (N = 315) |
| --- | --- |
| Initial diagnosis date of breast cancer available |  |
| Yes | 254/315 (80.6%) |
| No | 61/315 (19.4%) |
| Surgery date available |  |
| Yes | 296/315 (94.0%) |
| No | 19/315 (6.0%) |
| Date of last consultation available among patients still being followed in the site (as of December 31, 2018) |  |
| Yes | 240/240 (100.0%) |
| No | 0/240 (0.0%) |
| Date of the first progression of the disease available among patients having experienced progression of the disease since the beginning of adjuvant therapy |  |
| Yes | 43/43 (100.0%) |
| No | 0/43 (0.0%) |
| Death date available among dead patients |  |
| Yes | 17/17 (100.0%) |
| No | 0/17 (0.0%) |

# 2 Baseline characteristics

## 2.1 Demographics and baseline disease characteristics

### Table 2.1.1 Summary of demographics and baseline disease characteristics - Full Analysis Set Population

| Characteristic | All (N = 315) |
| --- | --- |
| BMI (kg/m2), n/N (%) |  |
| <25 | 148/311 (47.6%) |
| [25 - 30[ | 92/311 (29.6%) |
| >=30 | 71/311 (22.8%) |
| Missing | 4 |
| Professional situation, n/N (%) |  |
| Worker | 146/181 (80.7%) |
| Jobless person | 35/181 (19.3%) |
| Missing | 134 |
| Weight (kg) |  |
| Nobs | 312 |
| Mean (SD) | 68.57 (15.50) |
| Median (Q1;Q3) | 65.0 (58.0; 77.0) |
| Min - Max | 40, 126 |
| Missing | 3 |
| Height(cm) |  |
| Nobs | 311 |
| Mean (SD) | 163.05 (5.76) |
| Median (Q1;Q3) | 163.0 (160.0; 167.0) |
| Min - Max | 146, 180 |
| Missing | 4 |
| Weight at initiation of adjuvant therapy (kg) |  |
| Nobs | 262 |
| Mean (SD) | 68.42 (15.31) |
| Median (Q1;Q3) | 67.0 (58.0; 76.0) |
| Min - Max | 40, 126 |
| Missing | 53 |
| Classification T, n/N (%) |  |
| T0 | 1/303 (0.3%) |
| T1a | 4/303 (1.3%) |
| T1b | 1/303 (0.3%) |
| T1c | 19/303 (6.3%) |
| T2 | 161/303 (53.1%) |
| T3 | 72/303 (23.8%) |
| T4a | 11/303 (3.6%) |
| T4b | 5/303 (1.7%) |
| T4c | 3/303 (1.0%) |
| T4d | 23/303 (7.6%) |
| TX | 3/303 (1.0%) |
| Missing | 12 |
| Classification N, n/N (%) |  |
| N0 | 110/300 (36.7%) |
| N1 | 136/300 (45.3%) |
| N2 | 23/300 (7.7%) |
| N3 | 5/300 (1.7%) |
| NX | 26/300 (8.7%) |
| Missing | 15 |
| Histology at the initial diagnosis, n/N (%) |  |
| Invasive ductal carcinoma | 284/306 (92.8%) |
| Invasive lobular carcinoma | 10/306 (3.3%) |
| Mixed carcinoma | 1/306 (0.3%) |
| Other | 9/306 (2.9%) |
| Unknown | 2/306 (0.7%) |
| Missing | 9 |
| Presence of vascular emboli, n/N (%) |  |
| Yes | 25/223 (11.2%) |
| No | 198/223 (88.8%) |
| Missing | 92 |
| SBR grade, n/N (%) |  |
| SBR I | 7/294 (2.4%) |
| SBR II | 138/294 (46.9%) |
| SBR III | 143/294 (48.6%) |
| Ungradable | 6/294 (2.0%) |
| Missing | 21 |
| Number of lymph nodes invaded |  |
| Nobs | 226 |
| Mean (SD) | 0.91 (1.50) |
| Median (Q1;Q3) | 0.0 (0.0; 1.0) |
| Min - Max | 0, 10 |
| Missing | 89 |
| Estrogen receptors, n/N (%) |  |
| positive | 176/303 (58.1%) |
| negative | 127/303 (41.9%) |
| not assessable | 0/303 (0.0%) |
| Missing | 12 |
| Progesterone receptors, n/N (%) |  |
| positive | 124/300 (41.3%) |
| negative | 176/300 (58.7%) |
| not assessable | 0/300 (0.0%) |
| Missing | 15 |
| Hormonal receptors status, n/N (%) |  |
| ER and/or PR + | 181/301 (60.1%) |
| ER and PR - | 120/301 (39.9%) |
| Missing | 14 |

### Table 2.1.2 Summary of demographics and baseline disease characteristics by pCR result - Full Analysis Set Population

| Characteristic | pCR (N = 132) | No pCR (N = 183) |
| --- | --- | --- |
| BMI (kg/m2), n/N (%) |  |  |
| <25 | 65/130 (50.0%) | 83/181 (45.9%) |
| [25 - 30[ | 37/130 (28.5%) | 55/181 (30.4%) |
| >=30 | 28/130 (21.5%) | 43/181 (23.8%) |
| Missing | 2 | 2 |
| Professional situation, n/N (%) |  |  |
| Worker | 62/79 (78.5%) | 84/102 (82.4%) |
| Jobless person | 17/79 (21.5%) | 18/102 (17.6%) |
| Missing | 53 | 81 |
| Weight (kg) |  |  |
| Nobs | 130 | 182 |
| Mean (SD) | 67.54 (14.74) | 69.31 (16.02) |
| Median (Q1;Q3) | 64.0 (58.0; 75.8) | 66.0 (58.2; 78.0) |
| Min - Max | 40, 119 | 45, 126 |
| Missing | 2 | 1 |
| Height(cm) |  |  |
| Nobs | 130 | 181 |
| Mean (SD) | 163.02 (5.29) | 163.08 (6.09) |
| Median (Q1;Q3) | 163.0 (160.0; 166.0) | 162.0 (159.0; 167.0) |
| Min - Max | 146, 180 | 149, 180 |
| Missing | 2 | 2 |
| Weight at initiation of adjuvant therapy (kg) |  |  |
| Nobs | 111 | 151 |
| Mean (SD) | 68.11 (14.79) | 68.65 (15.73) |
| Median (Q1;Q3) | 66.0 (57.0; 75.5) | 67.0 (58.0; 77.0) |
| Min - Max | 40, 121 | 43, 126 |
| Missing | 21 | 32 |
| Classification T, n/N (%) |  |  |
| T0 | 1/124 (0.8%) | 0/179 (0.0%) |
| T1a | 0/124 (0.0%) | 4/179 (2.2%) |
| T1b | 0/124 (0.0%) | 1/179 (0.6%) |
| T1c | 10/124 (8.1%) | 9/179 (5.0%) |
| T2 | 62/124 (50.0%) | 99/179 (55.3%) |
| T3 | 34/124 (27.4%) | 38/179 (21.2%) |
| T4a | 5/124 (4.0%) | 6/179 (3.4%) |
| T4b | 2/124 (1.6%) | 3/179 (1.7%) |
| T4c | 0/124 (0.0%) | 3/179 (1.7%) |
| T4d | 8/124 (6.5%) | 15/179 (8.4%) |
| TX | 2/124 (1.6%) | 1/179 (0.6%) |
| Missing | 8 | 4 |
| Classification N, n/N (%) |  |  |
| N0 | 44/121 (36.4%) | 66/179 (36.9%) |
| N1 | 58/121 (47.9%) | 78/179 (43.6%) |
| N2 | 9/121 (7.4%) | 14/179 (7.8%) |
| N3 | 4/121 (3.3%) | 1/179 (0.6%) |
| NX | 6/121 (5.0%) | 20/179 (11.2%) |
| Missing | 11 | 4 |
| Histology at the initial diagnosis, n/N (%) |  |  |
| Invasive ductal carcinoma | 117/130 (90.0%) | 167/176 (94.9%) |
| Invasive lobular carcinoma | 3/130 (2.3%) | 7/176 (4.0%) |
| Mixed carcinoma | 1/130 (0.8%) | 0/176 (0.0%) |
| Other | 8/130 (6.2%) | 1/176 (0.6%) |
| Unknown | 1/130 (0.8%) | 1/176 (0.6%) |
| Missing | 2 | 7 |
| Presence of vascular emboli, n/N (%) |  |  |
| Yes | 8/100 (8.0%) | 17/123 (13.8%) |
| No | 92/100 (92.0%) | 106/123 (86.2%) |
| Missing | 32 | 60 |
| SBR grade, n/N (%) |  |  |
| SBR I | 1/126 (0.8%) | 6/168 (3.6%) |
| SBR II | 60/126 (47.6%) | 78/168 (46.4%) |
| SBR III | 63/126 (50.0%) | 80/168 (47.6%) |
| Ungradable | 2/126 (1.6%) | 4/168 (2.4%) |
| Missing | 6 | 15 |
| Number of lymph nodes invaded |  |  |
| Nobs | 99 | 127 |
| Mean (SD) | 0.88 (1.49) | 0.93 (1.51) |
| Median (Q1;Q3) | 1.0 (0.0; 1.0) | 0.0 (0.0; 1.0) |
| Min - Max | 0, 9 | 0, 10 |
| Missing | 33 | 56 |
| Estrogen receptors, n/N (%) |  |  |
| positive | 64/128 (50.0%) | 112/175 (64.0%) |
| negative | 64/128 (50.0%) | 63/175 (36.0%) |
| not assessable | 0/128 (0.0%) | 0/175 (0.0%) |
| Missing | 4 | 8 |
| Progesterone receptors, n/N (%) |  |  |
| positive | 45/127 (35.4%) | 79/173 (45.7%) |
| negative | 82/127 (64.6%) | 94/173 (54.3%) |
| not assessable | 0/127 (0.0%) | 0/173 (0.0%) |
| Missing | 5 | 10 |
| Hormonal receptors status, n/N (%) |  |  |
| ER and/or PR + | 67/127 (52.8%) | 114/174 (65.5%) |
| ER and PR - | 60/127 (47.2%) | 60/174 (34.5%) |
| Missing | 5 | 9 |

# 3 Surgery and pCR

## 3.1 Surgery

### 3.1.1 Summary of surgery - Among patients with at least one surgery - Full Analysis Set Population

| Characteristic | All (N = 313) |
| --- | --- |
| At least one Surgery* |  |
| Axillary curage | 249 (79.6%) |
| Mastectomy | 166 (53%) |
| Conservative surgery | 146 (46.6%) |
| Sentinel Ganglion | 53 (16.9%) |
| * One patient can have reported several surgery types | |

## 3.2 pCR

### 3.2.1 Summary of pCR - Full Analysis Set Population

| Characteristic | All (N = 315) |
| --- | --- |
| pCR results* |  |
| pCR | 132/315 (41.9%) |
| No pCR | 183/315 (58.1%) |
| Missing | 0 |
| Absence of invasive and in situ residues in the breast and in the lymph nodes |  |
| Yes | 105/240 (43.8%) |
| No | 135/240 (56.2%) |
| Missing | 75 |
| Absence of invasive residues in the breast and lymph nodes, regardless of the presence of ductal carcinoma in situ |  |
| Yes | 112/240 (46.7%) |
| No | 128/240 (53.3%) |
| Missing | 75 |
| Classification Chevallier |  |
| Grade 1 | 1/7 (14.3%) |
| Grade 2 | 2/7 (28.6%) |
| Grade 3 | 4/7 (57.1%) |
| Missing | 308 |
| Classification Sataloff T |  |
| TA | 32/52 (61.5%) |
| TB | 16/52 (30.8%) |
| TC | 4/52 (7.7%) |
| Missing | 263 |
| Classification Sataloff N |  |
| NA | 21/52 (40.4%) |
| NB | 20/52 (38.5%) |
| NC | 8/52 (15.4%) |
| ND | 3/52 (5.8%) |
| Missing | 263 |
| Classification RCB |  |
| RCB-I | 1/2 (50.0%) |
| RCB-II | 1/2 (50.0%) |
| Missing | 313 |
| * pCR results = pCR if ypT0/Tis ypN0 is ticked Yes OR, Grade 1 or Grade 2 are ticked for Classification Chevallier OR, TA and NA are ticked for Classification Sataloff OR, RCB0 is ticked for Classification RCB | |

# 4 Adjuvant treatments

## Table 4.1 Summary of adjuvant treatments - Among subjects with at least one adjuvant treatments - Full Analysis Set Population

| Characteristic | All (N = 305) |
| --- | --- |
| At least one Adjuvant Treatment* |  |
| Trastuzumab (Herceptin) | 305 (100%) |
| Tamoxifene | 81 (26.6%) |
| Letrozole | 40 (13.1%) |
| Anastrozole | 21 (6.9%) |
| Other | 6 (2%) |
| Exemestane | 5 (1.6%) |
| Other hormonotherapy 1 | 3 (1%) |
| * One patient can have reported several adjuvant treatment types | |

## Table 4.2 Summary of adjuvant treatments by adjuvant treatment - Among subjects with at least one adjuvant treatments - Full Analysis Set Population

| Characteristic | All |
| --- | --- |
| Anastrozole :  - Duration (months) |  |
| Nobs | 0 |
| Mean (SD) | NA (NA) |
| Median (Q1;Q3) | NA (NA; NA) |
| Min - Max | NA, NA |
| Missing | 21 |
| - Administration frequency |  |
| Nobs | 16 |
| Mean (SD) | 4.00 (0.00) |
| Median (Q1;Q3) | 4.0 (4.0; 4.0) |
| Min - Max | 4.0, 4.0 |
| Missing | 5 |
| - Maintenance dose (cycle) |  |
| Nobs | 0 |
| Mean (SD) | NA (NA) |
| Median (Q1;Q3) | NA (NA; NA) |
| Min - Max | NA, NA |
| Missing | 21 |
| - Route of administration, n/N (%) | 0/0 (NA%) |
| Missing | 21 |
| - Number of cycles completed |  |
| Nobs | 0 |
| Mean (SD) | NA (NA) |
| Median (Q1;Q3) | NA (NA; NA) |
| Min - Max | NA, NA |
| Missing | 21 |
| - Location of administration, n/N (%) | 0/0 (NA%) |
| Missing | 21 |
| - Start date of treatment available, n/N (%) |  |
| Yes | 18/21 (85.7%) |
| No | 3/21 (14.3%) |
| - End date of treatment available, n/N (%) |  |
| No | 4/4 (100.0%) |
| Exemestane :  - Duration (months) |  |
| Nobs | 2 |
| Mean (SD) | 9.97 (10.25) |
| Median (Q1;Q3) | 10.0 (6.3; 13.6) |
| Min - Max | 2.7, 17.2 |
| Missing | 3 |
| - Administration frequency |  |
| Nobs | 2 |
| Mean (SD) | 4.00 (0.00) |
| Median (Q1;Q3) | 4.0 (4.0; 4.0) |
| Min - Max | 4.0, 4.0 |
| Missing | 3 |
| - Maintenance dose (cycle) |  |
| Nobs | 0 |
| Mean (SD) | NA (NA) |
| Median (Q1;Q3) | NA (NA; NA) |
| Min - Max | NA, NA |
| Missing | 5 |
| - Route of administration, n/N (%) | 0/0 (NA%) |
| Missing | 5 |
| - Number of cycles completed |  |
| Nobs | 0 |
| Mean (SD) | NA (NA) |
| Median (Q1;Q3) | NA (NA; NA) |
| Min - Max | NA, NA |
| Missing | 5 |
| - Location of administration, n/N (%) | 0/0 (NA%) |
| Missing | 5 |
| - Start date of treatment available, n/N (%) |  |
| Yes | 4/5 (80.0%) |
| No | 1/5 (20.0%) |
| - End date of treatment available, n/N (%) |  |
| No | 1/3 (33.3%) |
| Yes | 2/3 (66.7%) |
| Letrozole :  - Duration (months) |  |
| Nobs | 10 |
| Mean (SD) | 15.67 (12.12) |
| Median (Q1;Q3) | 12.4 (8.7; 15.6) |
| Min - Max | 4.0, 42.7 |
| Missing | 30 |
| - Administration frequency |  |
| Nobs | 24 |
| Mean (SD) | 4.00 (0.00) |
| Median (Q1;Q3) | 4.0 (4.0; 4.0) |
| Min - Max | 4.0, 4.0 |
| Missing | 16 |
| - Maintenance dose (cycle) |  |
| Nobs | 2 |
| Mean (SD) | 2.00 (0.00) |
| Median (Q1;Q3) | 2.0 (2.0; 2.0) |
| Min - Max | 2.0, 2.0 |
| Missing | 38 |
| - Route of administration, n/N (%) | 0/0 (NA%) |
| Missing | 40 |
| - Number of cycles completed |  |
| Nobs | 0 |
| Mean (SD) | NA (NA) |
| Median (Q1;Q3) | NA (NA; NA) |
| Min - Max | NA, NA |
| Missing | 40 |
| - Location of administration, n/N (%) | 0/0 (NA%) |
| Missing | 40 |
| - Start date of treatment available, n/N (%) |  |
| Yes | 39/40 (97.5%) |
| No | 1/40 (2.5%) |
| - End date of treatment available, n/N (%) |  |
| No | 8/18 (44.4%) |
| Yes | 10/18 (55.6%) |
| Other :  - Duration (months) |  |
| Nobs | 3 |
| Mean (SD) | 2.41 (2.06) |
| Median (Q1;Q3) | 3.5 (1.8; 3.6) |
| Min - Max | 0.0, 3.7 |
| Missing | 3 |
| - Administration frequency |  |
| Nobs | 5 |
| Mean (SD) | 2.60 (0.89) |
| Median (Q1;Q3) | 3.0 (3.0; 3.0) |
| Min - Max | 1.0, 3.0 |
| Missing | 1 |
| - Maintenance dose (cycle) |  |
| Nobs | 4 |
| Mean (SD) | 767.50 (1,166.39) |
| Median (Q1;Q3) | 247.5 (75.0; 940.0) |
| Min - Max | 75.0, 2,500.0 |
| Missing | 2 |
| - Route of administration, n/N (%) |  |
| Intravenous | 1/1 (100.0%) |
| Missing | 5 |
| - Number of cycles completed |  |
| Nobs | 4 |
| Mean (SD) | 6.25 (4.50) |
| Median (Q1;Q3) | 6.0 (4.8; 7.5) |
| Min - Max | 1.0, 12.0 |
| Missing | 2 |
| - Location of administration, n/N (%) | 0/0 (NA%) |
| Missing | 6 |
| - Start date of treatment available, n/N (%) |  |
| Yes | 4/6 (66.7%) |
| No | 2/6 (33.3%) |
| - End date of treatment available, n/N (%) |  |
| No | 3/6 (50.0%) |
| Yes | 3/6 (50.0%) |
| Other hormonotherapy 1 :  - Duration (months) |  |
| Nobs | 2 |
| Mean (SD) | 30.46 (13.57) |
| Median (Q1;Q3) | 30.5 (25.7; 35.3) |
| Min - Max | 20.9, 40.0 |
| Missing | 1 |
| - Administration frequency |  |
| Nobs | 1 |
| Mean (SD) | 4.00 (NA) |
| Median (Q1;Q3) | 4.0 (4.0; 4.0) |
| Min - Max | 4.0, 4.0 |
| Missing | 2 |
| - Maintenance dose (cycle) |  |
| Nobs | 0 |
| Mean (SD) | NA (NA) |
| Median (Q1;Q3) | NA (NA; NA) |
| Min - Max | NA, NA |
| Missing | 3 |
| - Route of administration, n/N (%) | 0/0 (NA%) |
| Missing | 3 |
| - Number of cycles completed |  |
| Nobs | 0 |
| Mean (SD) | NA (NA) |
| Median (Q1;Q3) | NA (NA; NA) |
| Min - Max | NA, NA |
| Missing | 3 |
| - Location of administration, n/N (%) | 0/0 (NA%) |
| Missing | 3 |
| - Start date of treatment available, n/N (%) |  |
| Yes | 2/3 (66.7%) |
| No | 1/3 (33.3%) |
| - End date of treatment available, n/N (%) |  |
| No | 1/3 (33.3%) |
| Yes | 2/3 (66.7%) |
| Tamoxifene :  - Duration (months) |  |
| Nobs | 23 |
| Mean (SD) | 21.67 (11.85) |
| Median (Q1;Q3) | 22.1 (10.5; 33.1) |
| Min - Max | 3.0, 43.1 |
| Missing | 58 |
| - Administration frequency |  |
| Nobs | 56 |
| Mean (SD) | 3.95 (0.40) |
| Median (Q1;Q3) | 4.0 (4.0; 4.0) |
| Min - Max | 1.0, 4.0 |
| Missing | 25 |
| - Maintenance dose (cycle) |  |
| Nobs | 14 |
| Mean (SD) | 19.43 (2.14) |
| Median (Q1;Q3) | 20.0 (20.0; 20.0) |
| Min - Max | 12.0, 20.0 |
| Missing | 67 |
| - Route of administration, n/N (%) | 0/0 (NA%) |
| Missing | 81 |
| - Number of cycles completed |  |
| Nobs | 0 |
| Mean (SD) | NA (NA) |
| Median (Q1;Q3) | NA (NA; NA) |
| Min - Max | NA, NA |
| Missing | 81 |
| - Location of administration, n/N (%) | 0/0 (NA%) |
| Missing | 81 |
| - Start date of treatment available, n/N (%) |  |
| Yes | 74/81 (91.4%) |
| No | 7/81 (8.6%) |
| - End date of treatment available, n/N (%) |  |
| No | 19/42 (45.2%) |
| Yes | 23/42 (54.8%) |
| Trastuzumab (Herceptin) :  - Duration (months) |  |
| Nobs | 294 |
| Mean (SD) | 8.21 (2.20) |
| Median (Q1;Q3) | 9.0 (7.6; 9.3) |
| Min - Max | 0.0, 19.6 |
| Missing | 11 |
| - Administration frequency |  |
| Nobs | 248 |
| Mean (SD) | 3.00 (0.00) |
| Median (Q1;Q3) | 3.0 (3.0; 3.0) |
| Min - Max | 3.0, 3.0 |
| Missing | 57 |
| - Maintenance dose (cycle) |  |
| Nobs | 258 |
| Mean (SD) | 6.49 (4.81) |
| Median (Q1;Q3) | 6.0 (6.0; 6.0) |
| Min - Max | 6.0, 67.0 |
| Missing | 47 |
| - Route of administration, n/N (%) |  |
| Both | 41/258 (15.9%) |
| Intravenous | 159/258 (61.6%) |
| Subcutaneous | 58/258 (22.5%) |
| Missing | 47 |
| - Number of cycles completed |  |
| Nobs | 298 |
| Mean (SD) | 13.19 (3.11) |
| Median (Q1;Q3) | 14.0 (12.0; 15.0) |
| Min - Max | 1.0, 20.0 |
| Missing | 7 |
| - Location of administration, n/N (%) |  |
| Home | 6/282 (2.1%) |
| Hospital | 276/282 (97.9%) |
| Missing | 23 |
| - Start date of treatment available, n/N (%) |  |
| Yes | 303/305 (99.3%) |
| No | 2/305 (0.7%) |
| - End date of treatment available, n/N (%) |  |
| No | 10/305 (3.3%) |
| Yes | 295/305 (96.7%) |
| Duration of each adjuvant (months) = (End date of treatment – Start date of treatment + 1) / (365.25/12) | |

## Table 4.3 Time between surgery and adjuvant treatment - Among subjects with at least one adjuvant treatments - Full Analysis Set Population

| Characteristic | All (N = 305) |
| --- | --- |
| Time from surgery to adjuvant treatment initiation of Herceptin (days) |  |
| Nobs | 293 |
| Mean (SD) | 12.83 (39.59) |
| Median (Q1;Q3) | 10.0 (-6.0; 21.0) |
| Min - Max | -183.0, 268.0 |
| Missing | 12 |
| Time from surgery to adjuvant treatment initiation of Herceptin (days) = (Date of adjuvant treatment initiation of Herceptin - Surgery date) | |

## Table 4.4 Summary of adjuvant treatments by pCR status - Among subjects with at least one adjuvant treatments - Full Analysis Set Population

| Characteristic | pCR (N = 130) | No pCR (N = 175) |
| --- | --- | --- |
| At least one Adjuvant Treatment* |  |  |
| Trastuzumab (Herceptin) | 130 (100%) | 175 (100%) |
| Tamoxifene | 31 (23.8%) | 50 (28.6%) |
| Letrozole | 13 (10%) | 27 (15.4%) |
| Anastrozole | 7 (5.4%) | 14 (8%) |
| Other | 3 (2.3%) | 3 (1.7%) |
| Exemestane | 1 (0.8%) | 4 (2.3%) |
| Other hormonotherapy 1 | 0 (0%) | 3 (1.7%) |

## Table 4.5 Summary of adjuvant treatments by adjuvant treatment by pCR status - Among subjects with at least one adjuvant treatments - Full Analysis Set Population

| Characteristic | pCR (N = 130) | No pCR (N = 175) |
| --- | --- | --- |
| Anastrozole :  - Duration (months) |  |  |
| Nobs | 0 | 0 |
| Mean (SD) | NA (NA) | NA (NA) |
| Median (Q1;Q3) | NA (NA; NA) | NA (NA; NA) |
| Min - Max | NA, NA | NA, NA |
| Missing | 7 | 14 |
| - Administration frequency |  |  |
| Nobs | 6 | 10 |
| Mean (SD) | 4.00 (0.00) | 4.00 (0.00) |
| Median (Q1;Q3) | 4.0 (4.0; 4.0) | 4.0 (4.0; 4.0) |
| Min - Max | 4.0, 4.0 | 4.0, 4.0 |
| Missing | 1 | 4 |
| - Maintenance dose (cycle) |  |  |
| Nobs | 0 | 0 |
| Mean (SD) | NA (NA) | NA (NA) |
| Median (Q1;Q3) | NA (NA; NA) | NA (NA; NA) |
| Min - Max | NA, NA | NA, NA |
| Missing | 7 | 14 |
| - Route of administration, n/N (%) | 0/0 (NA%) | 0/0 (NA%) |
| Missing | 7 | 14 |
| - Number of cycles completed |  |  |
| Nobs | 0 | 0 |
| Mean (SD) | NA (NA) | NA (NA) |
| Median (Q1;Q3) | NA (NA; NA) | NA (NA; NA) |
| Min - Max | NA, NA | NA, NA |
| Missing | 7 | 14 |
| - Location of administration, n/N (%) | 0/0 (NA%) | 0/0 (NA%) |
| Missing | 7 | 14 |
| Exemestane :  - Duration (months) |  |  |
| Nobs | 0 | 2 |
| Mean (SD) | NA (NA) | 9.97 (10.25) |
| Median (Q1;Q3) | NA (NA; NA) | 10.0 (6.3; 13.6) |
| Min - Max | NA, NA | 2.7, 17.2 |
| Missing | 1 | 2 |
| - Administration frequency |  |  |
| Nobs | 0 | 2 |
| Mean (SD) | NA (NA) | 4.00 (0.00) |
| Median (Q1;Q3) | NA (NA; NA) | 4.0 (4.0; 4.0) |
| Min - Max | NA, NA | 4.0, 4.0 |
| Missing | 1 | 2 |
| - Maintenance dose (cycle) |  |  |
| Nobs | 0 | 0 |
| Mean (SD) | NA (NA) | NA (NA) |
| Median (Q1;Q3) | NA (NA; NA) | NA (NA; NA) |
| Min - Max | NA, NA | NA, NA |
| Missing | 1 | 4 |
| - Route of administration, n/N (%) | 0/0 (NA%) | 0/0 (NA%) |
| Missing | 1 | 4 |
| - Number of cycles completed |  |  |
| Nobs | 0 | 0 |
| Mean (SD) | NA (NA) | NA (NA) |
| Median (Q1;Q3) | NA (NA; NA) | NA (NA; NA) |
| Min - Max | NA, NA | NA, NA |
| Missing | 1 | 4 |
| - Location of administration, n/N (%) | 0/0 (NA%) | 0/0 (NA%) |
| Missing | 1 | 4 |
| Letrozole :  - Duration (months) |  |  |
| Nobs | 4 | 6 |
| Mean (SD) | 18.64 (16.44) | 13.69 (9.52) |
| Median (Q1;Q3) | 13.1 (11.1; 20.6) | 11.3 (8.7; 15.3) |
| Min - Max | 5.7, 42.7 | 4.0, 31.2 |
| Missing | 9 | 21 |
| - Administration frequency |  |  |
| Nobs | 9 | 15 |
| Mean (SD) | 4.00 (0.00) | 4.00 (0.00) |
| Median (Q1;Q3) | 4.0 (4.0; 4.0) | 4.0 (4.0; 4.0) |
| Min - Max | 4.0, 4.0 | 4.0, 4.0 |
| Missing | 4 | 12 |
| - Maintenance dose (cycle) |  |  |
| Nobs | 2 | 0 |
| Mean (SD) | 2.00 (0.00) | NA (NA) |
| Median (Q1;Q3) | 2.0 (2.0; 2.0) | NA (NA; NA) |
| Min - Max | 2.0, 2.0 | NA, NA |
| Missing | 11 | 27 |
| - Route of administration, n/N (%) | 0/0 (NA%) | 0/0 (NA%) |
| Missing | 13 | 27 |
| - Number of cycles completed |  |  |
| Nobs | 0 | 0 |
| Mean (SD) | NA (NA) | NA (NA) |
| Median (Q1;Q3) | NA (NA; NA) | NA (NA; NA) |
| Min - Max | NA, NA | NA, NA |
| Missing | 13 | 27 |
| - Location of administration, n/N (%) | 0/0 (NA%) | 0/0 (NA%) |
| Missing | 13 | 27 |
| Other :  - Duration (months) |  |  |
| Nobs | 1 | 2 |
| Mean (SD) | 0.03 (NA) | 3.60 (0.16) |
| Median (Q1;Q3) | 0.0 (0.0; 0.0) | 3.6 (3.5; 3.7) |
| Min - Max | 0.0, 0.0 | 3.5, 3.7 |
| Missing | 2 | 1 |
| - Administration frequency |  |  |
| Nobs | 3 | 2 |
| Mean (SD) | 2.33 (1.15) | 3.00 (0.00) |
| Median (Q1;Q3) | 3.0 (2.0; 3.0) | 3.0 (3.0; 3.0) |
| Min - Max | 1.0, 3.0 | 3.0, 3.0 |
| Missing | 0 | 1 |
| - Maintenance dose (cycle) |  |  |
| Nobs | 2 | 2 |
| Mean (SD) | 247.50 (243.95) | 1,287.50 (1,714.73) |
| Median (Q1;Q3) | 247.5 (161.2; 333.8) | 1,287.5 (681.2; 1,893.8) |
| Min - Max | 75.0, 420.0 | 75.0, 2,500.0 |
| Missing | 1 | 1 |
| - Route of administration, n/N (%) |  |  |
| Intravenous | 1/1 (100.0%) | 0/0 (NA%) |
| Missing | 2 | 3 |
| - Number of cycles completed |  |  |
| Nobs | 2 | 2 |
| Mean (SD) | 6.50 (7.78) | 6.00 (0.00) |
| Median (Q1;Q3) | 6.5 (3.8; 9.2) | 6.0 (6.0; 6.0) |
| Min - Max | 1.0, 12.0 | 6.0, 6.0 |
| Missing | 1 | 1 |
| - Location of administration, n/N (%) | 0/0 (NA%) | 0/0 (NA%) |
| Missing | 3 | 3 |
| Other hormonotherapy 1 :  - Duration (months) |  |  |
| Nobs | 0 | 2 |
| Mean (SD) | NA (NA) | 30.46 (13.57) |
| Median (Q1;Q3) | NA (NA; NA) | 30.5 (25.7; 35.3) |
| Min - Max | NA, NA | 20.9, 40.0 |
| Missing | 0 | 1 |
| - Administration frequency |  |  |
| Nobs | 0 | 1 |
| Mean (SD) | NA (NA) | 4.00 (NA) |
| Median (Q1;Q3) | NA (NA; NA) | 4.0 (4.0; 4.0) |
| Min - Max | NA, NA | 4.0, 4.0 |
| Missing | 0 | 2 |
| - Maintenance dose (cycle) |  |  |
| Nobs | 0 | 0 |
| Mean (SD) | NA (NA) | NA (NA) |
| Median (Q1;Q3) | NA (NA; NA) | NA (NA; NA) |
| Min - Max | NA, NA | NA, NA |
| Missing | 0 | 3 |
| - Route of administration, n/N (%) | 0/0 (NA%) | 0/0 (NA%) |
| Missing | 0 | 3 |
| - Number of cycles completed |  |  |
| Nobs | 0 | 0 |
| Mean (SD) | NA (NA) | NA (NA) |
| Median (Q1;Q3) | NA (NA; NA) | NA (NA; NA) |
| Min - Max | NA, NA | NA, NA |
| Missing | 0 | 3 |
| - Location of administration, n/N (%) | 0/0 (NA%) | 0/0 (NA%) |
| Missing | 0 | 3 |
| Tamoxifene :  - Duration (months) |  |  |
| Nobs | 11 | 12 |
| Mean (SD) | 25.02 (10.16) | 18.59 (12.87) |
| Median (Q1;Q3) | 24.1 (18.3; 35.3) | 13.0 (9.9; 25.8) |
| Min - Max | 9.6, 38.1 | 3.0, 43.1 |
| Missing | 20 | 38 |
| - Administration frequency |  |  |
| Nobs | 20 | 36 |
| Mean (SD) | 4.00 (0.00) | 3.92 (0.50) |
| Median (Q1;Q3) | 4.0 (4.0; 4.0) | 4.0 (4.0; 4.0) |
| Min - Max | 4.0, 4.0 | 1.0, 4.0 |
| Missing | 11 | 14 |
| - Maintenance dose (cycle) |  |  |
| Nobs | 10 | 4 |
| Mean (SD) | 20.00 (0.00) | 18.00 (4.00) |
| Median (Q1;Q3) | 20.0 (20.0; 20.0) | 20.0 (18.0; 20.0) |
| Min - Max | 20.0, 20.0 | 12.0, 20.0 |
| Missing | 21 | 46 |
| - Route of administration, n/N (%) | 0/0 (NA%) | 0/0 (NA%) |
| Missing | 31 | 50 |
| - Number of cycles completed |  |  |
| Nobs | 0 | 0 |
| Mean (SD) | NA (NA) | NA (NA) |
| Median (Q1;Q3) | NA (NA; NA) | NA (NA; NA) |
| Min - Max | NA, NA | NA, NA |
| Missing | 31 | 50 |
| - Location of administration, n/N (%) | 0/0 (NA%) | 0/0 (NA%) |
| Missing | 31 | 50 |
| Trastuzumab (Herceptin) :  - Duration (months) |  |  |
| Nobs | 126 | 168 |
| Mean (SD) | 8.05 (2.27) | 8.32 (2.15) |
| Median (Q1;Q3) | 8.5 (6.9; 9.2) | 9.0 (7.7; 9.5) |
| Min - Max | 0.0, 19.6 | 0.7, 13.9 |
| Missing | 4 | 7 |
| - Administration frequency |  |  |
| Nobs | 104 | 144 |
| Mean (SD) | 3.00 (0.00) | 3.00 (0.00) |
| Median (Q1;Q3) | 3.0 (3.0; 3.0) | 3.0 (3.0; 3.0) |
| Min - Max | 3.0, 3.0 | 3.0, 3.0 |
| Missing | 26 | 31 |
| - Maintenance dose (cycle) |  |  |
| Nobs | 110 | 148 |
| Mean (SD) | 7.04 (7.31) | 6.08 (0.61) |
| Median (Q1;Q3) | 6.0 (6.0; 6.0) | 6.0 (6.0; 6.0) |
| Min - Max | 6.0, 67.0 | 6.0, 12.0 |
| Missing | 20 | 27 |
| - Route of administration, n/N (%) |  |  |
| Both | 17/109 (15.6%) | 24/149 (16.1%) |
| Intravenous | 66/109 (60.6%) | 93/149 (62.4%) |
| Subcutaneous | 26/109 (23.9%) | 32/149 (21.5%) |
| Missing | 21 | 26 |
| - Number of cycles completed |  |  |
| Nobs | 127 | 171 |
| Mean (SD) | 12.81 (3.38) | 13.48 (2.87) |
| Median (Q1;Q3) | 13.0 (12.0; 15.0) | 14.0 (12.0; 15.0) |
| Min - Max | 1.0, 20.0 | 2.0, 20.0 |
| Missing | 3 | 4 |
| - Location of administration, n/N (%) |  |  |
| Home | 3/120 (2.5%) | 3/162 (1.9%) |
| Hospital | 117/120 (97.5%) | 159/162 (98.1%) |
| Missing | 10 | 13 |
| Duration of each adjuvant (months) = (End date of treatment – Start date of treatment + 1) / (365.25/12) | | |

## Table 4.6 Time between surgery and adjuvant treatment by pCR status - Among subjects with at least one adjuvant treatments - Full Analysis Set Population

| Characteristic | pCR (N = 130) | No pCR (N = 175) |
| --- | --- | --- |
| Time from surgery to adjuvant treatment initiation of Herceptin (days) |  |  |
| Nobs | 126 | 167 |
| Mean (SD) | 15.88 (42.84) | 10.53 (36.92) |
| Median (Q1;Q3) | 9.5 (1.2; 20.0) | 11.0 (-6.0; 21.0) |
| Min - Max | -105.0, 268.0 | -183.0, 189.0 |
| Missing | 4 | 8 |
| Time from surgery to adjuvant treatment initiation of Herceptin (days) = (Date of adjuvant treatment initiation of Herceptin - Surgery date) | | |

# 5 Efficacy Analyses

## 5.1 Time to event analyses

### Table 5.1.1 Summary of time from herceptin adjuvant treatment to PFS, overall and by pCR result - Kaplan-Meier estimation - Among subjects with herceptin adjuvant treatment start date available - Full Analysis Set Population

| PFS | N | Number of event | 10% Percentile (95% CI) |
| --- | --- | --- | --- |
| Overall | 303 | 48 | 1.7 (1.3, 2.5) |
| pCR results* |  |  |  |
| pCR | 130 | 16 | 2.1 (1.5, —) |
| No pCR | 173 | 32 | 1.5 (1.0, 2.3) |
| * pCR results = pCR if ypT0/Tis ypN0 is ticked Yes OR, Grade 1 or Grade 2 are ticked for Classification Chevallier OR, TA and NA are ticked for Classification Sataloff OR, RCB0 is ticked for Classification RCB | | | |
| Patients who did not experience event were censored at their last consultation date. If this date is missing, they were censored at the last adjuvant treatment date | | | |

### Table 5.1.2 Survival probabilities of time from herceptin adjuvant treatment to PFS, overall and by pCR result - Kaplan-Meier estimation - Among subjects with herceptin adjuvant treatment start date available - Full Analysis Set Population

| PFS | N | At 1 year (95% CI) | At 2 years (95% CI) | At 3 years (95% CI) | At 4 years (95% CI) |
| --- | --- | --- | --- | --- | --- |
| Overall | 303 | 95.4 (92.2, 97.3) | 88.8 (84.3, 92.0) | 84.5 (79.5, 88.3) | 81.2 (75.8, 85.5) |
| pCR results* |  |  |  |  |  |
| pCR | 130 | 95.8 (90.3, 98.3) | 90.5 (83.4, 94.6) | 88.7 (81.2, 93.3) | 85.1 (76.6, 90.7) |
| No pCR | 173 | 95.1 (90.4, 97.5) | 87.5 (81.1, 91.9) | 81.3 (74.1, 86.7) | 78.2 (70.6, 84.1) |
| * pCR results = pCR if ypT0/Tis ypN0 is ticked Yes OR, Grade 1 or Grade 2 are ticked for Classification Chevallier OR, TA and NA are ticked for Classification Sataloff OR, RCB0 is ticked for Classification RCB | | | | | |
| Patients who did not experience event were censored at their last consultation date. If this date is missing, they were censored at the last adjuvant treatment date | | | | | |

### Table 5.1.3 Summary of time from herceptin adjuvant treatment to PFS - Kaplan-Meier curve - Among subjects with herceptin adjuvant treatment start date available - Full Analysis Set Population


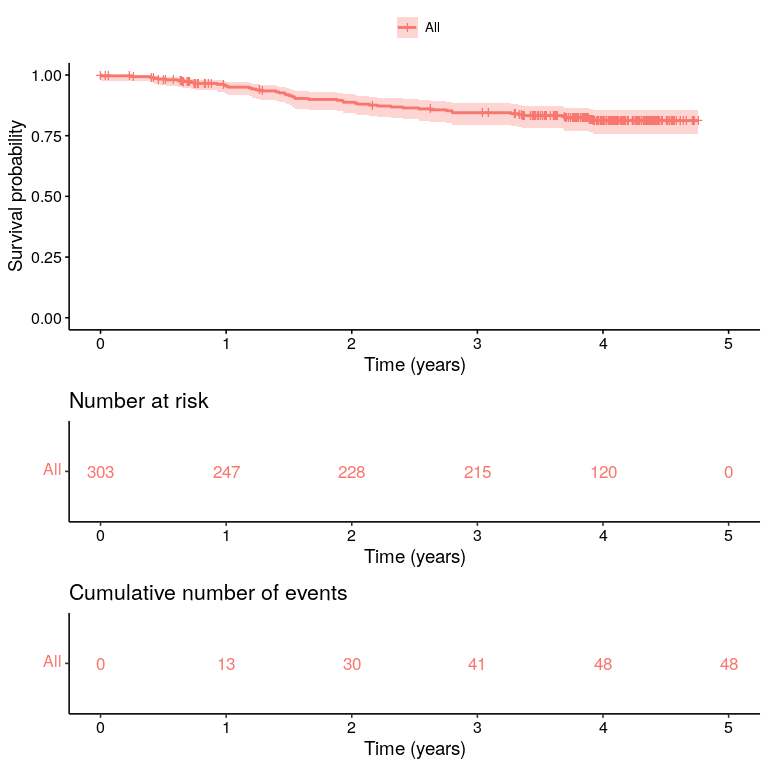


### Table 5.1.4 Summary of time from herceptin adjuvant treatment to PFS by pCR result - Kaplan-Meier curve - Among subjects with herceptin adjuvant treatment start date available - Full Analysis Set Population


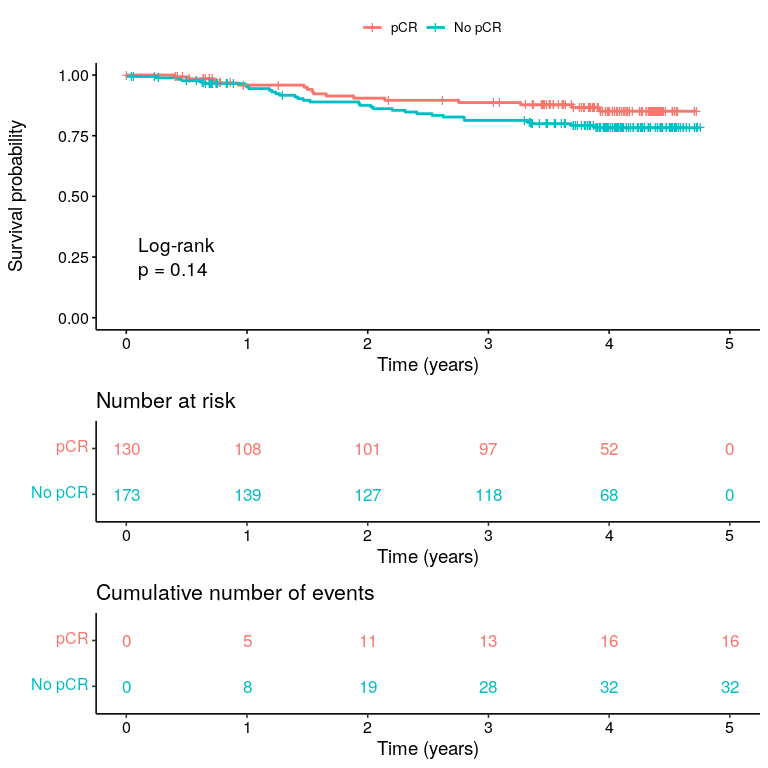


# 6 Exploratory Analyses

## 6.1 Predictive factors for PFS

### Table 6.1.1 PFS - Univariate Cox proportional hazard analysis - Among subjects with herceptin adjuvant treatment start date available - Full Analysis Set Population

|  | Descriptive statistics | | HR and 95% CI | | |
| --- | --- | --- | --- | --- | --- |
| Characteristic | Event, N = 48^1^ | No event, N = 255^1^ | HR^2^ | 95% CI^2^ | p-value |
| BMI (kg/m2) |  |  |  |  | 0.857 |
| <25 | 23/140 (16.4%) | 117/140 (83.6%) | — | — |  |
| [25 - 30[ | 15/90 (16.7%) | 75/90 (83.3%) | 1.04 | 0.54, 2.00 |  |
| >=30 | 9/69 (13.0%) | 60/69 (87.0%) | 0.83 | 0.39, 1.80 |  |
| Missing | 1 | 3 |  |  |  |
| T classification |  |  |  |  | 0.063 |
| T0-3 | 35/249 (14.1%) | 214/249 (85.9%) | — | — |  |
| T>3 | 10/39 (25.6%) | 29/39 (74.4%) | 1.95 | 0.97, 3.94 |  |
| Missing | 3 | 12 |  |  |  |
| N classification |  |  |  |  | 0.357 |
| N0 | 14/105 (13.3%) | 91/105 (86.7%) | — | — |  |
| N1 | 24/132 (18.2%) | 108/132 (81.8%) | 1.46 | 0.76, 2.83 |  |
| N2&N3 | 6/27 (22.2%) | 21/27 (77.8%) | 1.87 | 0.72, 4.88 |  |
| Missing | 4 | 35 |  |  |  |
| SBR Grade |  |  |  |  | 0.677 |
| SBR I & II | 21/142 (14.8%) | 121/142 (85.2%) | — | — |  |
| SBR III | 24/143 (16.8%) | 119/143 (83.2%) | 1.13 | 0.63, 2.03 |  |
| Missing | 3 | 15 |  |  |  |
| Presence of vascular emboli |  |  |  |  | <0.001 |
| Yes | 8/25 (32.0%) | 17/25 (68.0%) | — | — |  |
| No | 25/197 (12.7%) | 172/197 (87.3%) | 0.25 | 0.11, 0.57 |  |
| Missing | 15 | 66 |  |  |  |
| Hormonal receptors status |  |  |  |  | 0.690 |
| ER and/or PR + | 27/179 (15.1%) | 152/179 (84.9%) | — | — |  |
| ER and PR - | 20/119 (16.8%) | 99/119 (83.2%) | 1.13 | 0.63, 2.01 |  |
| Missing | 1 | 4 |  |  |  |
| pCR results* |  |  |  |  | 0.141 |
| pCR | 16/130 (12.3%) | 114/130 (87.7%) | — | — |  |
| No pCR | 32/173 (18.5%) | 141/173 (81.5%) | 1.57 | 0.86, 2.86 |  |
| Missing | 0 | 0 |  |  |  |
| * pCR results = pCR if ypT0/Tis ypN0 is ticked Yes OR, Grade 1 or Grade 2 are ticked for Classification Chevallier OR, TA and NA are ticked for Classification Sataloff OR, RCB0 is ticked for Classification RCB | | | | | |
| Univariate analysis has been done using a cox model. P-value is based on a global wald test from Cox model | | | | | |
| ^1^n/N (%) | | | | | |
| ^2^HR = Hazard Ratio, CI = Confidence Interval | | | | | |

### Table 6.1.2 PFS - Multivariate Cox proportional hazard analysis - Among subjects with herceptin adjuvant treatment start date available - Full Analysis Set Population

|  | Descriptive statistics | | HR and 95% CI | | |
| --- | --- | --- | --- | --- | --- |
| Characteristic | Event, N = 48 | No event, N = 255 | HR^1^ | 95% CI^1^ | p-value |
| Presence of vascular emboli |  |  |  |  |  |
| Yes | 8/25 (32.0%) | 17/25 (68.0%) | — | — |  |
| No | 25/197 (12.7%) | 172/197 (87.3%) | 0.25 | 0.11, 0.57 | <0.001 |
| Missing | 15 | 66 |  |  |  |
| * pCR results = pCR if ypT0/Tis ypN0 is ticked Yes OR, Grade 1 or Grade 2 are ticked for Classification Chevallier OR, TA and NA are ticked for Classification Sataloff OR, RCB0 is ticked for Classification RCB | | | | | |
| Multivariate analysis has been done using a cox model. For covariate with 2 modalities, p-value is based on a global wald test from Cox model, otherwise it is the likelihood ratio test global p-value. The model has been constructed using a stepwise selection of covariates with 0.15 as entry threshold and 0.15 as the retention threshold. | | | | | |
| ^1^HR = Hazard Ratio, CI = Confidence Interval | | | | | |

## 6.2 Predictive factors for pCR result

### Table 6.2.1 pCR result - Univariate analysis - Full Analysis Set Population

|  | Descriptive statistics | | OR and 95% CI | | |
| --- | --- | --- | --- | --- | --- |
| Characteristic | pCR, N = 132^1^ | No pCR, N = 183^1^ | OR^2^ | 95% CI^2^ | p-value |
| BMI (kg/m2) |  |  |  |  | 0.573 |
| <25 | 65/148 (43.9%) | 83/148 (56.1%) | — | — |  |
| [25 - 30[ | 37/92 (40.2%) | 55/92 (59.8%) | 0.86 | 0.50, 1.45 |  |
| >=30 | 28/71 (39.4%) | 43/71 (60.6%) | 0.83 | 0.46, 1.47 |  |
| Missing | 2 | 2 |  |  |  |
| T classification |  |  |  |  | 0.482 |
| T0-3 | 107/258 (41.5%) | 151/258 (58.5%) | — | — |  |
| T>3 | 15/42 (35.7%) | 27/42 (64.3%) | 0.78 | 0.39, 1.53 |  |
| Missing | 10 | 5 |  |  |  |
| N classification |  |  |  |  | 0.675 |
| N0 | 44/110 (40.0%) | 66/110 (60.0%) | — | — |  |
| N1 | 58/136 (42.6%) | 78/136 (57.4%) | 1.12 | 0.67, 1.86 |  |
| N2&N3 | 13/28 (46.4%) | 15/28 (53.6%) | 1.30 | 0.56, 3.00 |  |
| Missing | 17 | 24 |  |  |  |
| SBR Grade |  |  |  |  | 0.734 |
| SBR I & II | 61/145 (42.1%) | 84/145 (57.9%) | — | — |  |
| SBR III | 63/143 (44.1%) | 80/143 (55.9%) | 1.08 | 0.68, 1.73 |  |
| Missing | 8 | 19 |  |  |  |
| Presence of vascular emboli |  |  |  |  | 0.175 |
| Yes | 8/25 (32.0%) | 17/25 (68.0%) | — | — |  |
| No | 92/198 (46.5%) | 106/198 (53.5%) | 1.84 | 0.78, 4.70 |  |
| Missing | 32 | 60 |  |  |  |
| Hormonal receptors status |  |  |  |  | 0.026 |
| ER and/or PR + | 67/181 (37.0%) | 114/181 (63.0%) | — | — |  |
| ER and PR - | 60/120 (50.0%) | 60/120 (50.0%) | 1.70 | 1.07, 2.72 |  |
| Missing | 5 | 9 |  |  |  |
| Univariate analysis has been done using a logistic model. P-value is based on a global wald test from logistic model | | | | | |
| ^1^n/N (%) | | | | | |
| ^2^OR = Odds Ratio, CI = Confidence Interval | | | | | |

### Table 6.2.2 pCR result - Multivariate analysis - Full Analysis Set Population

|  | Descriptive statistics | | OR and 95% CI | | |
| --- | --- | --- | --- | --- | --- |
| Characteristic | pCR, N = 132 | No pCR, N = 183 | OR^1^ | 95% CI^1^ | p-value |
| Hormonal receptors status |  |  |  |  | 0.119 |
| ER and/or PR + | 67/181 (37.0%) | 114/181 (63.0%) | — | — |  |
| ER and PR - | 60/120 (50.0%) | 60/120 (50.0%) | 1.55 | 0.89, 2.71 |  |
| Missing | 5 | 9 |  |  |  |
| Presence of vascular emboli |  |  |  |  | 0.138 |
| Yes | 8/25 (32.0%) | 17/25 (68.0%) | — | — |  |
| No | 92/198 (46.5%) | 106/198 (53.5%) | 1.97 | 0.83, 5.06 |  |
| Missing | 32 | 60 |  |  |  |
| Multivariate analysis has been done using a logistic model. P-value is based on a global wald test from logisitc model. The model has been constructed using a stepwise selection of covariates with 0.15 as entry threshold and 0.15 as the retention threshold. | | | | | |
| ^1^OR = Odds Ratio, CI = Confidence Interval | | | | | |

## 6.3 Predictive factors for PFS and pCR result

### Table 6.3.1 Correlation matrix - Full Analysis Set Population

| Variables | BMI (kg/m2) | T classification | N classification | SBR Grade | Presence of vascular emboli | Hormonal receptors status | pCR results |
| --- | --- | --- | --- | --- | --- | --- | --- |
| BMI (kg/m2) | ND |  |  |  |  |  |  |
| T classification | 0 | ND |  |  |  |  |  |
| N classification | 0.1417 | 5e-04 | ND |  |  |  |  |
| SBR Grade | 0.0975 | 0.9619 | 0.152 | ND |  |  |  |
| Presence of vascular emboli | 0.5297 | 0.1036 | 0.0235 | 0.9192 | ND |  |  |
| Hormonal receptors status | 0.2681 | 0.1338 | 0.1824 | 7e-04 | 0.3928 | ND |  |
| pCR results | 0.7669 | 0.5925 | 0.8068 | 0.8247 | 0.2473 | 0.0345 | ND |
| * pCR results = pCR if ypT0/Tis ypN0 is ticked Yes OR, Grade 1 or Grade 2 are ticked for Classification Chevallier OR, TA and NA are ticked for Classification Sataloff OR, RCB0 is ticked for Classification RCB | | | | | | | |
| ND: Not Done | | | | | | | |
| Between quantitative and qualitative variables: Anova have been used: the p-value displayed is the p-value of the Type 3 test of fixed effects. P-value is displayed in the above table. | | | | | | | |
| Between qualitative variables: Chi² test has been used when all expected counts are >= 5. Otherwise, the Fisher exact test has been used. P-value is displayed in the above table | | | | | | | |

### Figure 6.3.2 Correlation coefficient matrix - Full Analysis Set Population


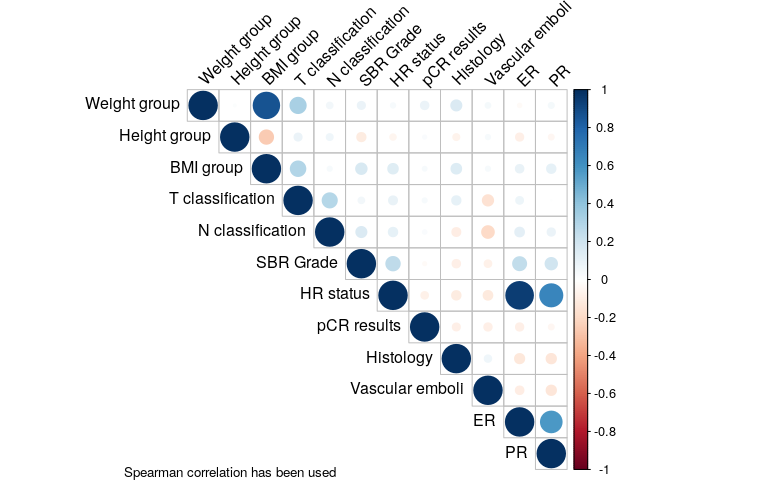

Supplement: S1 File — (ZIP) [file pdig.0000735.s001.zip › Suppl materials generalization_statistical_report.docx]
