# Supplementary material for: Comparison of anonymization techniques regarding statistical reproducibility
Source: PLOS Digit Health. 2025 Feb 3;4(2):e0000735. doi: 10.1371/journal.pdig.0000735 (PMC11790161; doi:10.1371/journal.pdig.0000735)

KADOR PoC Anonymisation – Analysis report on the self service anonymized data

A FRENCH RETROSPECTIVE STUDY DESCRIBING THE EPIDEMIOLOGY AND THE THERAPEUTIC MANAGEMENT OF PATIENTS TREATED BY HERCEPTIN® BASED NEOADJUVANT TREATMENT FOR HER2-POSITIVE EARLY BREAST CANCER

Laetitia Vinet (KLS)

2022-10-25

Table of Contents

[**1 Analysis of study conduct 1**](#_heading=)

[1.1 Patient Disposition 1](#_heading=)

[Table 1.1.1 Summary of patient disposition - Full Analysis Set Population 1](#_heading=)

[Table 1.1.2 Latest news - Among patients not being followed in the site - Full Analysis Set Population 2](#_heading=)

[Table 1.1.3 Cause of death - Among dead patients - Full Analysis Set Population 4](#_heading=)

[Table 1.1.4 Time from diagnostic to progression - Among patients having experienced progression of the disease since the beginning of adjuvant therapy - Full Analysis Set Population 5](#_heading=)

[Table 1.1.5 Dates available - Full Analysis Set Population 6](#_heading=)

[**2 Baseline characteristics 8**](#_heading=)

[2.1 Demographics and baseline disease characteristics 8](#_heading=)

[Table 2.1.1 Summary of demographics and baseline disease characteristics - Full Analysis Set Population 8](#_heading=)

[Table 2.1.2 Summary of demographics and baseline disease characteristics by pCR result - Full Analysis Set Population 13](#_heading=)

[**3 Surgery and pCR 19**](#_heading=)

[3.1 Surgery 19](#_heading=)

[3.1.1 Summary of surgery - Among patients with at least one surgery - Full Analysis Set Population 19](#_heading=)

[3.2 pCR 20](#_heading=)

[3.2.1 Summary of pCR - Full Analysis Set Population 20](#_heading=)

[**4 Adjuvant treatments 22**](#_heading=)

[Table 4.1 Summary of adjuvant treatments - Among subjects with at least one adjuvant treatments - Full Analysis Set Population 22](#_heading=)

[Table 4.2 Summary of adjuvant treatments by adjuvant treatment - Among subjects with at least one adjuvant treatments - Full Analysis Set Population 23](#_heading=)

[Table 4.3 Time between surgery and adjuvant treatment - Among subjects with at least one adjuvant treatments - Full Analysis Set Population 33](#_heading=)

[Table 4.4 Summary of adjuvant treatments by pCR status - Among subjects with at least one adjuvant treatments - Full Analysis Set Population 34](#_heading=)

[Table 4.5 Summary of adjuvant treatments by adjuvant treatment by pCR status - Among subjects with at least one adjuvant treatments - Full Analysis Set Population 35](#_heading=)

[Table 4.6 Time between surgery and adjuvant treatment by pCR status - Among subjects with at least one adjuvant treatments - Full Analysis Set Population 43](#_heading=)

[**5 Efficacy Analyses 44**](#_heading=)

[5.1 Time to event analyses 44](#_heading=)

[Table 5.1.1 Summary of time from herceptin adjuvant treatment to PFS, overall and by pCR result - Kaplan-Meier estimation - Among subjects with herceptin adjuvant treatment start date available - Full Analysis Set Population 44](#_heading=)

[Table 5.1.2 Survival probabilities of time from herceptin adjuvant treatment to PFS, overall and by pCR result - Kaplan-Meier estimation - Among subjects with herceptin adjuvant treatment start date available - Full Analysis Set Population 45](#_heading=)

[Table 5.1.3 Summary of time from herceptin adjuvant treatment to PFS - Kaplan-Meier curve - Among subjects with herceptin adjuvant treatment start date available - Full Analysis Set Population 46](#_heading=)

[Table 5.1.4 Summary of time from herceptin adjuvant treatment to PFS by pCR result - Kaplan-Meier curve - Among subjects with herceptin adjuvant treatment start date available - Full Analysis Set Population 47](#_heading=)

[**6 Exploratory Analyses 48**](#_heading=)

[6.1 Predictive factors for PFS 48](#_heading=)

[Table 6.1.1 PFS - Univariate Cox proportional hazard analysis - Among subjects with herceptin adjuvant treatment start date available - Full Analysis Set Population 48](#_heading=)

[Table 6.1.2 PFS - Multivariate Cox proportional hazard analysis - Among subjects with herceptin adjuvant treatment start date available - Full Analysis Set Population 51](#_heading=)

[6.2 Predictive factors for pCR result 52](#_heading=)

[Table 6.2.1 pCR result - Univariate analysis - Full Analysis Set Population 52](#_heading=)

[Table 6.2.2 pCR result - Multivariate analysis - Full Analysis Set Population 55](#_heading=)

[6.3 Predictive factors for PFS and pCR result 56](#_heading=)

[Table 6.3.1 Correlation matrix - Full Analysis Set Population 56](#_heading=)

[Figure 6.3.2 Correlation coefficient matrix - Full Analysis Set Population 58](#_heading=)

# 1 Analysis of study conduct

## 1.1 Patient Disposition

### Table 1.1.1 Summary of patient disposition - Full Analysis Set Population

| Characteristic | All (N = 315) |
| --- | --- |
| Follow-up duration (years) |  |
| Nobs | 221 |
| Mean (SD) | 4.49 (0.81) |
| Median (Q1;Q3) | 4.6 (4.3; 4.9) |
| Min - Max | 1.0, 5.9 |
| Missing | 94 |
| Is the patient still being followed in the site (as of December 31, 2018)?, n/N (%) |  |
| Yes | 251/305 (82.3%) |
| No | 54/305 (17.7%) |
| Missing | 10 |
| Time from diagnostic to surgery (months) |  |
| Nobs | 246 |
| Mean (SD) | 7.47 (5.68) |
| Median (Q1;Q3) | 7.0 (5.0; 10.0) |
| Min - Max | -3.9, 58.0 |
| Missing | 69 |
| Has there been any progression of the disease since the beginning of adjuvant therapy, n/N (%) |  |
| Yes | 46/301 (15.3%) |
| No | 255/301 (84.7%) |
| Missing | 14 |
| Follow-up duration (years) = (Last consultation date/Death date – initial diagnosis date of breast cancer + 1) / 365.25 | |
| Time from diagnostic to surgery (months) = (Surgery date – initial diagnosis date of breast cancer) / (365.25/12) | |

### Table 1.1.2 Latest news - Among patients not being followed in the site - Full Analysis Set Population

| Characteristic | All (N = 54) |
| --- | --- |
| Latest news |  |
| The patient is dead | 17/52 (32.7%) |
| The patient is lost to follow-up | 31/52 (59.6%) |
| Other reason | 4/52 (7.7%) |
| Missing | 2 |

### Table 1.1.3 Cause of death - Among dead patients - Full Analysis Set Population

| Characteristic | All (N = 17) |
| --- | --- |
| Cause of death |  |
| Disease progression | 10/15 (66.7%) |
| Other reason | 5/15 (33.3%) |
| Missing | 2 |

### Table 1.1.4 Time from diagnostic to progression - Among patients having experienced progression of the disease since the beginning of adjuvant therapy - Full Analysis Set Population

| Characteristic | All (N = 46) |
| --- | --- |
| Time from diagnostic to progression (years) |  |
| Nobs | 36 |
| Mean (SD) | 2.45 (1.03) |
| Median (Q1;Q3) | 2.5 (1.7; 3.3) |
| Min - Max | 0.8, 4.3 |
| Missing | 10 |
| Time from diagnostic to progression (years) = (Date of the first progression of the disease – initial diagnosis date of breast cancer) / 365.25 | |

### Table 1.1.5 Dates available - Full Analysis Set Population

| Characteristic | All (N = 315) |
| --- | --- |
| Birth date available |  |
| Yes | 315/315 (100.0%) |
| No | 0/315 (0.0%) |
| Initial diagnosis date of breast cancer available |  |
| Yes | 254/315 (80.6%) |
| No | 61/315 (19.4%) |
| Surgery date available |  |
| Yes | 296/315 (94.0%) |
| No | 19/315 (6.0%) |
| Date of last consultation available among patients still being followed in the site (as of December 31, 2018) |  |
| Yes | 251/251 (100.0%) |
| No | 0/251 (0.0%) |
| Date of the first progression of the disease available among patients having experienced progression of the disease since the beginning of adjuvant therapy |  |
| Yes | 46/46 (100.0%) |
| No | 0/46 (0.0%) |
| Death date available among dead patients |  |
| Yes | 17/17 (100.0%) |
| No | 0/17 (0.0%) |

# 2 Baseline characteristics

## 2.1 Demographics and baseline disease characteristics

### Table 2.1.1 Summary of demographics and baseline disease characteristics - Full Analysis Set Population

| Characteristic | All (N = 315) |
| --- | --- |
| Age at adjuvant treatment initiation of Herceptin (years) |  |
| Nobs | 303 |
| Mean (SD) | 52.56 (12.63) |
| Median (Q1;Q3) | 52.0 (43.0; 61.0) |
| Min - Max | 24, 94 |
| Missing | 12 |
| Age group (years), n/N (%) |  |
| <40 | 53/303 (17.5%) |
| [40 - 49] | 67/303 (22.1%) |
| [50 - 59] | 94/303 (31.0%) |
| [60 - 69] | 58/303 (19.1%) |
| >=70 | 31/303 (10.2%) |
| Missing | 12 |
| BMI (kg/m2), n/N (%) |  |
| <25 | 185/311 (59.5%) |
| [25 - 30[ | 32/311 (10.3%) |
| >=30 | 94/311 (30.2%) |
| Missing | 4 |
| Professional situation, n/N (%) |  |
| Worker | 150/264 (56.8%) |
| Jobless person | 64/264 (24.2%) |
| Data not found | 50/264 (18.9%) |
| Missing | 51 |
| Weight (kg) |  |
| Nobs | 312 |
| Mean (SD) | 141,395,765.98 (2,497,563,360.54) |
| Median (Q1;Q3) | 67.0 (51.0; 88.0) |
| Min - Max | -228,936, 44,115,763,781 |
| Missing | 3 |
| Height(cm) |  |
| Nobs | 311 |
| Mean (SD) | 9,551.27 (160,077.62) |
| Median (Q1;Q3) | 163.0 (152.0; 189.5) |
| Min - Max | -520,130, 2,695,202 |
| Missing | 4 |
| Weight at initiation of adjuvant therapy (kg) |  |
| Nobs | 263 |
| Mean (SD) | 519,355.57 (8,408,375.42) |
| Median (Q1;Q3) | 65.0 (46.5; 86.5) |
| Min - Max | -18,834, 136,361,615 |
| Missing | 52 |
| Classification T, n/N (%) |  |
| T0 | 1/310 (0.3%) |
| T1 | 24/310 (7.7%) |
| T2 | 161/310 (51.9%) |
| T3 | 75/310 (24.2%) |
| T4 | 46/310 (14.8%) |
| TX | 3/310 (1.0%) |
| Missing | 5 |
| Classification N, n/N (%) |  |
| N0 | 111/307 (36.2%) |
| N1 | 140/307 (45.6%) |
| N2 | 25/307 (8.1%) |
| N3 | 5/307 (1.6%) |
| NX | 26/307 (8.5%) |
| Missing | 8 |
| Histology at the initial diagnosis, n/N (%) |  |
| Invasive ductal carcinoma | 284/306 (92.8%) |
| Invasive lobular carcinoma | 10/306 (3.3%) |
| Mixed carcinoma | 1/306 (0.3%) |
| Other | 9/306 (2.9%) |
| Unknown | 2/306 (0.7%) |
| Missing | 9 |
| Presence of vascular emboli, n/N (%) |  |
| Yes | 25/223 (11.2%) |
| No | 198/223 (88.8%) |
| Missing | 92 |
| SBR grade, n/N (%) |  |
| SBR I | 7/303 (2.3%) |
| SBR II | 139/303 (45.9%) |
| SBR III | 151/303 (49.8%) |
| Ungradable | 6/303 (2.0%) |
| Missing | 12 |
| Number of lymph nodes invaded |  |
| Nobs | 226 |
| Mean (SD) | 1,617.39 (120,669.37) |
| Median (Q1;Q3) | 0.0 (-4.0; 4.0) |
| Min - Max | -903,593, 1,553,394 |
| Missing | 89 |
| Estrogen receptors, n/N (%) |  |
| positive | 176/303 (58.1%) |
| negative | 127/303 (41.9%) |
| not assessable | 0/303 (0.0%) |
| Missing | 12 |
| Progesterone receptors, n/N (%) |  |
| positive | 124/300 (41.3%) |
| negative | 176/300 (58.7%) |
| not assessable | 0/300 (0.0%) |
| Missing | 15 |
| Hormonal receptors status, n/N (%) |  |
| ER and/or PR + | 181/301 (60.1%) |
| ER and PR - | 120/301 (39.9%) |
| Missing | 14 |

### Table 2.1.2 Summary of demographics and baseline disease characteristics by pCR result - Full Analysis Set Population

| Characteristic | pCR (N = 132) | No pCR (N = 183) |
| --- | --- | --- |
| Age at adjuvant treatment initiation of Herceptin (years) |  |  |
| Nobs | 130 | 173 |
| Mean (SD) | 53.13 (12.57) | 52.13 (12.69) |
| Median (Q1;Q3) | 52.0 (44.0; 62.8) | 52.0 (43.0; 60.0) |
| Min - Max | 30, 83 | 24, 94 |
| Missing | 2 | 10 |
| Age group (years), n/N (%) |  |  |
| <40 | 22/130 (16.9%) | 31/173 (17.9%) |
| [40 - 49] | 28/130 (21.5%) | 39/173 (22.5%) |
| [50 - 59] | 41/130 (31.5%) | 53/173 (30.6%) |
| [60 - 69] | 24/130 (18.5%) | 34/173 (19.7%) |
| >=70 | 15/130 (11.5%) | 16/173 (9.2%) |
| Missing | 2 | 10 |
| BMI (kg/m2), n/N (%) |  |  |
| <25 | 82/130 (63.1%) | 103/181 (56.9%) |
| [25 - 30[ | 12/130 (9.2%) | 20/181 (11.0%) |
| >=30 | 36/130 (27.7%) | 58/181 (32.0%) |
| Missing | 2 | 2 |
| Professional situation, n/N (%) |  |  |
| Worker | 62/114 (54.4%) | 88/150 (58.7%) |
| Jobless person | 32/114 (28.1%) | 32/150 (21.3%) |
| Data not found | 20/114 (17.5%) | 30/150 (20.0%) |
| Missing | 18 | 33 |
| Weight (kg) |  |  |
| Nobs | 130 | 182 |
| Mean (SD) | -957.47 (9,895.98) | 242,393,425.58 (3,270,078,041.38) |
| Median (Q1;Q3) | 68.5 (53.2; 92.8) | 65.0 (48.2; 85.0) |
| Min - Max | -100,174, 11,318 | -228,936, 44,115,763,781 |
| Missing | 2 | 1 |
| Height(cm) |  |  |
| Nobs | 130 | 181 |
| Mean (SD) | -2,458.23 (48,544.36) | 18,176.88 (205,581.70) |
| Median (Q1;Q3) | 163.0 (153.2; 208.0) | 162.0 (151.0; 182.0) |
| Min - Max | -520,130, 168,836 | -220,404, 2,695,202 |
| Missing | 2 | 2 |
| Weight at initiation of adjuvant therapy (kg) |  |  |
| Nobs | 112 | 151 |
| Mean (SD) | 1,217,160.30 (12,884,995.62) | 1,778.56 (18,449.06) |
| Median (Q1;Q3) | 63.5 (33.5; 83.2) | 66.0 (51.0; 94.5) |
| Min - Max | -18,834, 136,361,615 | -3,259, 225,016 |
| Missing | 20 | 32 |
| Classification T, n/N (%) |  |  |
| T0 | 1/129 (0.8%) | 0/181 (0.0%) |
| T1 | 10/129 (7.8%) | 14/181 (7.7%) |
| T2 | 62/129 (48.1%) | 99/181 (54.7%) |
| T3 | 36/129 (27.9%) | 39/181 (21.5%) |
| T4 | 18/129 (14.0%) | 28/181 (15.5%) |
| TX | 2/129 (1.6%) | 1/181 (0.6%) |
| Missing | 3 | 2 |
| Classification N, n/N (%) |  |  |
| N0 | 45/126 (35.7%) | 66/181 (36.5%) |
| N1 | 60/126 (47.6%) | 80/181 (44.2%) |
| N2 | 11/126 (8.7%) | 14/181 (7.7%) |
| N3 | 4/126 (3.2%) | 1/181 (0.6%) |
| NX | 6/126 (4.8%) | 20/181 (11.0%) |
| Missing | 6 | 2 |
| Histology at the initial diagnosis, n/N (%) |  |  |
| Invasive ductal carcinoma | 117/130 (90.0%) | 167/176 (94.9%) |
| Invasive lobular carcinoma | 3/130 (2.3%) | 7/176 (4.0%) |
| Mixed carcinoma | 1/130 (0.8%) | 0/176 (0.0%) |
| Other | 8/130 (6.2%) | 1/176 (0.6%) |
| Unknown | 1/130 (0.8%) | 1/176 (0.6%) |
| Missing | 2 | 7 |
| Presence of vascular emboli, n/N (%) |  |  |
| Yes | 8/100 (8.0%) | 17/123 (13.8%) |
| No | 92/100 (92.0%) | 106/123 (86.2%) |
| Missing | 32 | 60 |
| SBR grade, n/N (%) |  |  |
| SBR I | 1/129 (0.8%) | 6/174 (3.4%) |
| SBR II | 61/129 (47.3%) | 78/174 (44.8%) |
| SBR III | 65/129 (50.4%) | 86/174 (49.4%) |
| Ungradable | 2/129 (1.6%) | 4/174 (2.3%) |
| Missing | 3 | 9 |
| Number of lymph nodes invaded |  |  |
| Nobs | 99 | 127 |
| Mean (SD) | -39.62 (664.64) | 2,909.07 (161,238.26) |
| Median (Q1;Q3) | 0.0 (-4.0; 4.5) | 0.0 (-3.5; 4.0) |
| Min - Max | -5,184, 2,481 | -903,593, 1,553,394 |
| Missing | 33 | 56 |
| Estrogen receptors, n/N (%) |  |  |
| positive | 64/128 (50.0%) | 112/175 (64.0%) |
| negative | 64/128 (50.0%) | 63/175 (36.0%) |
| not assessable | 0/128 (0.0%) | 0/175 (0.0%) |
| Missing | 4 | 8 |
| Progesterone receptors, n/N (%) |  |  |
| positive | 45/127 (35.4%) | 79/173 (45.7%) |
| negative | 82/127 (64.6%) | 94/173 (54.3%) |
| not assessable | 0/127 (0.0%) | 0/173 (0.0%) |
| Missing | 5 | 10 |
| Hormonal receptors status, n/N (%) |  |  |
| ER and/or PR + | 67/127 (52.8%) | 114/174 (65.5%) |
| ER and PR - | 60/127 (47.2%) | 60/174 (34.5%) |
| Missing | 5 | 9 |

# 3 Surgery and pCR

## 3.1 Surgery

### 3.1.1 Summary of surgery - Among patients with at least one surgery - Full Analysis Set Population

| Characteristic | All (N = 313) |
| --- | --- |
| At least one Surgery* |  |
| Axillary curage | 249 (79.6%) |
| Mastectomy | 166 (53%) |
| Conservative surgery | 146 (46.6%) |
| Sentinel Ganglion | 53 (16.9%) |
| * One patient can have reported several surgery types | |

## 3.2 pCR

### 3.2.1 Summary of pCR - Full Analysis Set Population

| Characteristic | All (N = 315) |
| --- | --- |
| pCR results* |  |
| pCR | 132/315 (41.9%) |
| No pCR | 183/315 (58.1%) |
| Missing | 0 |
| Absence of invasive and in situ residues in the breast and in the lymph nodes |  |
| Yes | 105/254 (41.3%) |
| No | 149/254 (58.7%) |
| Missing | 61 |
| Absence of invasive residues in the breast and lymph nodes, regardless of the presence of ductal carcinoma in situ |  |
| Yes | 112/254 (44.1%) |
| No | 142/254 (55.9%) |
| Missing | 61 |
| Classification Chevallier |  |
| Grade 1 | 1/7 (14.3%) |
| Grade 2 | 2/7 (28.6%) |
| Grade 3 | 4/7 (57.1%) |
| Missing | 308 |
| Classification Sataloff T |  |
| TA | 32/52 (61.5%) |
| TB | 16/52 (30.8%) |
| TC | 4/52 (7.7%) |
| Missing | 263 |
| Classification Sataloff N |  |
| NA | 21/52 (40.4%) |
| NB | 20/52 (38.5%) |
| NC | 8/52 (15.4%) |
| ND | 3/52 (5.8%) |
| Missing | 263 |
| Classification RCB |  |
| RCB-I | 1/2 (50.0%) |
| RCB-II | 1/2 (50.0%) |
| Missing | 313 |
| * pCR results = pCR if ypT0/Tis ypN0 is ticked Yes OR, Grade 1 or Grade 2 are ticked for Classification Chevallier OR, TA or NA are ticked for Classification Sataloff OR, RCB0 is ticked for Classification RCB | |

# 4 Adjuvant treatments

## Table 4.1 Summary of adjuvant treatments - Among subjects with at least one adjuvant treatments - Full Analysis Set Population

| Characteristic | All (N = 305) |
| --- | --- |
| At least one Adjuvant Treatment* |  |
| Trastuzumab (Herceptin) | 305 (100%) |
| Tamoxifene | 81 (26.6%) |
| Letrozole | 40 (13.1%) |
| Anastrozole | 21 (6.9%) |
| Exemestane | 5 (1.6%) |
| Other hormonotherapy 1 | 4 (1.3%) |
| Other | 3 (1%) |
| Carboplatine | 1 (0.3%) |
| Docetaxel | 1 (0.3%) |
| Epirubicine | 1 (0.3%) |
| Paclitaxel | 1 (0.3%) |
| * One patient can have reported several adjuvant treatment types | |

## Table 4.2 Summary of adjuvant treatments by adjuvant treatment - Among subjects with at least one adjuvant treatments - Full Analysis Set Population

| Characteristic | All |
| --- | --- |
| Anastrozole :  - Duration (months) |  |
| Nobs | 0 |
| Mean (SD) | NA (NA) |
| Median (Q1;Q3) | NA (NA; NA) |
| Min - Max | NA, NA |
| Missing | 21 |
| - Number of cycles completed |  |
| Nobs | 0 |
| Mean (SD) | NA (NA) |
| Median (Q1;Q3) | NA (NA; NA) |
| Min - Max | NA, NA |
| Missing | 21 |
| - Start date of treatment available, n/N (%) |  |
| Yes | 18/21 (85.7%) |
| No | 3/21 (14.3%) |
| - End date of treatment available, n/N (%) |  |
| No | 4/4 (100.0%) |
| Carboplatine :  - Duration (months) |  |
| Nobs | 1 |
| Mean (SD) | 0.03 (NA) |
| Median (Q1;Q3) | 0.0 (0.0; 0.0) |
| Min - Max | 0.0, 0.0 |
| Missing | 0 |
| - Number of cycles completed |  |
| Nobs | 1 |
| Mean (SD) | -3.00 (NA) |
| Median (Q1;Q3) | -3.0 (-3.0; -3.0) |
| Min - Max | -3.0, -3.0 |
| Missing | 0 |
| - Start date of treatment available, n/N (%) |  |
| Yes | 1/1 (100.0%) |
| No | 0/1 (0.0%) |
| - End date of treatment available, n/N (%) |  |
| Yes | 1/1 (100.0%) |
| Docetaxel :  - Duration (months) |  |
| Nobs | 1 |
| Mean (SD) | 0.03 (NA) |
| Median (Q1;Q3) | 0.0 (0.0; 0.0) |
| Min - Max | 0.0, 0.0 |
| Missing | 0 |
| - Number of cycles completed |  |
| Nobs | 1 |
| Mean (SD) | -18,532.00 (NA) |
| Median (Q1;Q3) | -18,532.0 (-18,532.0; -18,532.0) |
| Min - Max | -18,532.0, -18,532.0 |
| Missing | 0 |
| - Start date of treatment available, n/N (%) |  |
| Yes | 1/1 (100.0%) |
| No | 0/1 (0.0%) |
| - End date of treatment available, n/N (%) |  |
| Yes | 1/1 (100.0%) |
| Epirubicine :  - Duration (months) |  |
| Nobs | 1 |
| Mean (SD) | 3.06 (NA) |
| Median (Q1;Q3) | 3.1 (3.1; 3.1) |
| Min - Max | 3.1, 3.1 |
| Missing | 0 |
| - Number of cycles completed |  |
| Nobs | 1 |
| Mean (SD) | 6.00 (NA) |
| Median (Q1;Q3) | 6.0 (6.0; 6.0) |
| Min - Max | 6.0, 6.0 |
| Missing | 0 |
| - Start date of treatment available, n/N (%) |  |
| Yes | 1/1 (100.0%) |
| No | 0/1 (0.0%) |
| - End date of treatment available, n/N (%) |  |
| Yes | 1/1 (100.0%) |
| Exemestane :  - Duration (months) |  |
| Nobs | 2 |
| Mean (SD) | 10.05 (9.90) |
| Median (Q1;Q3) | 10.1 (6.6; 13.6) |
| Min - Max | 3.1, 17.1 |
| Missing | 3 |
| - Number of cycles completed |  |
| Nobs | 0 |
| Mean (SD) | NA (NA) |
| Median (Q1;Q3) | NA (NA; NA) |
| Min - Max | NA, NA |
| Missing | 5 |
| - Start date of treatment available, n/N (%) |  |
| Yes | 4/5 (80.0%) |
| No | 1/5 (20.0%) |
| - End date of treatment available, n/N (%) |  |
| No | 1/3 (33.3%) |
| Yes | 2/3 (66.7%) |
| Letrozole :  - Duration (months) |  |
| Nobs | 10 |
| Mean (SD) | 15.75 (12.42) |
| Median (Q1;Q3) | 12.5 (8.6; 16.1) |
| Min - Max | 3.0, 43.1 |
| Missing | 30 |
| - Number of cycles completed |  |
| Nobs | 0 |
| Mean (SD) | NA (NA) |
| Median (Q1;Q3) | NA (NA; NA) |
| Min - Max | NA, NA |
| Missing | 40 |
| - Start date of treatment available, n/N (%) |  |
| Yes | 39/40 (97.5%) |
| No | 1/40 (2.5%) |
| - End date of treatment available, n/N (%) |  |
| No | 8/18 (44.4%) |
| Yes | 10/18 (55.6%) |
| Other :  - Duration (months) |  |
| Nobs | 1 |
| Mean (SD) | 4.04 (NA) |
| Median (Q1;Q3) | 4.0 (4.0; 4.0) |
| Min - Max | 4.0, 4.0 |
| Missing | 2 |
| - Number of cycles completed |  |
| Nobs | 2 |
| Mean (SD) | 357.00 (511.95) |
| Median (Q1;Q3) | 357.0 (176.0; 538.0) |
| Min - Max | -5.0, 719.0 |
| Missing | 1 |
| - Start date of treatment available, n/N (%) |  |
| Yes | 2/3 (66.7%) |
| No | 1/3 (33.3%) |
| - End date of treatment available, n/N (%) |  |
| No | 2/3 (66.7%) |
| Yes | 1/3 (33.3%) |
| Other hormonotherapy 1 :  - Duration (months) |  |
| Nobs | 2 |
| Mean (SD) | 30.54 (13.45) |
| Median (Q1;Q3) | 30.5 (25.8; 35.3) |
| Min - Max | 21.0, 40.0 |
| Missing | 2 |
| - Number of cycles completed |  |
| Nobs | 0 |
| Mean (SD) | NA (NA) |
| Median (Q1;Q3) | NA (NA; NA) |
| Min - Max | NA, NA |
| Missing | 4 |
| - Start date of treatment available, n/N (%) |  |
| Yes | 3/4 (75.0%) |
| No | 1/4 (25.0%) |
| - End date of treatment available, n/N (%) |  |
| No | 1/3 (33.3%) |
| Yes | 2/3 (66.7%) |
| Paclitaxel :  - Duration (months) |  |
| Nobs | 0 |
| Mean (SD) | NA (NA) |
| Median (Q1;Q3) | NA (NA; NA) |
| Min - Max | NA, NA |
| Missing | 1 |
| - Number of cycles completed |  |
| Nobs | 0 |
| Mean (SD) | NA (NA) |
| Median (Q1;Q3) | NA (NA; NA) |
| Min - Max | NA, NA |
| Missing | 1 |
| - Start date of treatment available, n/N (%) |  |
| Yes | 0/1 (0.0%) |
| No | 1/1 (100.0%) |
| - End date of treatment available, n/N (%) |  |
| No | 1/1 (100.0%) |
| Tamoxifene :  - Duration (months) |  |
| Nobs | 23 |
| Mean (SD) | 21.52 (11.91) |
| Median (Q1;Q3) | 22.0 (10.1; 33.1) |
| Min - Max | 3.0, 43.1 |
| Missing | 58 |
| - Number of cycles completed |  |
| Nobs | 0 |
| Mean (SD) | NA (NA) |
| Median (Q1;Q3) | NA (NA; NA) |
| Min - Max | NA, NA |
| Missing | 81 |
| - Start date of treatment available, n/N (%) |  |
| Yes | 74/81 (91.4%) |
| No | 7/81 (8.6%) |
| - End date of treatment available, n/N (%) |  |
| No | 19/42 (45.2%) |
| Yes | 23/42 (54.8%) |
| Trastuzumab (Herceptin) :  - Duration (months) |  |
| Nobs | 294 |
| Mean (SD) | 8.23 (2.25) |
| Median (Q1;Q3) | 9.0 (7.1; 9.1) |
| Min - Max | 0.0, 20.0 |
| Missing | 11 |
| - Number of cycles completed |  |
| Nobs | 298 |
| Mean (SD) | 1,517.43 (39,009.55) |
| Median (Q1;Q3) | 12.0 (5.0; 16.0) |
| Min - Max | -349,221.0, 475,959.0 |
| Missing | 7 |
| - Start date of treatment available, n/N (%) |  |
| Yes | 303/305 (99.3%) |
| No | 2/305 (0.7%) |
| - End date of treatment available, n/N (%) |  |
| No | 10/305 (3.3%) |
| Yes | 295/305 (96.7%) |
| Duration of each adjuvant (months) = (End date of treatment – Start date of treatment + 1) / (365.25/12) | |

## Table 4.3 Time between surgery and adjuvant treatment - Among subjects with at least one adjuvant treatments - Full Analysis Set Population

| Characteristic | All (N = 305) |
| --- | --- |
| Time from surgery to adjuvant treatment initiation of Herceptin (days) |  |
| Nobs | 293 |
| Mean (SD) | 8.40 (106.93) |
| Median (Q1;Q3) | 0.0 (-28.0; 31.0) |
| Min - Max | -426.0, 424.0 |
| Missing | 12 |
| Time from surgery to adjuvant treatment initiation of Herceptin (days) = (Date of adjuvant treatment initiation of Herceptin - Surgery date) | |

## Table 4.4 Summary of adjuvant treatments by pCR status - Among subjects with at least one adjuvant treatments - Full Analysis Set Population

| Characteristic | pCR (N = 130) | No pCR (N = 175) |
| --- | --- | --- |
| At least one Adjuvant Treatment* |  |  |
| Trastuzumab (Herceptin) | 130 (100%) | 175 (100%) |
| Tamoxifene | 31 (23.8%) | 50 (28.6%) |
| Letrozole | 13 (10%) | 27 (15.4%) |
| Anastrozole | 7 (5.4%) | 14 (8%) |
| Exemestane | 1 (0.8%) | 4 (2.3%) |
| Other hormonotherapy 1 | 0 (0%) | 4 (2.3%) |
| Other | 1 (0.8%) | 2 (1.1%) |
| Carboplatine | 1 (0.8%) | 0 (0%) |
| Docetaxel | 1 (0.8%) | 0 (0%) |
| Epirubicine | 0 (0%) | 1 (0.6%) |
| Paclitaxel | 1 (0.8%) | 0 (0%) |

## Table 4.5 Summary of adjuvant treatments by adjuvant treatment by pCR status - Among subjects with at least one adjuvant treatments - Full Analysis Set Population

| Characteristic | pCR (N = 130) | No pCR (N = 175) |
| --- | --- | --- |
| Anastrozole :  - Duration (months) |  |  |
| Nobs | 0 | 0 |
| Mean (SD) | NA (NA) | NA (NA) |
| Median (Q1;Q3) | NA (NA; NA) | NA (NA; NA) |
| Min - Max | NA, NA | NA, NA |
| Missing | 7 | 14 |
| - Number of cycles completed |  |  |
| Nobs | 0 | 0 |
| Mean (SD) | NA (NA) | NA (NA) |
| Median (Q1;Q3) | NA (NA; NA) | NA (NA; NA) |
| Min - Max | NA, NA | NA, NA |
| Missing | 7 | 14 |
| Carboplatine :  - Duration (months) |  |  |
| Nobs | 1 | 0 |
| Mean (SD) | 0.03 (NA) | NA (NA) |
| Median (Q1;Q3) | 0.0 (0.0; 0.0) | NA (NA; NA) |
| Min - Max | 0.0, 0.0 | NA, NA |
| Missing | 0 | 0 |
| - Number of cycles completed |  |  |
| Nobs | 1 | 0 |
| Mean (SD) | -3.00 (NA) | NA (NA) |
| Median (Q1;Q3) | -3.0 (-3.0; -3.0) | NA (NA; NA) |
| Min - Max | -3.0, -3.0 | NA, NA |
| Missing | 0 | 0 |
| Docetaxel :  - Duration (months) |  |  |
| Nobs | 1 | 0 |
| Mean (SD) | 0.03 (NA) | NA (NA) |
| Median (Q1;Q3) | 0.0 (0.0; 0.0) | NA (NA; NA) |
| Min - Max | 0.0, 0.0 | NA, NA |
| Missing | 0 | 0 |
| - Number of cycles completed |  |  |
| Nobs | 1 | 0 |
| Mean (SD) | -18,532.00 (NA) | NA (NA) |
| Median (Q1;Q3) | -18,532.0 (-18,532.0; -18,532.0) | NA (NA; NA) |
| Min - Max | -18,532.0, -18,532.0 | NA, NA |
| Missing | 0 | 0 |
| Epirubicine :  - Duration (months) |  |  |
| Nobs | 0 | 1 |
| Mean (SD) | NA (NA) | 3.06 (NA) |
| Median (Q1;Q3) | NA (NA; NA) | 3.1 (3.1; 3.1) |
| Min - Max | NA, NA | 3.1, 3.1 |
| Missing | 0 | 0 |
| - Number of cycles completed |  |  |
| Nobs | 0 | 1 |
| Mean (SD) | NA (NA) | 6.00 (NA) |
| Median (Q1;Q3) | NA (NA; NA) | 6.0 (6.0; 6.0) |
| Min - Max | NA, NA | 6.0, 6.0 |
| Missing | 0 | 0 |
| Exemestane :  - Duration (months) |  |  |
| Nobs | 0 | 2 |
| Mean (SD) | NA (NA) | 10.05 (9.90) |
| Median (Q1;Q3) | NA (NA; NA) | 10.1 (6.6; 13.6) |
| Min - Max | NA, NA | 3.1, 17.1 |
| Missing | 1 | 2 |
| - Number of cycles completed |  |  |
| Nobs | 0 | 0 |
| Mean (SD) | NA (NA) | NA (NA) |
| Median (Q1;Q3) | NA (NA; NA) | NA (NA; NA) |
| Min - Max | NA, NA | NA, NA |
| Missing | 1 | 4 |
| Letrozole :  - Duration (months) |  |  |
| Nobs | 4 | 6 |
| Mean (SD) | 18.79 (16.52) | 13.72 (10.09) |
| Median (Q1;Q3) | 13.0 (11.3; 20.6) | 11.1 (8.6; 15.8) |
| Min - Max | 6.0, 43.1 | 3.0, 32.0 |
| Missing | 9 | 21 |
| - Number of cycles completed |  |  |
| Nobs | 0 | 0 |
| Mean (SD) | NA (NA) | NA (NA) |
| Median (Q1;Q3) | NA (NA; NA) | NA (NA; NA) |
| Min - Max | NA, NA | NA, NA |
| Missing | 13 | 27 |
| Other :  - Duration (months) |  |  |
| Nobs | 0 | 1 |
| Mean (SD) | NA (NA) | 4.04 (NA) |
| Median (Q1;Q3) | NA (NA; NA) | 4.0 (4.0; 4.0) |
| Min - Max | NA, NA | 4.0, 4.0 |
| Missing | 1 | 1 |
| - Number of cycles completed |  |  |
| Nobs | 1 | 1 |
| Mean (SD) | -5.00 (NA) | 719.00 (NA) |
| Median (Q1;Q3) | -5.0 (-5.0; -5.0) | 719.0 (719.0; 719.0) |
| Min - Max | -5.0, -5.0 | 719.0, 719.0 |
| Missing | 0 | 1 |
| Other hormonotherapy 1 :  - Duration (months) |  |  |
| Nobs | 0 | 2 |
| Mean (SD) | NA (NA) | 30.54 (13.45) |
| Median (Q1;Q3) | NA (NA; NA) | 30.5 (25.8; 35.3) |
| Min - Max | NA, NA | 21.0, 40.0 |
| Missing | 0 | 2 |
| - Number of cycles completed |  |  |
| Nobs | 0 | 0 |
| Mean (SD) | NA (NA) | NA (NA) |
| Median (Q1;Q3) | NA (NA; NA) | NA (NA; NA) |
| Min - Max | NA, NA | NA, NA |
| Missing | 0 | 4 |
| Paclitaxel :  - Duration (months) |  |  |
| Nobs | 0 | 0 |
| Mean (SD) | NA (NA) | NA (NA) |
| Median (Q1;Q3) | NA (NA; NA) | NA (NA; NA) |
| Min - Max | NA, NA | NA, NA |
| Missing | 1 | 0 |
| - Number of cycles completed |  |  |
| Nobs | 0 | 0 |
| Mean (SD) | NA (NA) | NA (NA) |
| Median (Q1;Q3) | NA (NA; NA) | NA (NA; NA) |
| Min - Max | NA, NA | NA, NA |
| Missing | 1 | 0 |
| Tamoxifene :  - Duration (months) |  |  |
| Nobs | 11 | 12 |
| Mean (SD) | 24.86 (10.19) | 18.46 (12.95) |
| Median (Q1;Q3) | 24.0 (18.6; 35.0) | 13.0 (9.8; 25.8) |
| Min - Max | 9.0, 38.0 | 3.0, 43.1 |
| Missing | 20 | 38 |
| - Number of cycles completed |  |  |
| Nobs | 0 | 0 |
| Mean (SD) | NA (NA) | NA (NA) |
| Median (Q1;Q3) | NA (NA; NA) | NA (NA; NA) |
| Min - Max | NA, NA | NA, NA |
| Missing | 31 | 50 |
| Trastuzumab (Herceptin) :  - Duration (months) |  |  |
| Nobs | 126 | 168 |
| Mean (SD) | 8.06 (2.32) | 8.36 (2.20) |
| Median (Q1;Q3) | 9.0 (7.0; 9.1) | 9.0 (8.0; 9.1) |
| Min - Max | 0.0, 20.0 | 0.0, 14.1 |
| Missing | 4 | 7 |
| - Number of cycles completed |  |  |
| Nobs | 127 | 171 |
| Mean (SD) | 2,570.76 (28,606.08) | 735.13 (45,283.81) |
| Median (Q1;Q3) | 12.0 (6.0; 15.0) | 12.0 (4.0; 17.0) |
| Min - Max | -7,187.0, 322,131.0 | -349,221.0, 475,959.0 |
| Missing | 3 | 4 |
| Duration of each adjuvant (months) = (End date of treatment – Start date of treatment + 1) / (365.25/12) | | |

## Table 4.6 Time between surgery and adjuvant treatment by pCR status - Among subjects with at least one adjuvant treatments - Full Analysis Set Population

| Characteristic | pCR (N = 130) | No pCR (N = 175) |
| --- | --- | --- |
| Time from surgery to adjuvant treatment initiation of Herceptin (days) |  |  |
| Nobs | 126 | 167 |
| Mean (SD) | 3.61 (121.24) | 12.01 (94.95) |
| Median (Q1;Q3) | 0.0 (-29.5; 31.0) | 0.0 (0.0; 31.0) |
| Min - Max | -426.0, 424.0 | -274.0, 334.0 |
| Missing | 4 | 8 |
| Time from surgery to adjuvant treatment initiation of Herceptin (days) = (Date of adjuvant treatment initiation of Herceptin - Surgery date) | | |

# 5 Efficacy Analyses

## 5.1 Time to event analyses

### Table 5.1.1 Summary of time from herceptin adjuvant treatment to PFS, overall and by pCR result - Kaplan-Meier estimation - Among subjects with herceptin adjuvant treatment start date available - Full Analysis Set Population

| PFS | N | Number of event | 10% Percentile (95% CI) |
| --- | --- | --- | --- |
| Overall | 303 | 51 | 1.9 (1.5, 2.6) |
| pCR results* |  |  |  |
| pCR | 130 | 17 | 2.3 (1.3, —) |
| No pCR | 173 | 34 | 1.8 (1.2, 2.4) |
| * pCR results = pCR if ypT0/Tis ypN0 is ticked Yes OR, Grade 1 or Grade 2 are ticked for Classification Chevallier OR, TA and NA are ticked for Classification Sataloff OR, RCB0 is ticked for Classification RCB | | | |
| Patients who did not experience event were censored at their last consultation date. If this date is missing, they were censored at the last adjuvant treatment date | | | |

### Table 5.1.2 Survival probabilities of time from herceptin adjuvant treatment to PFS, overall and by pCR result - Kaplan-Meier estimation - Among subjects with herceptin adjuvant treatment start date available - Full Analysis Set Population

| PFS | N | At 1 year (95% CI) | At 2 years (95% CI) | At 3 years (95% CI) | At 4 years (95% CI) |
| --- | --- | --- | --- | --- | --- |
| Overall | 303 | 96.2 (93.3, 97.9) | 89.9 (85.7, 92.9) | 84.3 (79.4, 88.1) | 81.1 (75.7, 85.4) |
| pCR results* |  |  |  |  |  |
| pCR | 130 | 95.9 (90.5, 98.3) | 91.7 (85.1, 95.4) | 88.2 (80.9, 92.8) | 84.7 (76.2, 90.3) |
| No pCR | 173 | 96.5 (92.3, 98.4) | 88.5 (82.4, 92.6) | 81.3 (74.2, 86.6) | 78.3 (70.8, 84.1) |
| * pCR results = pCR if ypT0/Tis ypN0 is ticked Yes OR, Grade 1 or Grade 2 are ticked for Classification Chevallier OR, TA and NA are ticked for Classification Sataloff OR, RCB0 is ticked for Classification RCB | | | | | |
| Patients who did not experience event were censored at their last consultation date. If this date is missing, they were censored at the last adjuvant treatment date | | | | | |

### Table 5.1.3 Summary of time from herceptin adjuvant treatment to PFS - Kaplan-Meier curve - Among subjects with herceptin adjuvant treatment start date available - Full Analysis Set Population


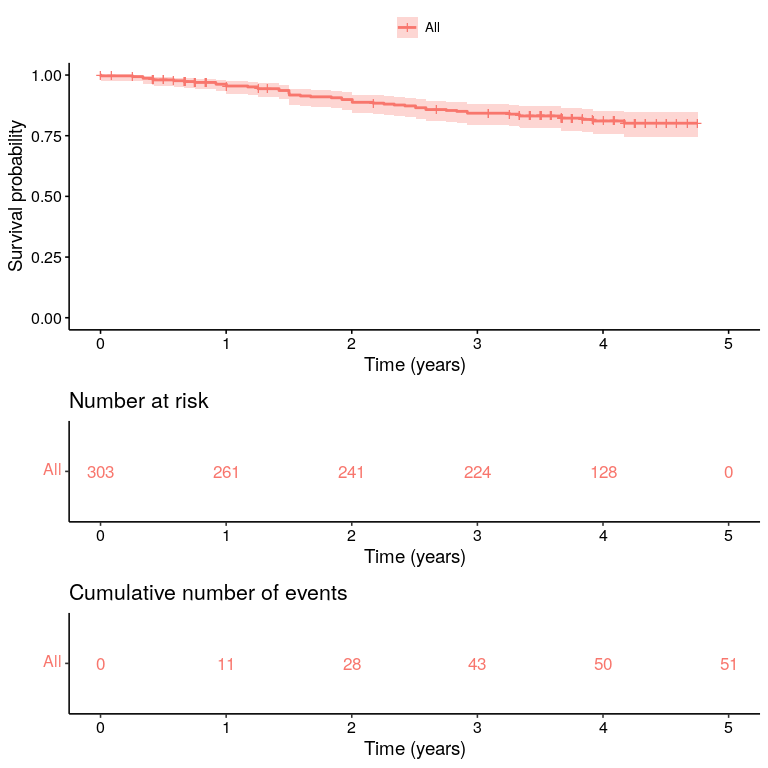


### Table 5.1.4 Summary of time from herceptin adjuvant treatment to PFS by pCR result - Kaplan-Meier curve - Among subjects with herceptin adjuvant treatment start date available - Full Analysis Set Population


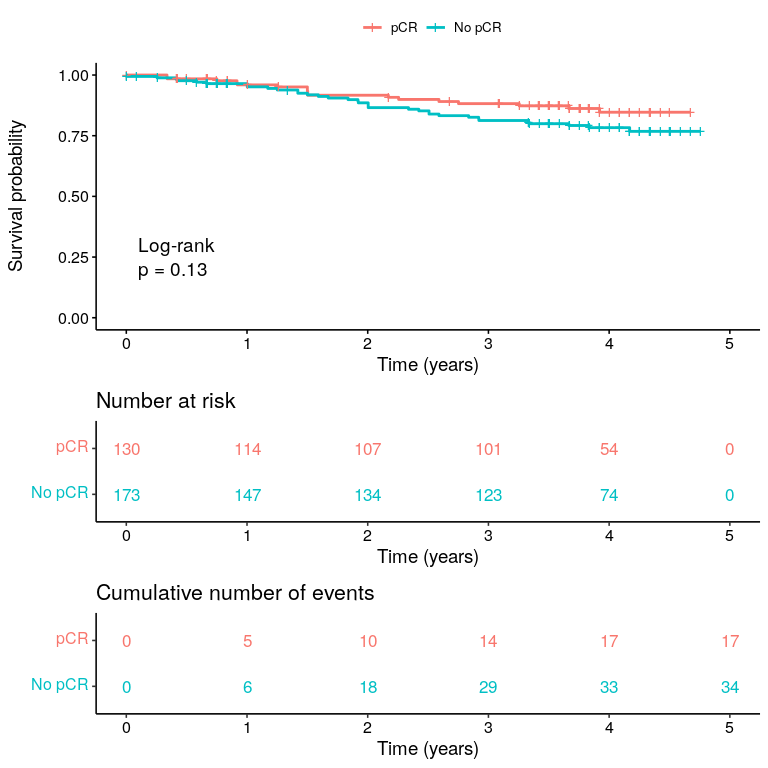


# 6 Exploratory Analyses

## 6.1 Predictive factors for PFS

### Table 6.1.1 PFS - Univariate Cox proportional hazard analysis - Among subjects with herceptin adjuvant treatment start date available - Full Analysis Set Population

|  | Descriptive statistics | | HR and 95% CI | | |
| --- | --- | --- | --- | --- | --- |
| Characteristic | Event, N = 51 | No event, N = 252 | HR^1^ | 95% CI^1^ | p-value |
| Age at adjuvant treatment initiation of Herceptin (years) |  |  | 0.99 | 0.97, 1.01 | 0.512 |
| N | 51 | 252 |  |  |  |
| Mean (SD) | 51.43 (14.27) | 52.79 (12.29) |  |  |  |
| Median (25%; 75%) | 51.0 (44.0; 58.0) | 52.0 (43.0; 61.0) |  |  |  |
| Range | 24, 94 | 30, 83 |  |  |  |
| Missing | 0 | 0 |  |  |  |
| Age group (years) |  |  |  |  | 0.357 |
| <40 | 12/53 (22.6%) | 41/53 (77.4%) | — | — |  |
| [40 - 49] | 7/67 (10.4%) | 60/67 (89.6%) | 0.46 | 0.18, 1.17 |  |
| [50 - 59] | 20/94 (21.3%) | 74/94 (78.7%) | 0.94 | 0.46, 1.92 |  |
| [60 - 69] | 8/58 (13.8%) | 50/58 (86.2%) | 0.60 | 0.24, 1.46 |  |
| >=70 | 4/31 (12.9%) | 27/31 (87.1%) | 0.56 | 0.18, 1.72 |  |
| Missing | 0 | 0 |  |  |  |
| BMI (kg/m2) |  |  |  |  | 0.894 |
| <25 | 30/178 (16.9%) | 148/178 (83.1%) | — | — |  |
| [25 - 30[ | 6/31 (19.4%) | 25/31 (80.6%) | 1.19 | 0.49, 2.86 |  |
| >=30 | 14/90 (15.6%) | 76/90 (84.4%) | 0.94 | 0.50, 1.78 |  |
| Missing | 1 | 3 |  |  |  |
| T classification |  |  |  |  | 0.048 |
| T0-3 | 38/252 (15.1%) | 214/252 (84.9%) | — | — |  |
| T>3 | 12/43 (27.9%) | 31/43 (72.1%) | 1.92 | 1.00, 3.68 |  |
| Missing | 1 | 7 |  |  |  |
| N classification |  |  |  |  | 0.243 |
| N0 | 14/106 (13.2%) | 92/106 (86.8%) | — | — |  |
| N1 | 28/136 (20.6%) | 108/136 (79.4%) | 1.61 | 0.85, 3.07 |  |
| N2&N3 | 7/29 (24.1%) | 22/29 (75.9%) | 1.93 | 0.78, 4.78 |  |
| Missing | 2 | 30 |  |  |  |
| SBR Grade |  |  |  |  | 0.513 |
| SBR I & II | 22/143 (15.4%) | 121/143 (84.6%) | — | — |  |
| SBR III | 28/151 (18.5%) | 123/151 (81.5%) | 1.21 | 0.69, 2.11 |  |
| Missing | 1 | 8 |  |  |  |
| Presence of vascular emboli |  |  |  |  | 0.002 |
| Yes | 8/25 (32.0%) | 17/25 (68.0%) | — | — |  |
| No | 27/197 (13.7%) | 170/197 (86.3%) | 0.28 | 0.13, 0.62 |  |
| Missing | 16 | 65 |  |  |  |
| Hormonal receptors status |  |  |  |  | 0.371 |
| ER and/or PR + | 27/179 (15.1%) | 152/179 (84.9%) | — | — |  |
| ER and PR - | 23/119 (19.3%) | 96/119 (80.7%) | 1.29 | 0.74, 2.25 |  |
| Missing | 1 | 4 |  |  |  |
| pCR results* |  |  |  |  | 0.132 |
| pCR | 17/130 (13.1%) | 113/130 (86.9%) | — | — |  |
| No pCR | 34/173 (19.7%) | 139/173 (80.3%) | 1.56 | 0.87, 2.80 |  |
| Missing | 0 | 0 |  |  |  |
| * pCR results = pCR if ypT0/Tis ypN0 is ticked Yes OR, Grade 1 or Grade 2 are ticked for Classification Chevallier OR, TA and NA are ticked for Classification Sataloff OR, RCB0 is ticked for Classification RCB | | | | | |
| Univariate analysis has been done using a cox model. P-value is based on a global wald test from Cox model | | | | | |
| ^1^HR = Hazard Ratio, CI = Confidence Interval | | | | | |

### Table 6.1.2 PFS - Multivariate Cox proportional hazard analysis - Among subjects with herceptin adjuvant treatment start date available - Full Analysis Set Population

|  | Descriptive statistics | | HR and 95% CI | | |
| --- | --- | --- | --- | --- | --- |
| Characteristic | Event, N = 51 | No event, N = 252 | HR^1^ | 95% CI^1^ | p-value |
| Presence of vascular emboli |  |  |  |  | 0.004 |
| Yes | 8/25 (32.0%) | 17/25 (68.0%) | — | — |  |
| No | 27/197 (13.7%) | 170/197 (86.3%) | 0.31 | 0.14, 0.68 |  |
| Missing | 16 | 65 |  |  |  |
| pCR results* |  |  |  |  | 0.102 |
| pCR | 17/130 (13.1%) | 113/130 (86.9%) | — | — |  |
| No pCR | 34/173 (19.7%) | 139/173 (80.3%) | 1.82 | 0.89, 3.74 |  |
| Missing | 0 | 0 |  |  |  |
| * pCR results = pCR if ypT0/Tis ypN0 is ticked Yes OR, Grade 1 or Grade 2 are ticked for Classification Chevallier OR, TA and NA are ticked for Classification Sataloff OR, RCB0 is ticked for Classification RCB | | | | | |
| Multivariate analysis has been done using a cox model. P-value is based on a global wald test from Cox model. The model has been constructed using a stepwise selection of covariates with 0.15 as entry threshold and 0.15 as the retention threshold. | | | | | |
| ^1^HR = Hazard Ratio, CI = Confidence Interval | | | | | |

## 6.2 Predictive factors for pCR result

### Table 6.2.1 pCR result - Univariate analysis - Full Analysis Set Population

|  | Descriptive statistics | | OR and 95% CI | | |
| --- | --- | --- | --- | --- | --- |
| Characteristic | pCR, N = 132 | No pCR, N = 183 | OR^1^ | 95% CI^1^ | p-value |
| Age at adjuvant treatment initiation of Herceptin (years) |  |  | 1.01 | 0.99, 1.02 | 0.493 |
| N | 130 | 173 |  |  |  |
| Mean (SD) | 53.13 (12.57) | 52.13 (12.69) |  |  |  |
| Median (25%; 75%) | 52.0 (44.0; 62.8) | 52.0 (43.0; 60.0) |  |  |  |
| Range | 30, 83 | 24, 94 |  |  |  |
| Missing | 2 | 10 |  |  |  |
| Age group (years) |  |  |  |  | 0.975 |
| <40 | 22/53 (41.5%) | 31/53 (58.5%) | — | — |  |
| [40 - 49] | 28/67 (41.8%) | 39/67 (58.2%) | 1.01 | 0.49, 2.11 |  |
| [50 - 59] | 41/94 (43.6%) | 53/94 (56.4%) | 1.09 | 0.55, 2.17 |  |
| [60 - 69] | 24/58 (41.4%) | 34/58 (58.6%) | 0.99 | 0.47, 2.13 |  |
| >=70 | 15/31 (48.4%) | 16/31 (51.6%) | 1.32 | 0.54, 3.24 |  |
| Missing | 2 | 10 |  |  |  |
| BMI (kg/m2) |  |  |  |  | 0.473 |
| <25 | 82/185 (44.3%) | 103/185 (55.7%) | — | — |  |
| [25 - 30[ | 12/32 (37.5%) | 20/32 (62.5%) | 0.75 | 0.34, 1.61 |  |
| >=30 | 36/94 (38.3%) | 58/94 (61.7%) | 0.78 | 0.47, 1.29 |  |
| Missing | 2 | 2 |  |  |  |
| T classification |  |  |  |  | 0.738 |
| T0-3 | 109/261 (41.8%) | 152/261 (58.2%) | — | — |  |
| T>3 | 18/46 (39.1%) | 28/46 (60.9%) | 0.90 | 0.47, 1.69 |  |
| Missing | 5 | 3 |  |  |  |
| N classification |  |  |  |  | 0.712 |
| N0 | 45/111 (40.5%) | 66/111 (59.5%) | — | — |  |
| N1 | 60/140 (42.9%) | 80/140 (57.1%) | 1.10 | 0.66, 1.83 |  |
| N2&N3 | 15/30 (50.0%) | 15/30 (50.0%) | 1.47 | 0.65, 3.32 |  |
| Missing | 12 | 22 |  |  |  |
| SBR Grade |  |  |  |  | 0.919 |
| SBR I & II | 62/146 (42.5%) | 84/146 (57.5%) | — | — |  |
| SBR III | 65/151 (43.0%) | 86/151 (57.0%) | 1.02 | 0.65, 1.62 |  |
| Missing | 5 | 13 |  |  |  |
| Presence of vascular emboli |  |  |  |  | 0.175 |
| Yes | 8/25 (32.0%) | 17/25 (68.0%) | — | — |  |
| No | 92/198 (46.5%) | 106/198 (53.5%) | 1.84 | 0.78, 4.70 |  |
| Missing | 32 | 60 |  |  |  |
| Hormonal receptors status |  |  |  |  | 0.026 |
| ER and/or PR + | 67/181 (37.0%) | 114/181 (63.0%) | — | — |  |
| ER and PR - | 60/120 (50.0%) | 60/120 (50.0%) | 1.70 | 1.07, 2.72 |  |
| Missing | 5 | 9 |  |  |  |
| Univariate analysis has been done using a logistic model. P-value is based on a global wald test from logistic model | | | | | |
| ^1^OR = Odds Ratio, CI = Confidence Interval | | | | | |

### Table 6.2.2 pCR result - Multivariate analysis - Full Analysis Set Population

|  | Descriptive statistics | | OR and 95% CI | | |
| --- | --- | --- | --- | --- | --- |
| Characteristic | pCR, N = 132 | No pCR, N = 183 | OR^1^ | 95% CI^1^ | p-value |
| Hormonal receptors status |  |  |  |  | 0.119 |
| ER and/or PR + | 67/181 (37.0%) | 114/181 (63.0%) | — | — |  |
| ER and PR - | 60/120 (50.0%) | 60/120 (50.0%) | 1.55 | 0.89, 2.71 |  |
| Missing | 5 | 9 |  |  |  |
| Presence of vascular emboli |  |  |  |  | 0.138 |
| Yes | 8/25 (32.0%) | 17/25 (68.0%) | — | — |  |
| No | 92/198 (46.5%) | 106/198 (53.5%) | 1.97 | 0.83, 5.06 |  |
| Missing | 32 | 60 |  |  |  |
| Multivariate analysis has been done using a logistic model. P-value is based on a global wald test from logisitc model. The model has been constructed using a stepwise selection of covariates with 0.15 as entry threshold and 0.15 as the retention threshold. | | | | | |
| ^1^OR = Odds Ratio, CI = Confidence Interval | | | | | |

## 6.3 Predictive factors for PFS and pCR result

### Table 6.3.1 Correlation matrix - Full Analysis Set Population

| Variables | Age (years) | Age group (years) | BMI (kg/m2) | T classification | N classification | SBR Grade | Presence of vascular emboli | Hormonal receptors status | pCR results |
| --- | --- | --- | --- | --- | --- | --- | --- | --- | --- |
| Age (years) | ND |  |  |  |  |  |  |  |  |
| Age group (years) | ND | ND |  |  |  |  |  |  |  |
| BMI (kg/m2) | 0.0357 | 0.1529 | ND |  |  |  |  |  |  |
| T classification | 0.0018 | 0.0015 | 0.2264 | ND |  |  |  |  |  |
| N classification | 0.0017 | 0.0095 | 0.1039 | 5e-04 | ND |  |  |  |  |
| SBR Grade | 0.6232 | 0.79 | 0.3445 | 0.4272 | 0.2461 | ND |  |  |  |
| Presence of vascular emboli | 0.9483 | 0.922 | 0.1049 | 0.2244 | 0.043 | 1 | ND |  |  |
| Hormonal receptors status | 0.2834 | 0.151 | 0.9 | 0.2229 | 0.2021 | 0.0012 | 0.3928 | ND |  |
| pCR results | 0.4945 | 0.9704 | 0.5483 | 0.8635 | 0.6484 | 1 | 0.2473 | 0.0345 | ND |
| * pCR results = pCR if ypT0/Tis ypN0 is ticked Yes OR, Grade 1 or Grade 2 are ticked for Classification Chevallier OR, TA and NA are ticked for Classification Sataloff OR, RCB0 is ticked for Classification RCB | | | | | | | | | |
| ND: Not Done | | | | | | | | | |
| Between quantitative and qualitative variables: Anova have been used: the p-value displayed is the p-value of the Type 3 test of fixed effects. P-value is displayed in the above table. | | | | | | | | | |
| Between qualitative variables: Chi² test has been used when all expected counts are >= 5. Otherwise, the Fisher exact test has been used. P-value is displayed in the above table | | | | | | | | | |

### Figure 6.3.2 Correlation coefficient matrix - Full Analysis Set Population


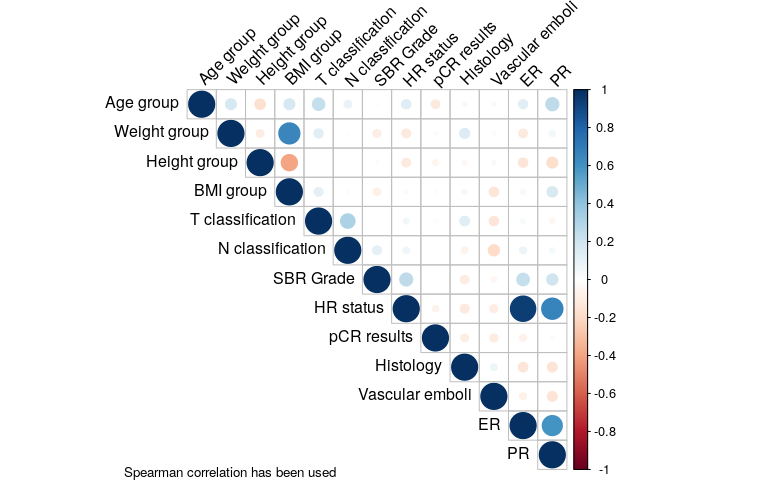

Supplement: S1 File — (ZIP) [file pdig.0000735.s001.zip › Suppl materials self_service_statistical_report.docx]
