# Supplementary material for: Comparison of anonymization techniques regarding statistical reproducibility
Source: PLOS Digit Health. 2025 Feb 3;4(2):e0000735. doi: 10.1371/journal.pdig.0000735 (PMC11790161; doi:10.1371/journal.pdig.0000735)

KADOR PoC Anonymisation – Analysis report on the parm anonymized data

A FRENCH RETROSPECTIVE STUDY DESCRIBING THE EPIDEMIOLOGY AND THE THERAPEUTIC MANAGEMENT OF PATIENTS TREATED BY HERCEPTIN® BASED NEOADJUVANT TREATMENT FOR HER2-POSITIVE EARLY BREAST CANCER

Laetitia Vinet

2022-10-25

Table of Contents

[**1 Analysis of study conduct 1**](#_heading=)

[1.1 Patient Disposition 1](#_heading=)

[Table 1.1.1 Summary of patient disposition - Full Analysis Set Population 1](#_heading=)

[Table 1.1.2 Latest news - Among patients not being followed in the site - Full Analysis Set Population 2](#_heading=)

[Table 1.1.3 Cause of death - Among dead patients - Full Analysis Set Population 4](#_heading=)

[Table 1.1.4 Time from diagnostic to progression - Among patients having experienced progression of the disease since the beginning of adjuvant therapy - Full Analysis Set Population 5](#_heading=)

[Table 1.1.5 Dates available - Full Analysis Set Population 6](#_heading=)

[**2 Baseline characteristics 8**](#_heading=)

[2.1 Demographics and baseline disease characteristics 8](#_heading=)

[Table 2.1.1 Summary of demographics and baseline disease characteristics - Full Analysis Set Population 8](#_heading=)

[Table 2.1.2 Summary of demographics and baseline disease characteristics by pCR result - Full Analysis Set Population 13](#_heading=)

[**3 Surgery and pCR 18**](#_heading=)

[3.1 Surgery 18](#_heading=)

[3.1.1 Summary of surgery - Among patients with at least one surgery - Full Analysis Set Population 18](#_heading=)

[3.2 pCR 19](#_heading=)

[3.2.1 Summary of pCR - Full Analysis Set Population 19](#_heading=)

[**4 Adjuvant treatments 21**](#_heading=)

[Table 4.1 Summary of adjuvant treatments - Among subjects with at least one adjuvant treatments - Full Analysis Set Population 21](#_heading=)

[Table 4.2 Summary of adjuvant treatments by adjuvant treatment - Among subjects with at least one adjuvant treatments - Full Analysis Set Population 23](#_heading=)

[Table 4.3 Time between surgery and adjuvant treatment - Among subjects with at least one adjuvant treatments - Full Analysis Set Population 57](#_heading=)

[Table 4.4 Summary of adjuvant treatments by pCR status - Among subjects with at least one adjuvant treatments - Full Analysis Set Population 58](#_heading=)

[Table 4.5 Summary of adjuvant treatments by adjuvant treatment by pCR status - Among subjects with at least one adjuvant treatments - Full Analysis Set Population 59](#_heading=)

[Table 4.6 Time between surgery and adjuvant treatment by pCR status - Among subjects with at least one adjuvant treatments - Full Analysis Set Population 90](#_heading=)

[**5 Efficacy Analyses 91**](#_heading=)

[5.1 Time to event analyses 91](#_heading=)

[Table 5.1.1 Summary of time from herceptin adjuvant treatment to PFS, overall and by pCR result - Kaplan-Meier estimation - Among subjects with herceptin adjuvant treatment start date available - Full Analysis Set Population 91](#_heading=)

[Table 5.1.2 Survival probabilities of time from herceptin adjuvant treatment to PFS, overall and by pCR result - Kaplan-Meier estimation - Among subjects with herceptin adjuvant treatment start date available - Full Analysis Set Population 92](#_heading=)

[Table 5.1.3 Summary of time from herceptin adjuvant treatment to PFS - Kaplan-Meier curve - Among subjects with herceptin adjuvant treatment start date available - Full Analysis Set Population 93](#_heading=)

[Table 5.1.4 Summary of time from herceptin adjuvant treatment to PFS by pCR result - Kaplan-Meier curve - Among subjects with herceptin adjuvant treatment start date available - Full Analysis Set Population 94](#_heading=)

[**6 Exploratory Analyses 95**](#_heading=)

[6.1 Predictive factors for PFS 95](#_heading=)

[Table 6.1.1 PFS - Univariate Cox proportional hazard analysis - Among subjects with herceptin adjuvant treatment start date available - Full Analysis Set Population 95](#_heading=)

[Table 6.1.2 PFS - Multivariate Cox proportional hazard analysis - Among subjects with herceptin adjuvant treatment start date available - Full Analysis Set Population 96](#_heading=)

[6.2 Predictive factors for pCR result 97](#_heading=)

[Table 6.2.1 pCR result - Univariate analysis - Full Analysis Set Population 97](#_heading=)

[Table 6.2.2 pCR result - Multivariate analysis - Full Analysis Set Population 100](#_heading=)

[6.3 Predictive factors for PFS and pCR result 101](#_heading=)

[Table 6.3.1 Correlation matrix - Full Analysis Set Population 101](#_heading=)

[Figure 6.3.2 Correlation coefficient matrix - Full Analysis Set Population 103](#_heading=)

# 1 Analysis of study conduct

## 1.1 Patient Disposition

### Table 1.1.1 Summary of patient disposition - Full Analysis Set Population

| Characteristic | All (N = 315) |
| --- | --- |
| Follow-up duration (years) |  |
| Nobs | 110 |
| Mean (SD) | 16.86 (21.47) |
| Median (Q1;Q3) | 7.2 (4.4; 11.5) |
| Min - Max | 0.9, 82.4 |
| Missing | 205 |
| Is the patient still being followed in the site (as of December 31, 2018)?, n/N (%) |  |
| Yes | 211/290 (72.8%) |
| No | 79/290 (27.2%) |
| Missing | 25 |
| Time from diagnostic to surgery (months) |  |
| Nobs | 168 |
| Mean (SD) | 159.36 (240.99) |
| Median (Q1;Q3) | 37.2 (18.3; 207.8) |
| Min - Max | 0.2, 978.8 |
| Missing | 147 |
| Has there been any progression of the disease since the beginning of adjuvant therapy, n/N (%) |  |
| Yes | 40/292 (13.7%) |
| No | 252/292 (86.3%) |
| Missing | 23 |
| Follow-up duration (years) = (Last consultation date/Death date – initial diagnosis date of breast cancer + 1) / 365.25 | |
| Time from diagnostic to surgery (months) = (Surgery date – initial diagnosis date of breast cancer) / (365.25/12) | |

### Table 1.1.2 Latest news - Among patients not being followed in the site - Full Analysis Set Population

| Characteristic | All (N = 79) |
| --- | --- |
| Latest news |  |
| The patient is dead | 12/18 (66.7%) |
| The patient is lost to follow-up | 5/18 (27.8%) |
| Other reason | 1/18 (5.6%) |
| Missing | 61 |

### Table 1.1.3 Cause of death - Among dead patients - Full Analysis Set Population

| Characteristic | All (N = 37) |
| --- | --- |
| Cause of death | 0/0 (NA%) |
| Missing | 37 |

### Table 1.1.4 Time from diagnostic to progression - Among patients having experienced progression of the disease since the beginning of adjuvant therapy - Full Analysis Set Population

| Characteristic | All (N = 40) |
| --- | --- |
| Time from diagnostic to progression (years) |  |
| Nobs | 13 |
| Mean (SD) | 22.17 (27.69) |
| Median (Q1;Q3) | 10.5 (4.2; 24.6) |
| Min - Max | 2.1, 86.2 |
| Missing | 27 |
| Time from diagnostic to progression (years) = (Date of the first progression of the disease – initial diagnosis date of breast cancer) / 365.25 | |

### Table 1.1.5 Dates available - Full Analysis Set Population

| Characteristic | All (N = 315) |
| --- | --- |
| Birth date available |  |
| Yes | 315/315 (100.0%) |
| No | 0/315 (0.0%) |
| Initial diagnosis date of breast cancer available |  |
| Yes | 269/315 (85.4%) |
| No | 46/315 (14.6%) |
| Surgery date available |  |
| Yes | 210/315 (66.7%) |
| No | 105/315 (33.3%) |
| Date of last consultation available among patients still being followed in the site (as of December 31, 2018) |  |
| Yes | 123/211 (58.3%) |
| No | 88/211 (41.7%) |
| Date of the first progression of the disease available among patients having experienced progression of the disease since the beginning of adjuvant therapy |  |
| Yes | 14/40 (35.0%) |
| No | 26/40 (65.0%) |
| Death date available among dead patients |  |
| Yes | 13/37 (35.1%) |
| No | 24/37 (64.9%) |

# 2 Baseline characteristics

## 2.1 Demographics and baseline disease characteristics

### Table 2.1.1 Summary of demographics and baseline disease characteristics - Full Analysis Set Population

| Characteristic | All (N = 315) |
| --- | --- |
| Age at adjuvant treatment initiation of Herceptin (years) |  |
| Nobs | 0 |
| Mean (SD) | NA (NA) |
| Median (Q1;Q3) | NA (NA; NA) |
| Min - Max | Inf, -Inf |
| Missing | 315 |
| Age group (years), n/N (%) | 0/0 (NA%) |
| Missing | 315 |
| BMI (kg/m2), n/N (%) |  |
| <25 | 98/315 (31.1%) |
| [25 - 30[ | 114/315 (36.2%) |
| >=30 | 103/315 (32.7%) |
| Missing | 0 |
| Professional situation, n/N (%) |  |
| Worker | 159/315 (50.5%) |
| Data not found | 90/315 (28.6%) |
| Jobless person | 66/315 (21.0%) |
| Missing | 0 |
| Weight (kg) |  |
| Nobs | 315 |
| Mean (SD) | 72.74 (17.45) |
| Median (Q1;Q3) | 69.9 (60.6; 78.8) |
| Min - Max | 40, 147 |
| Missing | 0 |
| Height(cm) |  |
| Nobs | 315 |
| Mean (SD) | 160.39 (6.97) |
| Median (Q1;Q3) | 159.9 (155.3; 164.6) |
| Min - Max | 146, 179 |
| Missing | 0 |
| Weight at initiation of adjuvant therapy (kg) |  |
| Nobs | 235 |
| Mean (SD) | 72.62 (11.39) |
| Median (Q1;Q3) | 71.4 (66.5; 76.3) |
| Min - Max | 45, 129 |
| Missing | 80 |
| Classification T, n/N (%) |  |
| T0 | 1/312 (0.3%) |
| T1a | 4/312 (1.3%) |
| T1b | 1/312 (0.3%) |
| T1c | 21/312 (6.7%) |
| T2 | 141/312 (45.2%) |
| T3 | 71/312 (22.8%) |
| T4a | 13/312 (4.2%) |
| T4b | 12/312 (3.8%) |
| T4c | 2/312 (0.6%) |
| T4d | 39/312 (12.5%) |
| TX | 7/312 (2.2%) |
| Missing | 3 |
| Classification N, n/N (%) |  |
| N0 | 108/301 (35.9%) |
| N1 | 131/301 (43.5%) |
| N2 | 23/301 (7.6%) |
| N3 | 4/301 (1.3%) |
| NX | 35/301 (11.6%) |
| Missing | 14 |
| Histology at the initial diagnosis, n/N (%) |  |
| Invasive ductal carcinoma | 275/306 (89.9%) |
| Invasive lobular carcinoma | 9/306 (2.9%) |
| Mixed carcinoma | 4/306 (1.3%) |
| Other | 12/306 (3.9%) |
| Unknown | 6/306 (2.0%) |
| Missing | 9 |
| Presence of vascular emboli, n/N (%) |  |
| Yes | 29/206 (14.1%) |
| No | 177/206 (85.9%) |
| Missing | 109 |
| SBR grade, n/N (%) |  |
| SBR I | 9/296 (3.0%) |
| SBR II | 133/296 (44.9%) |
| SBR III | 149/296 (50.3%) |
| Ungradable | 5/296 (1.7%) |
| Missing | 19 |
| Number of lymph nodes invaded |  |
| Nobs | 212 |
| Mean (SD) | 1.02 (1.85) |
| Median (Q1;Q3) | 0.0 (0.0; 1.0) |
| Min - Max | 0, 10 |
| Missing | 103 |
| Estrogen receptors, n/N (%) |  |
| positive | 181/300 (60.3%) |
| negative | 119/300 (39.7%) |
| not assessable | 0/300 (0.0%) |
| Missing | 15 |
| Progesterone receptors, n/N (%) |  |
| positive | 130/282 (46.1%) |
| negative | 152/282 (53.9%) |
| not assessable | 0/282 (0.0%) |
| Missing | 33 |
| Hormonal receptors status, n/N (%) |  |
| ER and/or PR + | 241/301 (80.1%) |
| ER and PR - | 60/301 (19.9%) |
| Missing | 14 |

### Table 2.1.2 Summary of demographics and baseline disease characteristics by pCR result - Full Analysis Set Population

| Characteristic | pCR (N = 108) | No pCR (N = 207) |
| --- | --- | --- |
| Age at adjuvant treatment initiation of Herceptin (years) |  |  |
| Nobs | 0 | 0 |
| Mean (SD) | NA (NA) | NA (NA) |
| Median (Q1;Q3) | NA (NA; NA) | NA (NA; NA) |
| Min - Max | Inf, -Inf | Inf, -Inf |
| Missing | 108 | 207 |
| Age group (years), n/N (%) | 0/0 (NA%) | 0/0 (NA%) |
| Missing | 108 | 207 |
| BMI (kg/m2), n/N (%) |  |  |
| <25 | 37/108 (34.3%) | 61/207 (29.5%) |
| [25 - 30[ | 39/108 (36.1%) | 75/207 (36.2%) |
| >=30 | 32/108 (29.6%) | 71/207 (34.3%) |
| Missing | 0 | 0 |
| Professional situation, n/N (%) |  |  |
| Worker | 60/108 (55.6%) | 99/207 (47.8%) |
| Data not found | 30/108 (27.8%) | 60/207 (29.0%) |
| Jobless person | 18/108 (16.7%) | 48/207 (23.2%) |
| Missing | 0 | 0 |
| Weight (kg) |  |  |
| Nobs | 108 | 207 |
| Mean (SD) | 72.60 (17.36) | 72.82 (17.54) |
| Median (Q1;Q3) | 69.5 (60.1; 77.7) | 69.9 (61.3; 80.2) |
| Min - Max | 50, 137 | 40, 147 |
| Missing | 0 | 0 |
| Height(cm) |  |  |
| Nobs | 108 | 207 |
| Mean (SD) | 159.81 (7.51) | 160.70 (6.67) |
| Median (Q1;Q3) | 159.3 (154.2; 164.4) | 160.5 (155.6; 164.9) |
| Min - Max | 146, 179 | 147, 179 |
| Missing | 0 | 0 |
| Weight at initiation of adjuvant therapy (kg) |  |  |
| Nobs | 79 | 156 |
| Mean (SD) | 72.53 (11.41) | 72.67 (11.42) |
| Median (Q1;Q3) | 71.4 (65.8; 76.3) | 71.4 (67.0; 76.1) |
| Min - Max | 50, 127 | 45, 129 |
| Missing | 29 | 51 |
| Classification T, n/N (%) |  |  |
| T0 | 0/106 (0.0%) | 1/206 (0.5%) |
| T1a | 2/106 (1.9%) | 2/206 (1.0%) |
| T1b | 0/106 (0.0%) | 1/206 (0.5%) |
| T1c | 6/106 (5.7%) | 15/206 (7.3%) |
| T2 | 50/106 (47.2%) | 91/206 (44.2%) |
| T3 | 26/106 (24.5%) | 45/206 (21.8%) |
| T4a | 3/106 (2.8%) | 10/206 (4.9%) |
| T4b | 4/106 (3.8%) | 8/206 (3.9%) |
| T4c | 0/106 (0.0%) | 2/206 (1.0%) |
| T4d | 12/106 (11.3%) | 27/206 (13.1%) |
| TX | 3/106 (2.8%) | 4/206 (1.9%) |
| Missing | 2 | 1 |
| Classification N, n/N (%) |  |  |
| N0 | 37/105 (35.2%) | 71/196 (36.2%) |
| N1 | 46/105 (43.8%) | 85/196 (43.4%) |
| N2 | 7/105 (6.7%) | 16/196 (8.2%) |
| N3 | 2/105 (1.9%) | 2/196 (1.0%) |
| NX | 13/105 (12.4%) | 22/196 (11.2%) |
| Missing | 3 | 11 |
| Histology at the initial diagnosis, n/N (%) |  |  |
| Invasive ductal carcinoma | 97/106 (91.5%) | 178/200 (89.0%) |
| Invasive lobular carcinoma | 1/106 (0.9%) | 8/200 (4.0%) |
| Mixed carcinoma | 1/106 (0.9%) | 3/200 (1.5%) |
| Other | 4/106 (3.8%) | 8/200 (4.0%) |
| Unknown | 3/106 (2.8%) | 3/200 (1.5%) |
| Missing | 2 | 7 |
| Presence of vascular emboli, n/N (%) |  |  |
| Yes | 13/76 (17.1%) | 16/130 (12.3%) |
| No | 63/76 (82.9%) | 114/130 (87.7%) |
| Missing | 32 | 77 |
| SBR grade, n/N (%) |  |  |
| SBR I | 3/103 (2.9%) | 6/193 (3.1%) |
| SBR II | 44/103 (42.7%) | 89/193 (46.1%) |
| SBR III | 54/103 (52.4%) | 95/193 (49.2%) |
| Ungradable | 2/103 (1.9%) | 3/193 (1.6%) |
| Missing | 5 | 14 |
| Number of lymph nodes invaded |  |  |
| Nobs | 75 | 137 |
| Mean (SD) | 0.99 (1.54) | 1.04 (2.01) |
| Median (Q1;Q3) | 1.0 (0.0; 1.0) | 0.0 (0.0; 1.0) |
| Min - Max | 0, 8 | 0, 10 |
| Missing | 33 | 70 |
| Estrogen receptors, n/N (%) |  |  |
| positive | 65/105 (61.9%) | 116/195 (59.5%) |
| negative | 40/105 (38.1%) | 79/195 (40.5%) |
| not assessable | 0/105 (0.0%) | 0/195 (0.0%) |
| Missing | 3 | 12 |
| Progesterone receptors, n/N (%) |  |  |
| positive | 47/100 (47.0%) | 83/182 (45.6%) |
| negative | 53/100 (53.0%) | 99/182 (54.4%) |
| not assessable | 0/100 (0.0%) | 0/182 (0.0%) |
| Missing | 8 | 25 |
| Hormonal receptors status, n/N (%) |  |  |
| ER and/or PR + | 86/105 (81.9%) | 155/196 (79.1%) |
| ER and PR - | 19/105 (18.1%) | 41/196 (20.9%) |
| Missing | 3 | 11 |

# 3 Surgery and pCR

## 3.1 Surgery

### 3.1.1 Summary of surgery - Among patients with at least one surgery - Full Analysis Set Population

| Characteristic | All (N = 250) |
| --- | --- |
| At least one Surgery* |  |
| Axillary curage | 167 (66.8%) |
| Conservative surgery | 116 (46.4%) |
| Mastectomy | 78 (31.2%) |
| Sentinel Ganglion | 9 (3.6%) |
| * One patient can have reported several surgery types | |

## 3.2 pCR

### 3.2.1 Summary of pCR - Full Analysis Set Population

| Characteristic | All (N = 315) |
| --- | --- |
| pCR results* |  |
| pCR | 108/315 (34.3%) |
| No pCR | 207/315 (65.7%) |
| Missing | 0 |
| Absence of invasive and in situ residues in the breast and in the lymph nodes |  |
| Yes | 88/233 (37.8%) |
| No | 145/233 (62.2%) |
| Missing | 82 |
| Absence of invasive residues in the breast and lymph nodes, regardless of the presence of ductal carcinoma in situ |  |
| Yes | 101/237 (42.6%) |
| No | 136/237 (57.4%) |
| Missing | 78 |
| Classification Chevallier |  |
| Grade 1 | 1/17 (5.9%) |
| Grade 2 | 7/17 (41.2%) |
| Grade 3 | 9/17 (52.9%) |
| Missing | 298 |
| Classification Sataloff T |  |
| TA | 38/60 (63.3%) |
| TB | 17/60 (28.3%) |
| TC | 5/60 (8.3%) |
| Missing | 255 |
| Classification Sataloff N |  |
| NA | 20/61 (32.8%) |
| NB | 29/61 (47.5%) |
| NC | 7/61 (11.5%) |
| ND | 5/61 (8.2%) |
| Missing | 254 |
| Classification RCB |  |
| RCB-I | 1/6 (16.7%) |
| RCB-II | 5/6 (83.3%) |
| Missing | 309 |
| * pCR results = pCR if ypT0/Tis ypN0 is ticked Yes OR, Grade 1 or Grade 2 are ticked for Classification Chevallier OR, TA and NA are ticked for Classification Sataloff OR, RCB0 is ticked for Classification RCB | |

# 4 Adjuvant treatments

## Table 4.1 Summary of adjuvant treatments - Among subjects with at least one adjuvant treatments - Full Analysis Set Population

| Characteristic | All (N = 243) |
| --- | --- |
| At least one Adjuvant Treatment* |  |
| Docetaxel | 41 (16.9%) |
| Other | 38 (15.6%) |
| Carboplatine | 35 (14.4%) |
| Anastrozole | 34 (14%) |
| Tamoxifene | 34 (14%) |
| Paclitaxel | 33 (13.6%) |
| Letrozole | 28 (11.5%) |
| Navelbine | 28 (11.5%) |
| Cyclophosphamide | 27 (11.1%) |
| Epirubicine | 26 (10.7%) |
| Exemestane | 26 (10.7%) |
| Doxorubicine | 25 (10.3%) |
| Other hormonotherapy 1 | 25 (10.3%) |
| Trastuzumab (Herceptin) | 25 (10.3%) |
| 5-FU | 22 (9.1%) |
| Other hormonotherapy 2 | 17 (7%) |
| * One patient can have reported several adjuvant treatment types | |

## Table 4.2 Summary of adjuvant treatments by adjuvant treatment - Among subjects with at least one adjuvant treatments - Full Analysis Set Population

| Characteristic | All |
| --- | --- |
| 5-FU :  - Duration (months) |  |
| Nobs | 0 |
| Mean (SD) | NA (NA) |
| Median (Q1;Q3) | NA (NA; NA) |
| Min - Max | NA, NA |
| Missing | 22 |
| - Administration frequency |  |
| Nobs | 3 |
| Mean (SD) | 1.00 (0.00) |
| Median (Q1;Q3) | 1.0 (1.0; 1.0) |
| Min - Max | 1.0, 1.0 |
| Missing | 19 |
| - Maintenance dose (cycle) |  |
| Nobs | 3 |
| Mean (SD) | 837.33 (1,439.91) |
| Median (Q1;Q3) | 6.0 (6.0; 1,253.0) |
| Min - Max | 6.0, 2,500.0 |
| Missing | 19 |
| - Maintenance dose (cycle mg/kg or mg) |  |
| Nobs | 1 |
| Mean (SD) | 20.00 (NA) |
| Median (Q1;Q3) | 20.0 (20.0; 20.0) |
| Min - Max | 20.0, 20.0 |
| Missing | 21 |
| - Route of administration, n/N (%) |  |
| Intravenous | 4/5 (80.0%) |
| Subcutaneous | 1/5 (20.0%) |
| Missing | 17 |
| - Number of cycles completed |  |
| Nobs | 6 |
| Mean (SD) | 8.83 (7.63) |
| Median (Q1;Q3) | 7.5 (2.0; 16.0) |
| Min - Max | 2.0, 17.0 |
| Missing | 16 |
| - Location of administration, n/N (%) |  |
| Hospital | 3/3 (100.0%) |
| Missing | 19 |
| - Start date of treatment available, n/N (%) |  |
| Yes | 0/22 (0.0%) |
| No | 22/22 (100.0%) |
| - End date of treatment available, n/N (%) |  |
| No | 21/21 (100.0%) |
| Anastrozole :  - Duration (months) |  |
| Nobs | 0 |
| Mean (SD) | NA (NA) |
| Median (Q1;Q3) | NA (NA; NA) |
| Min - Max | NA, NA |
| Missing | 34 |
| - Administration frequency |  |
| Nobs | 3 |
| Mean (SD) | 3.00 (0.00) |
| Median (Q1;Q3) | 3.0 (3.0; 3.0) |
| Min - Max | 3.0, 3.0 |
| Missing | 31 |
| - Maintenance dose (cycle) |  |
| Nobs | 3 |
| Mean (SD) | 1,668.67 (1,439.91) |
| Median (Q1;Q3) | 2,500.0 (1,253.0; 2,500.0) |
| Min - Max | 6.0, 2,500.0 |
| Missing | 31 |
| - Maintenance dose (cycle mg/kg or mg) |  |
| Nobs | 0 |
| Mean (SD) | NA (NA) |
| Median (Q1;Q3) | NA (NA; NA) |
| Min - Max | NA, NA |
| Missing | 34 |
| - Route of administration, n/N (%) |  |
| Intravenous | 2/2 (100.0%) |
| Missing | 32 |
| - Number of cycles completed |  |
| Nobs | 3 |
| Mean (SD) | 2.00 (0.00) |
| Median (Q1;Q3) | 2.0 (2.0; 2.0) |
| Min - Max | 2.0, 2.0 |
| Missing | 31 |
| - Location of administration, n/N (%) |  |
| Hospital | 1/1 (100.0%) |
| Missing | 33 |
| - Start date of treatment available, n/N (%) |  |
| Yes | 0/34 (0.0%) |
| No | 34/34 (100.0%) |
| - End date of treatment available, n/N (%) |  |
| No | 34/34 (100.0%) |
| Carboplatine :  - Duration (months) |  |
| Nobs | 0 |
| Mean (SD) | NA (NA) |
| Median (Q1;Q3) | NA (NA; NA) |
| Min - Max | NA, NA |
| Missing | 35 |
| - Administration frequency |  |
| Nobs | 3 |
| Mean (SD) | 1.67 (1.15) |
| Median (Q1;Q3) | 1.0 (1.0; 2.0) |
| Min - Max | 1.0, 3.0 |
| Missing | 32 |
| - Maintenance dose (cycle) |  |
| Nobs | 3 |
| Mean (SD) | 837.33 (1,439.91) |
| Median (Q1;Q3) | 6.0 (6.0; 1,253.0) |
| Min - Max | 6.0, 2,500.0 |
| Missing | 32 |
| - Maintenance dose (cycle mg/kg or mg) |  |
| Nobs | 3 |
| Mean (SD) | 120.33 (190.47) |
| Median (Q1;Q3) | 20.0 (10.5; 180.0) |
| Min - Max | 1.0, 340.0 |
| Missing | 32 |
| - Route of administration, n/N (%) |  |
| Intravenous | 5/5 (100.0%) |
| Missing | 30 |
| - Number of cycles completed |  |
| Nobs | 5 |
| Mean (SD) | 8.80 (7.16) |
| Median (Q1;Q3) | 8.0 (2.0; 14.0) |
| Min - Max | 2.0, 18.0 |
| Missing | 30 |
| - Location of administration, n/N (%) |  |
| Hospital | 3/3 (100.0%) |
| Missing | 32 |
| - Start date of treatment available, n/N (%) |  |
| Yes | 0/35 (0.0%) |
| No | 35/35 (100.0%) |
| - End date of treatment available, n/N (%) |  |
| No | 32/32 (100.0%) |
| Cyclophosphamide :  - Duration (months) |  |
| Nobs | 0 |
| Mean (SD) | NA (NA) |
| Median (Q1;Q3) | NA (NA; NA) |
| Min - Max | NA, NA |
| Missing | 27 |
| - Administration frequency |  |
| Nobs | 3 |
| Mean (SD) | 3.67 (0.58) |
| Median (Q1;Q3) | 4.0 (3.5; 4.0) |
| Min - Max | 3.0, 4.0 |
| Missing | 24 |
| - Maintenance dose (cycle) |  |
| Nobs | 2 |
| Mean (SD) | 1,253.00 (1,763.52) |
| Median (Q1;Q3) | 1,253.0 (629.5; 1,876.5) |
| Min - Max | 6.0, 2,500.0 |
| Missing | 25 |
| - Maintenance dose (cycle mg/kg or mg) |  |
| Nobs | 2 |
| Mean (SD) | 22.50 (3.54) |
| Median (Q1;Q3) | 22.5 (21.2; 23.8) |
| Min - Max | 20.0, 25.0 |
| Missing | 25 |
| - Route of administration, n/N (%) |  |
| Intravenous | 2/2 (100.0%) |
| Missing | 25 |
| - Number of cycles completed |  |
| Nobs | 4 |
| Mean (SD) | 8.00 (6.93) |
| Median (Q1;Q3) | 8.0 (2.0; 14.0) |
| Min - Max | 2.0, 14.0 |
| Missing | 23 |
| - Location of administration, n/N (%) |  |
| Hospital | 2/2 (100.0%) |
| Missing | 25 |
| - Start date of treatment available, n/N (%) |  |
| Yes | 0/27 (0.0%) |
| No | 27/27 (100.0%) |
| - End date of treatment available, n/N (%) |  |
| No | 21/21 (100.0%) |
| Docetaxel :  - Duration (months) |  |
| Nobs | 0 |
| Mean (SD) | NA (NA) |
| Median (Q1;Q3) | NA (NA; NA) |
| Min - Max | NA, NA |
| Missing | 41 |
| - Administration frequency |  |
| Nobs | 7 |
| Mean (SD) | 1.71 (1.25) |
| Median (Q1;Q3) | 1.0 (1.0; 2.0) |
| Min - Max | 1.0, 4.0 |
| Missing | 34 |
| - Maintenance dose (cycle) |  |
| Nobs | 3 |
| Mean (SD) | 836.00 (1,441.07) |
| Median (Q1;Q3) | 6.0 (4.0; 1,253.0) |
| Min - Max | 2.0, 2,500.0 |
| Missing | 38 |
| - Maintenance dose (cycle mg/kg or mg) |  |
| Nobs | 2 |
| Mean (SD) | 600.00 (0.00) |
| Median (Q1;Q3) | 600.0 (600.0; 600.0) |
| Min - Max | 600.0, 600.0 |
| Missing | 39 |
| - Route of administration, n/N (%) |  |
| Intravenous | 4/4 (100.0%) |
| Missing | 37 |
| - Number of cycles completed |  |
| Nobs | 4 |
| Mean (SD) | 7.75 (6.65) |
| Median (Q1;Q3) | 7.5 (2.0; 13.2) |
| Min - Max | 2.0, 14.0 |
| Missing | 37 |
| - Location of administration, n/N (%) |  |
| Hospital | 2/2 (100.0%) |
| Missing | 39 |
| - Start date of treatment available, n/N (%) |  |
| Yes | 0/41 (0.0%) |
| No | 41/41 (100.0%) |
| - End date of treatment available, n/N (%) |  |
| No | 40/40 (100.0%) |
| Doxorubicine :  - Duration (months) |  |
| Nobs | 0 |
| Mean (SD) | NA (NA) |
| Median (Q1;Q3) | NA (NA; NA) |
| Min - Max | NA, NA |
| Missing | 25 |
| - Administration frequency |  |
| Nobs | 2 |
| Mean (SD) | 2.00 (1.41) |
| Median (Q1;Q3) | 2.0 (1.5; 2.5) |
| Min - Max | 1.0, 3.0 |
| Missing | 23 |
| - Maintenance dose (cycle) |  |
| Nobs | 5 |
| Mean (SD) | 504.80 (1,115.35) |
| Median (Q1;Q3) | 6.0 (6.0; 6.0) |
| Min - Max | 6.0, 2,500.0 |
| Missing | 20 |
| - Maintenance dose (cycle mg/kg or mg) |  |
| Nobs | 1 |
| Mean (SD) | 340.00 (NA) |
| Median (Q1;Q3) | 340.0 (340.0; 340.0) |
| Min - Max | 340.0, 340.0 |
| Missing | 24 |
| - Route of administration, n/N (%) |  |
| Intravenous | 2/2 (100.0%) |
| Missing | 23 |
| - Number of cycles completed |  |
| Nobs | 2 |
| Mean (SD) | 2.00 (0.00) |
| Median (Q1;Q3) | 2.0 (2.0; 2.0) |
| Min - Max | 2.0, 2.0 |
| Missing | 23 |
| - Location of administration, n/N (%) |  |
| Hospital | 3/3 (100.0%) |
| Missing | 22 |
| - Start date of treatment available, n/N (%) |  |
| Yes | 0/25 (0.0%) |
| No | 25/25 (100.0%) |
| - End date of treatment available, n/N (%) |  |
| No | 24/24 (100.0%) |
| Epirubicine :  - Duration (months) |  |
| Nobs | 0 |
| Mean (SD) | NA (NA) |
| Median (Q1;Q3) | NA (NA; NA) |
| Min - Max | NA, NA |
| Missing | 26 |
| - Administration frequency |  |
| Nobs | 5 |
| Mean (SD) | 1.40 (0.89) |
| Median (Q1;Q3) | 1.0 (1.0; 1.0) |
| Min - Max | 1.0, 3.0 |
| Missing | 21 |
| - Maintenance dose (cycle) |  |
| Nobs | 2 |
| Mean (SD) | 2,500.00 (0.00) |
| Median (Q1;Q3) | 2,500.0 (2,500.0; 2,500.0) |
| Min - Max | 2,500.0, 2,500.0 |
| Missing | 24 |
| - Maintenance dose (cycle mg/kg or mg) |  |
| Nobs | 0 |
| Mean (SD) | NA (NA) |
| Median (Q1;Q3) | NA (NA; NA) |
| Min - Max | NA, NA |
| Missing | 26 |
| - Route of administration, n/N (%) |  |
| Intravenous | 2/2 (100.0%) |
| Missing | 24 |
| - Number of cycles completed |  |
| Nobs | 3 |
| Mean (SD) | 6.00 (6.93) |
| Median (Q1;Q3) | 2.0 (2.0; 8.0) |
| Min - Max | 2.0, 14.0 |
| Missing | 23 |
| - Location of administration, n/N (%) |  |
| Hospital | 2/2 (100.0%) |
| Missing | 24 |
| - Start date of treatment available, n/N (%) |  |
| Yes | 0/26 (0.0%) |
| No | 26/26 (100.0%) |
| - End date of treatment available, n/N (%) |  |
| No | 24/24 (100.0%) |
| Exemestane :  - Duration (months) |  |
| Nobs | 0 |
| Mean (SD) | NA (NA) |
| Median (Q1;Q3) | NA (NA; NA) |
| Min - Max | NA, NA |
| Missing | 26 |
| - Administration frequency |  |
| Nobs | 5 |
| Mean (SD) | 1.80 (1.10) |
| Median (Q1;Q3) | 1.0 (1.0; 3.0) |
| Min - Max | 1.0, 3.0 |
| Missing | 21 |
| - Maintenance dose (cycle) |  |
| Nobs | 4 |
| Mean (SD) | 629.50 (1,247.00) |
| Median (Q1;Q3) | 6.0 (6.0; 629.5) |
| Min - Max | 6.0, 2,500.0 |
| Missing | 22 |
| - Maintenance dose (cycle mg/kg or mg) |  |
| Nobs | 0 |
| Mean (SD) | NA (NA) |
| Median (Q1;Q3) | NA (NA; NA) |
| Min - Max | NA, NA |
| Missing | 26 |
| - Route of administration, n/N (%) |  |
| Intravenous | 4/4 (100.0%) |
| Missing | 22 |
| - Number of cycles completed |  |
| Nobs | 2 |
| Mean (SD) | 10.00 (11.31) |
| Median (Q1;Q3) | 10.0 (6.0; 14.0) |
| Min - Max | 2.0, 18.0 |
| Missing | 24 |
| - Location of administration, n/N (%) |  |
| Hospital | 2/2 (100.0%) |
| Missing | 24 |
| - Start date of treatment available, n/N (%) |  |
| Yes | 0/26 (0.0%) |
| No | 26/26 (100.0%) |
| - End date of treatment available, n/N (%) |  |
| No | 24/24 (100.0%) |
| Letrozole :  - Duration (months) |  |
| Nobs | 0 |
| Mean (SD) | NA (NA) |
| Median (Q1;Q3) | NA (NA; NA) |
| Min - Max | NA, NA |
| Missing | 28 |
| - Administration frequency |  |
| Nobs | 4 |
| Mean (SD) | 2.50 (1.00) |
| Median (Q1;Q3) | 3.0 (2.5; 3.0) |
| Min - Max | 1.0, 3.0 |
| Missing | 24 |
| - Maintenance dose (cycle) |  |
| Nobs | 7 |
| Mean (SD) | 720.57 (1,215.59) |
| Median (Q1;Q3) | 6.0 (6.0; 1,260.0) |
| Min - Max | 6.0, 2,500.0 |
| Missing | 21 |
| - Maintenance dose (cycle mg/kg or mg) |  |
| Nobs | 1 |
| Mean (SD) | 600.00 (NA) |
| Median (Q1;Q3) | 600.0 (600.0; 600.0) |
| Min - Max | 600.0, 600.0 |
| Missing | 27 |
| - Route of administration, n/N (%) |  |
| Intravenous | 2/3 (66.7%) |
| Subcutaneous | 1/3 (33.3%) |
| Missing | 25 |
| - Number of cycles completed |  |
| Nobs | 1 |
| Mean (SD) | 2.00 (NA) |
| Median (Q1;Q3) | 2.0 (2.0; 2.0) |
| Min - Max | 2.0, 2.0 |
| Missing | 27 |
| - Location of administration, n/N (%) |  |
| Hospital | 3/3 (100.0%) |
| Missing | 25 |
| - Start date of treatment available, n/N (%) |  |
| Yes | 0/28 (0.0%) |
| No | 28/28 (100.0%) |
| - End date of treatment available, n/N (%) |  |
| No | 25/25 (100.0%) |
| Navelbine :  - Duration (months) |  |
| Nobs | 0 |
| Mean (SD) | NA (NA) |
| Median (Q1;Q3) | NA (NA; NA) |
| Min - Max | NA, NA |
| Missing | 28 |
| - Administration frequency |  |
| Nobs | 5 |
| Mean (SD) | 3.40 (0.55) |
| Median (Q1;Q3) | 3.0 (3.0; 4.0) |
| Min - Max | 3.0, 4.0 |
| Missing | 23 |
| - Maintenance dose (cycle) |  |
| Nobs | 2 |
| Mean (SD) | 2,500.00 (0.00) |
| Median (Q1;Q3) | 2,500.0 (2,500.0; 2,500.0) |
| Min - Max | 2,500.0, 2,500.0 |
| Missing | 26 |
| - Maintenance dose (cycle mg/kg or mg) |  |
| Nobs | 0 |
| Mean (SD) | NA (NA) |
| Median (Q1;Q3) | NA (NA; NA) |
| Min - Max | NA, NA |
| Missing | 28 |
| - Route of administration, n/N (%) |  |
| Intravenous | 6/6 (100.0%) |
| Missing | 22 |
| - Number of cycles completed |  |
| Nobs | 5 |
| Mean (SD) | 3.80 (4.02) |
| Median (Q1;Q3) | 2.0 (2.0; 2.0) |
| Min - Max | 2.0, 11.0 |
| Missing | 23 |
| - Location of administration, n/N (%) |  |
| Hospital | 3/3 (100.0%) |
| Missing | 25 |
| - Start date of treatment available, n/N (%) |  |
| Yes | 0/28 (0.0%) |
| No | 28/28 (100.0%) |
| - End date of treatment available, n/N (%) |  |
| No | 25/25 (100.0%) |
| Other :  - Duration (months) |  |
| Nobs | 0 |
| Mean (SD) | NA (NA) |
| Median (Q1;Q3) | NA (NA; NA) |
| Min - Max | NA, NA |
| Missing | 38 |
| - Administration frequency |  |
| Nobs | 8 |
| Mean (SD) | 1.50 (0.93) |
| Median (Q1;Q3) | 1.0 (1.0; 1.5) |
| Min - Max | 1.0, 3.0 |
| Missing | 30 |
| - Maintenance dose (cycle) |  |
| Nobs | 1 |
| Mean (SD) | 6.00 (NA) |
| Median (Q1;Q3) | 6.0 (6.0; 6.0) |
| Min - Max | 6.0, 6.0 |
| Missing | 37 |
| - Maintenance dose (cycle mg/kg or mg) |  |
| Nobs | 1 |
| Mean (SD) | 600.00 (NA) |
| Median (Q1;Q3) | 600.0 (600.0; 600.0) |
| Min - Max | 600.0, 600.0 |
| Missing | 37 |
| - Route of administration, n/N (%) |  |
| Intravenous | 4/4 (100.0%) |
| Missing | 34 |
| - Number of cycles completed |  |
| Nobs | 5 |
| Mean (SD) | 8.80 (6.26) |
| Median (Q1;Q3) | 12.0 (2.0; 14.0) |
| Min - Max | 2.0, 14.0 |
| Missing | 33 |
| - Location of administration, n/N (%) |  |
| Home | 1/4 (25.0%) |
| Hospital | 3/4 (75.0%) |
| Missing | 34 |
| - Start date of treatment available, n/N (%) |  |
| Yes | 0/38 (0.0%) |
| No | 38/38 (100.0%) |
| - End date of treatment available, n/N (%) |  |
| No | 35/35 (100.0%) |
| Other hormonotherapy 1 :  - Duration (months) |  |
| Nobs | 0 |
| Mean (SD) | NA (NA) |
| Median (Q1;Q3) | NA (NA; NA) |
| Min - Max | NA, NA |
| Missing | 25 |
| - Administration frequency |  |
| Nobs | 2 |
| Mean (SD) | 3.00 (0.00) |
| Median (Q1;Q3) | 3.0 (3.0; 3.0) |
| Min - Max | 3.0, 3.0 |
| Missing | 23 |
| - Maintenance dose (cycle) |  |
| Nobs | 2 |
| Mean (SD) | 1,253.00 (1,763.52) |
| Median (Q1;Q3) | 1,253.0 (629.5; 1,876.5) |
| Min - Max | 6.0, 2,500.0 |
| Missing | 23 |
| - Maintenance dose (cycle mg/kg or mg) |  |
| Nobs | 0 |
| Mean (SD) | NA (NA) |
| Median (Q1;Q3) | NA (NA; NA) |
| Min - Max | NA, NA |
| Missing | 25 |
| - Route of administration, n/N (%) |  |
| Intravenous | 3/4 (75.0%) |
| Subcutaneous | 1/4 (25.0%) |
| Missing | 21 |
| - Number of cycles completed |  |
| Nobs | 2 |
| Mean (SD) | 2.00 (0.00) |
| Median (Q1;Q3) | 2.0 (2.0; 2.0) |
| Min - Max | 2.0, 2.0 |
| Missing | 23 |
| - Location of administration, n/N (%) |  |
| Hospital | 2/2 (100.0%) |
| Missing | 23 |
| - Start date of treatment available, n/N (%) |  |
| Yes | 0/25 (0.0%) |
| No | 25/25 (100.0%) |
| - End date of treatment available, n/N (%) |  |
| No | 24/24 (100.0%) |
| Other hormonotherapy 2 :  - Duration (months) |  |
| Nobs | 0 |
| Mean (SD) | NA (NA) |
| Median (Q1;Q3) | NA (NA; NA) |
| Min - Max | NA, NA |
| Missing | 17 |
| - Administration frequency |  |
| Nobs | 1 |
| Mean (SD) | 1.00 (NA) |
| Median (Q1;Q3) | 1.0 (1.0; 1.0) |
| Min - Max | 1.0, 1.0 |
| Missing | 16 |
| - Maintenance dose (cycle) |  |
| Nobs | 0 |
| Mean (SD) | NA (NA) |
| Median (Q1;Q3) | NA (NA; NA) |
| Min - Max | NA, NA |
| Missing | 17 |
| - Maintenance dose (cycle mg/kg or mg) |  |
| Nobs | 0 |
| Mean (SD) | NA (NA) |
| Median (Q1;Q3) | NA (NA; NA) |
| Min - Max | NA, NA |
| Missing | 17 |
| - Route of administration, n/N (%) |  |
| Intravenous | 4/4 (100.0%) |
| Missing | 13 |
| - Number of cycles completed |  |
| Nobs | 4 |
| Mean (SD) | 7.75 (6.65) |
| Median (Q1;Q3) | 7.5 (2.0; 13.2) |
| Min - Max | 2.0, 14.0 |
| Missing | 13 |
| - Location of administration, n/N (%) |  |
| Hospital | 2/2 (100.0%) |
| Missing | 15 |
| - Start date of treatment available, n/N (%) |  |
| Yes | 0/17 (0.0%) |
| No | 17/17 (100.0%) |
| - End date of treatment available, n/N (%) |  |
| No | 15/15 (100.0%) |
| Paclitaxel :  - Duration (months) |  |
| Nobs | 0 |
| Mean (SD) | NA (NA) |
| Median (Q1;Q3) | NA (NA; NA) |
| Min - Max | NA, NA |
| Missing | 33 |
| - Administration frequency |  |
| Nobs | 4 |
| Mean (SD) | 2.00 (1.15) |
| Median (Q1;Q3) | 2.0 (1.0; 3.0) |
| Min - Max | 1.0, 3.0 |
| Missing | 29 |
| - Maintenance dose (cycle) |  |
| Nobs | 2 |
| Mean (SD) | 13.00 (9.90) |
| Median (Q1;Q3) | 13.0 (9.5; 16.5) |
| Min - Max | 6.0, 20.0 |
| Missing | 31 |
| - Maintenance dose (cycle mg/kg or mg) |  |
| Nobs | 1 |
| Mean (SD) | 600.00 (NA) |
| Median (Q1;Q3) | 600.0 (600.0; 600.0) |
| Min - Max | 600.0, 600.0 |
| Missing | 32 |
| - Route of administration, n/N (%) |  |
| Both | 1/4 (25.0%) |
| Intravenous | 3/4 (75.0%) |
| Missing | 29 |
| - Number of cycles completed |  |
| Nobs | 6 |
| Mean (SD) | 5.67 (5.72) |
| Median (Q1;Q3) | 2.0 (2.0; 9.5) |
| Min - Max | 2.0, 14.0 |
| Missing | 27 |
| - Location of administration, n/N (%) |  |
| Hospital | 6/6 (100.0%) |
| Missing | 27 |
| - Start date of treatment available, n/N (%) |  |
| Yes | 0/33 (0.0%) |
| No | 33/33 (100.0%) |
| - End date of treatment available, n/N (%) |  |
| No | 31/31 (100.0%) |
| Tamoxifene :  - Duration (months) |  |
| Nobs | 0 |
| Mean (SD) | NA (NA) |
| Median (Q1;Q3) | NA (NA; NA) |
| Min - Max | NA, NA |
| Missing | 34 |
| - Administration frequency |  |
| Nobs | 4 |
| Mean (SD) | 2.25 (1.50) |
| Median (Q1;Q3) | 2.0 (1.0; 3.2) |
| Min - Max | 1.0, 4.0 |
| Missing | 30 |
| - Maintenance dose (cycle) |  |
| Nobs | 4 |
| Mean (SD) | 1,253.00 (1,439.91) |
| Median (Q1;Q3) | 1,253.0 (6.0; 2,500.0) |
| Min - Max | 6.0, 2,500.0 |
| Missing | 30 |
| - Maintenance dose (cycle mg/kg or mg) |  |
| Nobs | 0 |
| Mean (SD) | NA (NA) |
| Median (Q1;Q3) | NA (NA; NA) |
| Min - Max | NA, NA |
| Missing | 34 |
| - Route of administration, n/N (%) |  |
| Intravenous | 2/3 (66.7%) |
| Subcutaneous | 1/3 (33.3%) |
| Missing | 31 |
| - Number of cycles completed |  |
| Nobs | 2 |
| Mean (SD) | 7.50 (7.78) |
| Median (Q1;Q3) | 7.5 (4.8; 10.2) |
| Min - Max | 2.0, 13.0 |
| Missing | 32 |
| - Location of administration, n/N (%) |  |
| Hospital | 6/6 (100.0%) |
| Missing | 28 |
| - Start date of treatment available, n/N (%) |  |
| Yes | 0/34 (0.0%) |
| No | 34/34 (100.0%) |
| - End date of treatment available, n/N (%) |  |
| No | 31/31 (100.0%) |
| Trastuzumab (Herceptin) :  - Duration (months) |  |
| Nobs | 0 |
| Mean (SD) | NA (NA) |
| Median (Q1;Q3) | NA (NA; NA) |
| Min - Max | NA, NA |
| Missing | 25 |
| - Administration frequency |  |
| Nobs | 2 |
| Mean (SD) | 1.00 (0.00) |
| Median (Q1;Q3) | 1.0 (1.0; 1.0) |
| Min - Max | 1.0, 1.0 |
| Missing | 23 |
| - Maintenance dose (cycle) |  |
| Nobs | 4 |
| Mean (SD) | 6.00 (0.00) |
| Median (Q1;Q3) | 6.0 (6.0; 6.0) |
| Min - Max | 6.0, 6.0 |
| Missing | 21 |
| - Maintenance dose (cycle mg/kg or mg) |  |
| Nobs | 1 |
| Mean (SD) | 20.00 (NA) |
| Median (Q1;Q3) | 20.0 (20.0; 20.0) |
| Min - Max | 20.0, 20.0 |
| Missing | 24 |
| - Route of administration, n/N (%) |  |
| Intravenous | 5/5 (100.0%) |
| Missing | 20 |
| - Number of cycles completed |  |
| Nobs | 4 |
| Mean (SD) | 8.00 (8.37) |
| Median (Q1;Q3) | 6.0 (1.8; 12.2) |
| Min - Max | 1.0, 19.0 |
| Missing | 21 |
| - Location of administration, n/N (%) |  |
| Hospital | 2/2 (100.0%) |
| Missing | 23 |
| - Start date of treatment available, n/N (%) |  |
| Yes | 0/25 (0.0%) |
| No | 25/25 (100.0%) |
| - End date of treatment available, n/N (%) |  |
| No | 24/24 (100.0%) |
| Duration of each adjuvant (months) = (End date of treatment – Start date of treatment + 1) / (365.25/12) | |

## Table 4.3 Time between surgery and adjuvant treatment - Among subjects with at least one adjuvant treatments - Full Analysis Set Population

| Characteristic | All (N = 243) |
| --- | --- |
| Time from surgery to adjuvant treatment initiation of Herceptin (days) |  |
| Nobs | 0 |
| Mean (SD) | NA (NA) |
| Median (Q1;Q3) | NA (NA; NA) |
| Min - Max | Inf, -Inf |
| Missing | 243 |
| Time from surgery to adjuvant treatment initiation of Herceptin (days) = (Date of adjuvant treatment initiation of Herceptin - Surgery date) | |

## Table 4.4 Summary of adjuvant treatments by pCR status - Among subjects with at least one adjuvant treatments - Full Analysis Set Population

| Characteristic | pCR (N = 86) | No pCR (N = 157) |
| --- | --- | --- |
| At least one Adjuvant Treatment* |  |  |
| Docetaxel | 11 (12.8%) | 30 (19.1%) |
| Other | 16 (18.6%) | 22 (14%) |
| Carboplatine | 11 (12.8%) | 24 (15.3%) |
| Anastrozole | 11 (12.8%) | 23 (14.6%) |
| Tamoxifene | 13 (15.1%) | 21 (13.4%) |
| Paclitaxel | 10 (11.6%) | 23 (14.6%) |
| Letrozole | 10 (11.6%) | 18 (11.5%) |
| Navelbine | 7 (8.1%) | 21 (13.4%) |
| Cyclophosphamide | 7 (8.1%) | 20 (12.7%) |
| Epirubicine | 12 (14%) | 14 (8.9%) |
| Exemestane | 8 (9.3%) | 18 (11.5%) |
| Doxorubicine | 9 (10.5%) | 16 (10.2%) |
| Other hormonotherapy 1 | 9 (10.5%) | 16 (10.2%) |
| Trastuzumab (Herceptin) | 6 (7%) | 19 (12.1%) |
| 5-FU | 12 (14%) | 10 (6.4%) |
| Other hormonotherapy 2 | 5 (5.8%) | 12 (7.6%) |

## Table 4.5 Summary of adjuvant treatments by adjuvant treatment by pCR status - Among subjects with at least one adjuvant treatments - Full Analysis Set Population

| Characteristic | pCR (N = 86) | No pCR (N = 157) |
| --- | --- | --- |
| 5-FU :  - Duration (months) |  |  |
| Nobs | 0 | 0 |
| Mean (SD) | NA (NA) | NA (NA) |
| Median (Q1;Q3) | NA (NA; NA) | NA (NA; NA) |
| Min - Max | NA, NA | NA, NA |
| Missing | 12 | 10 |
| - Administration frequency |  |  |
| Nobs | 2 | 1 |
| Mean (SD) | 1.00 (0.00) | 1.00 (NA) |
| Median (Q1;Q3) | 1.0 (1.0; 1.0) | 1.0 (1.0; 1.0) |
| Min - Max | 1.0, 1.0 | 1.0, 1.0 |
| Missing | 10 | 9 |
| - Maintenance dose (cycle) |  |  |
| Nobs | 2 | 1 |
| Mean (SD) | 1,253.00 (1,763.52) | 6.00 (NA) |
| Median (Q1;Q3) | 1,253.0 (629.5; 1,876.5) | 6.0 (6.0; 6.0) |
| Min - Max | 6.0, 2,500.0 | 6.0, 6.0 |
| Missing | 10 | 9 |
| - Maintenance dose (cycle mg/kg or mg) |  |  |
| Nobs | 0 | 1 |
| Mean (SD) | NA (NA) | 20.00 (NA) |
| Median (Q1;Q3) | NA (NA; NA) | 20.0 (20.0; 20.0) |
| Min - Max | NA, NA | 20.0, 20.0 |
| Missing | 12 | 9 |
| - Route of administration, n/N (%) |  |  |
| Intravenous | 3/3 (100.0%) | 1/2 (50.0%) |
| Subcutaneous | 0/3 (0.0%) | 1/2 (50.0%) |
| Missing | 9 | 8 |
| - Number of cycles completed |  |  |
| Nobs | 4 | 2 |
| Mean (SD) | 8.50 (7.68) | 9.50 (10.61) |
| Median (Q1;Q3) | 7.5 (2.0; 14.0) | 9.5 (5.8; 13.2) |
| Min - Max | 2.0, 17.0 | 2.0, 17.0 |
| Missing | 8 | 8 |
| - Location of administration, n/N (%) |  |  |
| Hospital | 2/2 (100.0%) | 1/1 (100.0%) |
| Missing | 10 | 9 |
| Anastrozole :  - Duration (months) |  |  |
| Nobs | 0 | 0 |
| Mean (SD) | NA (NA) | NA (NA) |
| Median (Q1;Q3) | NA (NA; NA) | NA (NA; NA) |
| Min - Max | NA, NA | NA, NA |
| Missing | 11 | 23 |
| - Administration frequency |  |  |
| Nobs | 1 | 2 |
| Mean (SD) | 3.00 (NA) | 3.00 (0.00) |
| Median (Q1;Q3) | 3.0 (3.0; 3.0) | 3.0 (3.0; 3.0) |
| Min - Max | 3.0, 3.0 | 3.0, 3.0 |
| Missing | 10 | 21 |
| - Maintenance dose (cycle) |  |  |
| Nobs | 0 | 3 |
| Mean (SD) | NA (NA) | 1,668.67 (1,439.91) |
| Median (Q1;Q3) | NA (NA; NA) | 2,500.0 (1,253.0; 2,500.0) |
| Min - Max | NA, NA | 6.0, 2,500.0 |
| Missing | 11 | 20 |
| - Maintenance dose (cycle mg/kg or mg) |  |  |
| Nobs | 0 | 0 |
| Mean (SD) | NA (NA) | NA (NA) |
| Median (Q1;Q3) | NA (NA; NA) | NA (NA; NA) |
| Min - Max | NA, NA | NA, NA |
| Missing | 11 | 23 |
| - Route of administration, n/N (%) |  |  |
| Intravenous | 0/0 (NA%) | 2/2 (100.0%) |
| Missing | 11 | 21 |
| - Number of cycles completed |  |  |
| Nobs | 1 | 2 |
| Mean (SD) | 2.00 (NA) | 2.00 (0.00) |
| Median (Q1;Q3) | 2.0 (2.0; 2.0) | 2.0 (2.0; 2.0) |
| Min - Max | 2.0, 2.0 | 2.0, 2.0 |
| Missing | 10 | 21 |
| - Location of administration, n/N (%) |  |  |
| Hospital | 0/0 (NA%) | 1/1 (100.0%) |
| Missing | 11 | 22 |
| Carboplatine :  - Duration (months) |  |  |
| Nobs | 0 | 0 |
| Mean (SD) | NA (NA) | NA (NA) |
| Median (Q1;Q3) | NA (NA; NA) | NA (NA; NA) |
| Min - Max | NA, NA | NA, NA |
| Missing | 11 | 24 |
| - Administration frequency |  |  |
| Nobs | 1 | 2 |
| Mean (SD) | 1.00 (NA) | 2.00 (1.41) |
| Median (Q1;Q3) | 1.0 (1.0; 1.0) | 2.0 (1.5; 2.5) |
| Min - Max | 1.0, 1.0 | 1.0, 3.0 |
| Missing | 10 | 22 |
| - Maintenance dose (cycle) |  |  |
| Nobs | 0 | 3 |
| Mean (SD) | NA (NA) | 837.33 (1,439.91) |
| Median (Q1;Q3) | NA (NA; NA) | 6.0 (6.0; 1,253.0) |
| Min - Max | NA, NA | 6.0, 2,500.0 |
| Missing | 11 | 21 |
| - Maintenance dose (cycle mg/kg or mg) |  |  |
| Nobs | 0 | 3 |
| Mean (SD) | NA (NA) | 120.33 (190.47) |
| Median (Q1;Q3) | NA (NA; NA) | 20.0 (10.5; 180.0) |
| Min - Max | NA, NA | 1.0, 340.0 |
| Missing | 11 | 21 |
| - Route of administration, n/N (%) |  |  |
| Intravenous | 2/2 (100.0%) | 3/3 (100.0%) |
| Missing | 9 | 21 |
| - Number of cycles completed |  |  |
| Nobs | 2 | 3 |
| Mean (SD) | 8.00 (8.49) | 9.33 (8.08) |
| Median (Q1;Q3) | 8.0 (5.0; 11.0) | 8.0 (5.0; 13.0) |
| Min - Max | 2.0, 14.0 | 2.0, 18.0 |
| Missing | 9 | 21 |
| - Location of administration, n/N (%) |  |  |
| Hospital | 1/1 (100.0%) | 2/2 (100.0%) |
| Missing | 10 | 22 |
| Cyclophosphamide :  - Duration (months) |  |  |
| Nobs | 0 | 0 |
| Mean (SD) | NA (NA) | NA (NA) |
| Median (Q1;Q3) | NA (NA; NA) | NA (NA; NA) |
| Min - Max | NA, NA | NA, NA |
| Missing | 7 | 20 |
| - Administration frequency |  |  |
| Nobs | 0 | 3 |
| Mean (SD) | NA (NA) | 3.67 (0.58) |
| Median (Q1;Q3) | NA (NA; NA) | 4.0 (3.5; 4.0) |
| Min - Max | NA, NA | 3.0, 4.0 |
| Missing | 7 | 17 |
| - Maintenance dose (cycle) |  |  |
| Nobs | 0 | 2 |
| Mean (SD) | NA (NA) | 1,253.00 (1,763.52) |
| Median (Q1;Q3) | NA (NA; NA) | 1,253.0 (629.5; 1,876.5) |
| Min - Max | NA, NA | 6.0, 2,500.0 |
| Missing | 7 | 18 |
| - Maintenance dose (cycle mg/kg or mg) |  |  |
| Nobs | 1 | 1 |
| Mean (SD) | 20.00 (NA) | 25.00 (NA) |
| Median (Q1;Q3) | 20.0 (20.0; 20.0) | 25.0 (25.0; 25.0) |
| Min - Max | 20.0, 20.0 | 25.0, 25.0 |
| Missing | 6 | 19 |
| - Route of administration, n/N (%) |  |  |
| Intravenous | 0/0 (NA%) | 2/2 (100.0%) |
| Missing | 7 | 18 |
| - Number of cycles completed |  |  |
| Nobs | 1 | 3 |
| Mean (SD) | 14.00 (NA) | 6.00 (6.93) |
| Median (Q1;Q3) | 14.0 (14.0; 14.0) | 2.0 (2.0; 8.0) |
| Min - Max | 14.0, 14.0 | 2.0, 14.0 |
| Missing | 6 | 17 |
| - Location of administration, n/N (%) |  |  |
| Hospital | 1/1 (100.0%) | 1/1 (100.0%) |
| Missing | 6 | 19 |
| Docetaxel :  - Duration (months) |  |  |
| Nobs | 0 | 0 |
| Mean (SD) | NA (NA) | NA (NA) |
| Median (Q1;Q3) | NA (NA; NA) | NA (NA; NA) |
| Min - Max | NA, NA | NA, NA |
| Missing | 11 | 30 |
| - Administration frequency |  |  |
| Nobs | 0 | 7 |
| Mean (SD) | NA (NA) | 1.71 (1.25) |
| Median (Q1;Q3) | NA (NA; NA) | 1.0 (1.0; 2.0) |
| Min - Max | NA, NA | 1.0, 4.0 |
| Missing | 11 | 23 |
| - Maintenance dose (cycle) |  |  |
| Nobs | 3 | 0 |
| Mean (SD) | 836.00 (1,441.07) | NA (NA) |
| Median (Q1;Q3) | 6.0 (4.0; 1,253.0) | NA (NA; NA) |
| Min - Max | 2.0, 2,500.0 | NA, NA |
| Missing | 8 | 30 |
| - Maintenance dose (cycle mg/kg or mg) |  |  |
| Nobs | 0 | 2 |
| Mean (SD) | NA (NA) | 600.00 (0.00) |
| Median (Q1;Q3) | NA (NA; NA) | 600.0 (600.0; 600.0) |
| Min - Max | NA, NA | 600.0, 600.0 |
| Missing | 11 | 28 |
| - Route of administration, n/N (%) |  |  |
| Intravenous | 2/2 (100.0%) | 2/2 (100.0%) |
| Missing | 9 | 28 |
| - Number of cycles completed |  |  |
| Nobs | 0 | 4 |
| Mean (SD) | NA (NA) | 7.75 (6.65) |
| Median (Q1;Q3) | NA (NA; NA) | 7.5 (2.0; 13.2) |
| Min - Max | NA, NA | 2.0, 14.0 |
| Missing | 11 | 26 |
| - Location of administration, n/N (%) |  |  |
| Hospital | 0/0 (NA%) | 2/2 (100.0%) |
| Missing | 11 | 28 |
| Doxorubicine :  - Duration (months) |  |  |
| Nobs | 0 | 0 |
| Mean (SD) | NA (NA) | NA (NA) |
| Median (Q1;Q3) | NA (NA; NA) | NA (NA; NA) |
| Min - Max | NA, NA | NA, NA |
| Missing | 9 | 16 |
| - Administration frequency |  |  |
| Nobs | 1 | 1 |
| Mean (SD) | 3.00 (NA) | 1.00 (NA) |
| Median (Q1;Q3) | 3.0 (3.0; 3.0) | 1.0 (1.0; 1.0) |
| Min - Max | 3.0, 3.0 | 1.0, 1.0 |
| Missing | 8 | 15 |
| - Maintenance dose (cycle) |  |  |
| Nobs | 4 | 1 |
| Mean (SD) | 629.50 (1,247.00) | 6.00 (NA) |
| Median (Q1;Q3) | 6.0 (6.0; 629.5) | 6.0 (6.0; 6.0) |
| Min - Max | 6.0, 2,500.0 | 6.0, 6.0 |
| Missing | 5 | 15 |
| - Maintenance dose (cycle mg/kg or mg) |  |  |
| Nobs | 0 | 1 |
| Mean (SD) | NA (NA) | 340.00 (NA) |
| Median (Q1;Q3) | NA (NA; NA) | 340.0 (340.0; 340.0) |
| Min - Max | NA, NA | 340.0, 340.0 |
| Missing | 9 | 15 |
| - Route of administration, n/N (%) |  |  |
| Intravenous | 2/2 (100.0%) | 0/0 (NA%) |
| Missing | 7 | 16 |
| - Number of cycles completed |  |  |
| Nobs | 1 | 1 |
| Mean (SD) | 2.00 (NA) | 2.00 (NA) |
| Median (Q1;Q3) | 2.0 (2.0; 2.0) | 2.0 (2.0; 2.0) |
| Min - Max | 2.0, 2.0 | 2.0, 2.0 |
| Missing | 8 | 15 |
| - Location of administration, n/N (%) |  |  |
| Hospital | 1/1 (100.0%) | 2/2 (100.0%) |
| Missing | 8 | 14 |
| Epirubicine :  - Duration (months) |  |  |
| Nobs | 0 | 0 |
| Mean (SD) | NA (NA) | NA (NA) |
| Median (Q1;Q3) | NA (NA; NA) | NA (NA; NA) |
| Min - Max | NA, NA | NA, NA |
| Missing | 12 | 14 |
| - Administration frequency |  |  |
| Nobs | 4 | 1 |
| Mean (SD) | 1.50 (1.00) | 1.00 (NA) |
| Median (Q1;Q3) | 1.0 (1.0; 1.5) | 1.0 (1.0; 1.0) |
| Min - Max | 1.0, 3.0 | 1.0, 1.0 |
| Missing | 8 | 13 |
| - Maintenance dose (cycle) |  |  |
| Nobs | 1 | 1 |
| Mean (SD) | 2,500.00 (NA) | 2,500.00 (NA) |
| Median (Q1;Q3) | 2,500.0 (2,500.0; 2,500.0) | 2,500.0 (2,500.0; 2,500.0) |
| Min - Max | 2,500.0, 2,500.0 | 2,500.0, 2,500.0 |
| Missing | 11 | 13 |
| - Maintenance dose (cycle mg/kg or mg) |  |  |
| Nobs | 0 | 0 |
| Mean (SD) | NA (NA) | NA (NA) |
| Median (Q1;Q3) | NA (NA; NA) | NA (NA; NA) |
| Min - Max | NA, NA | NA, NA |
| Missing | 12 | 14 |
| - Route of administration, n/N (%) |  |  |
| Intravenous | 0/0 (NA%) | 2/2 (100.0%) |
| Missing | 12 | 12 |
| - Number of cycles completed |  |  |
| Nobs | 1 | 2 |
| Mean (SD) | 14.00 (NA) | 2.00 (0.00) |
| Median (Q1;Q3) | 14.0 (14.0; 14.0) | 2.0 (2.0; 2.0) |
| Min - Max | 14.0, 14.0 | 2.0, 2.0 |
| Missing | 11 | 12 |
| - Location of administration, n/N (%) |  |  |
| Hospital | 2/2 (100.0%) | 0/0 (NA%) |
| Missing | 10 | 14 |
| Exemestane :  - Duration (months) |  |  |
| Nobs | 0 | 0 |
| Mean (SD) | NA (NA) | NA (NA) |
| Median (Q1;Q3) | NA (NA; NA) | NA (NA; NA) |
| Min - Max | NA, NA | NA, NA |
| Missing | 8 | 18 |
| - Administration frequency |  |  |
| Nobs | 1 | 4 |
| Mean (SD) | 3.00 (NA) | 1.50 (1.00) |
| Median (Q1;Q3) | 3.0 (3.0; 3.0) | 1.0 (1.0; 1.5) |
| Min - Max | 3.0, 3.0 | 1.0, 3.0 |
| Missing | 7 | 14 |
| - Maintenance dose (cycle) |  |  |
| Nobs | 3 | 1 |
| Mean (SD) | 837.33 (1,439.91) | 6.00 (NA) |
| Median (Q1;Q3) | 6.0 (6.0; 1,253.0) | 6.0 (6.0; 6.0) |
| Min - Max | 6.0, 2,500.0 | 6.0, 6.0 |
| Missing | 5 | 17 |
| - Maintenance dose (cycle mg/kg or mg) |  |  |
| Nobs | 0 | 0 |
| Mean (SD) | NA (NA) | NA (NA) |
| Median (Q1;Q3) | NA (NA; NA) | NA (NA; NA) |
| Min - Max | NA, NA | NA, NA |
| Missing | 8 | 18 |
| - Route of administration, n/N (%) |  |  |
| Intravenous | 0/0 (NA%) | 4/4 (100.0%) |
| Missing | 8 | 14 |
| - Number of cycles completed |  |  |
| Nobs | 1 | 1 |
| Mean (SD) | 2.00 (NA) | 18.00 (NA) |
| Median (Q1;Q3) | 2.0 (2.0; 2.0) | 18.0 (18.0; 18.0) |
| Min - Max | 2.0, 2.0 | 18.0, 18.0 |
| Missing | 7 | 17 |
| - Location of administration, n/N (%) |  |  |
| Hospital | 0/0 (NA%) | 2/2 (100.0%) |
| Missing | 8 | 16 |
| Letrozole :  - Duration (months) |  |  |
| Nobs | 0 | 0 |
| Mean (SD) | NA (NA) | NA (NA) |
| Median (Q1;Q3) | NA (NA; NA) | NA (NA; NA) |
| Min - Max | NA, NA | NA, NA |
| Missing | 10 | 18 |
| - Administration frequency |  |  |
| Nobs | 0 | 4 |
| Mean (SD) | NA (NA) | 2.50 (1.00) |
| Median (Q1;Q3) | NA (NA; NA) | 3.0 (2.5; 3.0) |
| Min - Max | NA, NA | 1.0, 3.0 |
| Missing | 10 | 14 |
| - Maintenance dose (cycle) |  |  |
| Nobs | 2 | 5 |
| Mean (SD) | 2,500.00 (0.00) | 8.80 (6.26) |
| Median (Q1;Q3) | 2,500.0 (2,500.0; 2,500.0) | 6.0 (6.0; 6.0) |
| Min - Max | 2,500.0, 2,500.0 | 6.0, 20.0 |
| Missing | 8 | 13 |
| - Maintenance dose (cycle mg/kg or mg) |  |  |
| Nobs | 1 | 0 |
| Mean (SD) | 600.00 (NA) | NA (NA) |
| Median (Q1;Q3) | 600.0 (600.0; 600.0) | NA (NA; NA) |
| Min - Max | 600.0, 600.0 | NA, NA |
| Missing | 9 | 18 |
| - Route of administration, n/N (%) |  |  |
| Intravenous | 0/0 (NA%) | 2/3 (66.7%) |
| Subcutaneous | 0/0 (NA%) | 1/3 (33.3%) |
| Missing | 10 | 15 |
| - Number of cycles completed |  |  |
| Nobs | 1 | 0 |
| Mean (SD) | 2.00 (NA) | NA (NA) |
| Median (Q1;Q3) | 2.0 (2.0; 2.0) | NA (NA; NA) |
| Min - Max | 2.0, 2.0 | NA, NA |
| Missing | 9 | 18 |
| - Location of administration, n/N (%) |  |  |
| Hospital | 1/1 (100.0%) | 2/2 (100.0%) |
| Missing | 9 | 16 |
| Navelbine :  - Duration (months) |  |  |
| Nobs | 0 | 0 |
| Mean (SD) | NA (NA) | NA (NA) |
| Median (Q1;Q3) | NA (NA; NA) | NA (NA; NA) |
| Min - Max | NA, NA | NA, NA |
| Missing | 7 | 21 |
| - Administration frequency |  |  |
| Nobs | 1 | 4 |
| Mean (SD) | 3.00 (NA) | 3.50 (0.58) |
| Median (Q1;Q3) | 3.0 (3.0; 3.0) | 3.5 (3.0; 4.0) |
| Min - Max | 3.0, 3.0 | 3.0, 4.0 |
| Missing | 6 | 17 |
| - Maintenance dose (cycle) |  |  |
| Nobs | 0 | 2 |
| Mean (SD) | NA (NA) | 2,500.00 (0.00) |
| Median (Q1;Q3) | NA (NA; NA) | 2,500.0 (2,500.0; 2,500.0) |
| Min - Max | NA, NA | 2,500.0, 2,500.0 |
| Missing | 7 | 19 |
| - Maintenance dose (cycle mg/kg or mg) |  |  |
| Nobs | 0 | 0 |
| Mean (SD) | NA (NA) | NA (NA) |
| Median (Q1;Q3) | NA (NA; NA) | NA (NA; NA) |
| Min - Max | NA, NA | NA, NA |
| Missing | 7 | 21 |
| - Route of administration, n/N (%) |  |  |
| Intravenous | 2/2 (100.0%) | 4/4 (100.0%) |
| Missing | 5 | 17 |
| - Number of cycles completed |  |  |
| Nobs | 0 | 5 |
| Mean (SD) | NA (NA) | 3.80 (4.02) |
| Median (Q1;Q3) | NA (NA; NA) | 2.0 (2.0; 2.0) |
| Min - Max | NA, NA | 2.0, 11.0 |
| Missing | 7 | 16 |
| - Location of administration, n/N (%) |  |  |
| Hospital | 1/1 (100.0%) | 2/2 (100.0%) |
| Missing | 6 | 19 |
| Other :  - Duration (months) |  |  |
| Nobs | 0 | 0 |
| Mean (SD) | NA (NA) | NA (NA) |
| Median (Q1;Q3) | NA (NA; NA) | NA (NA; NA) |
| Min - Max | NA, NA | NA, NA |
| Missing | 16 | 22 |
| - Administration frequency |  |  |
| Nobs | 2 | 6 |
| Mean (SD) | 1.00 (0.00) | 1.67 (1.03) |
| Median (Q1;Q3) | 1.0 (1.0; 1.0) | 1.0 (1.0; 2.5) |
| Min - Max | 1.0, 1.0 | 1.0, 3.0 |
| Missing | 14 | 16 |
| - Maintenance dose (cycle) |  |  |
| Nobs | 0 | 1 |
| Mean (SD) | NA (NA) | 6.00 (NA) |
| Median (Q1;Q3) | NA (NA; NA) | 6.0 (6.0; 6.0) |
| Min - Max | NA, NA | 6.0, 6.0 |
| Missing | 16 | 21 |
| - Maintenance dose (cycle mg/kg or mg) |  |  |
| Nobs | 0 | 1 |
| Mean (SD) | NA (NA) | 600.00 (NA) |
| Median (Q1;Q3) | NA (NA; NA) | 600.0 (600.0; 600.0) |
| Min - Max | NA, NA | 600.0, 600.0 |
| Missing | 16 | 21 |
| - Route of administration, n/N (%) |  |  |
| Intravenous | 1/1 (100.0%) | 3/3 (100.0%) |
| Missing | 15 | 19 |
| - Number of cycles completed |  |  |
| Nobs | 1 | 4 |
| Mean (SD) | 12.00 (NA) | 8.00 (6.93) |
| Median (Q1;Q3) | 12.0 (12.0; 12.0) | 8.0 (2.0; 14.0) |
| Min - Max | 12.0, 12.0 | 2.0, 14.0 |
| Missing | 15 | 18 |
| - Location of administration, n/N (%) |  |  |
| Home | 0/1 (0.0%) | 1/3 (33.3%) |
| Hospital | 1/1 (100.0%) | 2/3 (66.7%) |
| Missing | 15 | 19 |
| Other hormonotherapy 1 :  - Duration (months) |  |  |
| Nobs | 0 | 0 |
| Mean (SD) | NA (NA) | NA (NA) |
| Median (Q1;Q3) | NA (NA; NA) | NA (NA; NA) |
| Min - Max | NA, NA | NA, NA |
| Missing | 9 | 16 |
| - Administration frequency |  |  |
| Nobs | 0 | 2 |
| Mean (SD) | NA (NA) | 3.00 (0.00) |
| Median (Q1;Q3) | NA (NA; NA) | 3.0 (3.0; 3.0) |
| Min - Max | NA, NA | 3.0, 3.0 |
| Missing | 9 | 14 |
| - Maintenance dose (cycle) |  |  |
| Nobs | 1 | 1 |
| Mean (SD) | 2,500.00 (NA) | 6.00 (NA) |
| Median (Q1;Q3) | 2,500.0 (2,500.0; 2,500.0) | 6.0 (6.0; 6.0) |
| Min - Max | 2,500.0, 2,500.0 | 6.0, 6.0 |
| Missing | 8 | 15 |
| - Maintenance dose (cycle mg/kg or mg) |  |  |
| Nobs | 0 | 0 |
| Mean (SD) | NA (NA) | NA (NA) |
| Median (Q1;Q3) | NA (NA; NA) | NA (NA; NA) |
| Min - Max | NA, NA | NA, NA |
| Missing | 9 | 16 |
| - Route of administration, n/N (%) |  |  |
| Intravenous | 1/1 (100.0%) | 2/3 (66.7%) |
| Subcutaneous | 0/1 (0.0%) | 1/3 (33.3%) |
| Missing | 8 | 13 |
| - Number of cycles completed |  |  |
| Nobs | 0 | 2 |
| Mean (SD) | NA (NA) | 2.00 (0.00) |
| Median (Q1;Q3) | NA (NA; NA) | 2.0 (2.0; 2.0) |
| Min - Max | NA, NA | 2.0, 2.0 |
| Missing | 9 | 14 |
| - Location of administration, n/N (%) |  |  |
| Hospital | 1/1 (100.0%) | 1/1 (100.0%) |
| Missing | 8 | 15 |
| Other hormonotherapy 2 :  - Duration (months) |  |  |
| Nobs | 0 | 0 |
| Mean (SD) | NA (NA) | NA (NA) |
| Median (Q1;Q3) | NA (NA; NA) | NA (NA; NA) |
| Min - Max | NA, NA | NA, NA |
| Missing | 5 | 12 |
| - Administration frequency |  |  |
| Nobs | 0 | 1 |
| Mean (SD) | NA (NA) | 1.00 (NA) |
| Median (Q1;Q3) | NA (NA; NA) | 1.0 (1.0; 1.0) |
| Min - Max | NA, NA | 1.0, 1.0 |
| Missing | 5 | 11 |
| - Maintenance dose (cycle) |  |  |
| Nobs | 0 | 0 |
| Mean (SD) | NA (NA) | NA (NA) |
| Median (Q1;Q3) | NA (NA; NA) | NA (NA; NA) |
| Min - Max | NA, NA | NA, NA |
| Missing | 5 | 12 |
| - Maintenance dose (cycle mg/kg or mg) |  |  |
| Nobs | 0 | 0 |
| Mean (SD) | NA (NA) | NA (NA) |
| Median (Q1;Q3) | NA (NA; NA) | NA (NA; NA) |
| Min - Max | NA, NA | NA, NA |
| Missing | 5 | 12 |
| - Route of administration, n/N (%) |  |  |
| Intravenous | 3/3 (100.0%) | 1/1 (100.0%) |
| Missing | 2 | 11 |
| - Number of cycles completed |  |  |
| Nobs | 1 | 3 |
| Mean (SD) | 14.00 (NA) | 5.67 (6.35) |
| Median (Q1;Q3) | 14.0 (14.0; 14.0) | 2.0 (2.0; 7.5) |
| Min - Max | 14.0, 14.0 | 2.0, 13.0 |
| Missing | 4 | 9 |
| - Location of administration, n/N (%) |  |  |
| Hospital | 1/1 (100.0%) | 1/1 (100.0%) |
| Missing | 4 | 11 |
| Paclitaxel :  - Duration (months) |  |  |
| Nobs | 0 | 0 |
| Mean (SD) | NA (NA) | NA (NA) |
| Median (Q1;Q3) | NA (NA; NA) | NA (NA; NA) |
| Min - Max | NA, NA | NA, NA |
| Missing | 10 | 23 |
| - Administration frequency |  |  |
| Nobs | 3 | 1 |
| Mean (SD) | 2.33 (1.15) | 1.00 (NA) |
| Median (Q1;Q3) | 3.0 (2.0; 3.0) | 1.0 (1.0; 1.0) |
| Min - Max | 1.0, 3.0 | 1.0, 1.0 |
| Missing | 7 | 22 |
| - Maintenance dose (cycle) |  |  |
| Nobs | 0 | 2 |
| Mean (SD) | NA (NA) | 13.00 (9.90) |
| Median (Q1;Q3) | NA (NA; NA) | 13.0 (9.5; 16.5) |
| Min - Max | NA, NA | 6.0, 20.0 |
| Missing | 10 | 21 |
| - Maintenance dose (cycle mg/kg or mg) |  |  |
| Nobs | 0 | 1 |
| Mean (SD) | NA (NA) | 600.00 (NA) |
| Median (Q1;Q3) | NA (NA; NA) | 600.0 (600.0; 600.0) |
| Min - Max | NA, NA | 600.0, 600.0 |
| Missing | 10 | 22 |
| - Route of administration, n/N (%) |  |  |
| Both | 0/0 (NA%) | 1/4 (25.0%) |
| Intravenous | 0/0 (NA%) | 3/4 (75.0%) |
| Missing | 10 | 19 |
| - Number of cycles completed |  |  |
| Nobs | 3 | 3 |
| Mean (SD) | 6.00 (6.93) | 5.33 (5.77) |
| Median (Q1;Q3) | 2.0 (2.0; 8.0) | 2.0 (2.0; 7.0) |
| Min - Max | 2.0, 14.0 | 2.0, 12.0 |
| Missing | 7 | 20 |
| - Location of administration, n/N (%) |  |  |
| Hospital | 5/5 (100.0%) | 1/1 (100.0%) |
| Missing | 5 | 22 |
| Tamoxifene :  - Duration (months) |  |  |
| Nobs | 0 | 0 |
| Mean (SD) | NA (NA) | NA (NA) |
| Median (Q1;Q3) | NA (NA; NA) | NA (NA; NA) |
| Min - Max | NA, NA | NA, NA |
| Missing | 13 | 21 |
| - Administration frequency |  |  |
| Nobs | 1 | 3 |
| Mean (SD) | 1.00 (NA) | 2.67 (1.53) |
| Median (Q1;Q3) | 1.0 (1.0; 1.0) | 3.0 (2.0; 3.5) |
| Min - Max | 1.0, 1.0 | 1.0, 4.0 |
| Missing | 12 | 18 |
| - Maintenance dose (cycle) |  |  |
| Nobs | 0 | 4 |
| Mean (SD) | NA (NA) | 1,253.00 (1,439.91) |
| Median (Q1;Q3) | NA (NA; NA) | 1,253.0 (6.0; 2,500.0) |
| Min - Max | NA, NA | 6.0, 2,500.0 |
| Missing | 13 | 17 |
| - Maintenance dose (cycle mg/kg or mg) |  |  |
| Nobs | 0 | 0 |
| Mean (SD) | NA (NA) | NA (NA) |
| Median (Q1;Q3) | NA (NA; NA) | NA (NA; NA) |
| Min - Max | NA, NA | NA, NA |
| Missing | 13 | 21 |
| - Route of administration, n/N (%) |  |  |
| Intravenous | 0/0 (NA%) | 2/3 (66.7%) |
| Subcutaneous | 0/0 (NA%) | 1/3 (33.3%) |
| Missing | 13 | 18 |
| - Number of cycles completed |  |  |
| Nobs | 1 | 1 |
| Mean (SD) | 2.00 (NA) | 13.00 (NA) |
| Median (Q1;Q3) | 2.0 (2.0; 2.0) | 13.0 (13.0; 13.0) |
| Min - Max | 2.0, 2.0 | 13.0, 13.0 |
| Missing | 12 | 20 |
| - Location of administration, n/N (%) |  |  |
| Hospital | 3/3 (100.0%) | 3/3 (100.0%) |
| Missing | 10 | 18 |
| Trastuzumab (Herceptin) :  - Duration (months) |  |  |
| Nobs | 0 | 0 |
| Mean (SD) | NA (NA) | NA (NA) |
| Median (Q1;Q3) | NA (NA; NA) | NA (NA; NA) |
| Min - Max | NA, NA | NA, NA |
| Missing | 6 | 19 |
| - Administration frequency |  |  |
| Nobs | 1 | 1 |
| Mean (SD) | 1.00 (NA) | 1.00 (NA) |
| Median (Q1;Q3) | 1.0 (1.0; 1.0) | 1.0 (1.0; 1.0) |
| Min - Max | 1.0, 1.0 | 1.0, 1.0 |
| Missing | 5 | 18 |
| - Maintenance dose (cycle) |  |  |
| Nobs | 0 | 4 |
| Mean (SD) | NA (NA) | 6.00 (0.00) |
| Median (Q1;Q3) | NA (NA; NA) | 6.0 (6.0; 6.0) |
| Min - Max | NA, NA | 6.0, 6.0 |
| Missing | 6 | 15 |
| - Maintenance dose (cycle mg/kg or mg) |  |  |
| Nobs | 0 | 1 |
| Mean (SD) | NA (NA) | 20.00 (NA) |
| Median (Q1;Q3) | NA (NA; NA) | 20.0 (20.0; 20.0) |
| Min - Max | NA, NA | 20.0, 20.0 |
| Missing | 6 | 18 |
| - Route of administration, n/N (%) |  |  |
| Intravenous | 3/3 (100.0%) | 2/2 (100.0%) |
| Missing | 3 | 17 |
| - Number of cycles completed |  |  |
| Nobs | 2 | 2 |
| Mean (SD) | 10.00 (12.73) | 6.00 (5.66) |
| Median (Q1;Q3) | 10.0 (5.5; 14.5) | 6.0 (4.0; 8.0) |
| Min - Max | 1.0, 19.0 | 2.0, 10.0 |
| Missing | 4 | 17 |
| - Location of administration, n/N (%) |  |  |
| Hospital | 0/0 (NA%) | 2/2 (100.0%) |
| Missing | 6 | 17 |
| Duration of each adjuvant (months) = (End date of treatment – Start date of treatment + 1) / (365.25/12) | | |

## Table 4.6 Time between surgery and adjuvant treatment by pCR status - Among subjects with at least one adjuvant treatments - Full Analysis Set Population

| Characteristic | pCR (N = 86) | No pCR (N = 157) |
| --- | --- | --- |
| Time from surgery to adjuvant treatment initiation of Herceptin (days) |  |  |
| Nobs | 0 | 0 |
| Mean (SD) | NA (NA) | NA (NA) |
| Median (Q1;Q3) | NA (NA; NA) | NA (NA; NA) |
| Min - Max | Inf, -Inf | Inf, -Inf |
| Missing | 86 | 157 |
| Time from surgery to adjuvant treatment initiation of Herceptin (days) = (Date of adjuvant treatment initiation of Herceptin - Surgery date) | | |

# 5 Efficacy Analyses

## 5.1 Time to event analyses

### Table 5.1.1 Summary of time from herceptin adjuvant treatment to PFS, overall and by pCR result - Kaplan-Meier estimation - Among subjects with herceptin adjuvant treatment start date available - Full Analysis Set Population

No observation

### Table 5.1.2 Survival probabilities of time from herceptin adjuvant treatment to PFS, overall and by pCR result - Kaplan-Meier estimation - Among subjects with herceptin adjuvant treatment start date available - Full Analysis Set Population

No observation

### Table 5.1.3 Summary of time from herceptin adjuvant treatment to PFS - Kaplan-Meier curve - Among subjects with herceptin adjuvant treatment start date available - Full Analysis Set Population

No observation

### Table 5.1.4 Summary of time from herceptin adjuvant treatment to PFS by pCR result - Kaplan-Meier curve - Among subjects with herceptin adjuvant treatment start date available - Full Analysis Set Population

No observation

# 6 Exploratory Analyses

## 6.1 Predictive factors for PFS

### Table 6.1.1 PFS - Univariate Cox proportional hazard analysis - Among subjects with herceptin adjuvant treatment start date available - Full Analysis Set Population

No observation

### Table 6.1.2 PFS - Multivariate Cox proportional hazard analysis - Among subjects with herceptin adjuvant treatment start date available - Full Analysis Set Population

No observation

## 6.2 Predictive factors for pCR result

### Table 6.2.1 pCR result - Univariate analysis - Full Analysis Set Population

|  | Descriptive statistics | | OR and 95% CI | | |
| --- | --- | --- | --- | --- | --- |
| Characteristic | pCR, N = 108 | No pCR, N = 207 | OR^1^ | 95% CI^1^ | p-value |
| Age at adjuvant treatment initiation of Herceptin (years) |  |  |  |  |  |
| N | 0 | 0 |  |  |  |
| Mean (SD) | NA (NA) | NA (NA) |  |  |  |
| Median (25%; 75%) | NA (NA; NA) | NA (NA; NA) |  |  |  |
| Range | Inf, -Inf | Inf, -Inf |  |  |  |
| Missing | 108 | 207 |  |  |  |
| Age group (years) | 0/0 (NA%) | 0/0 (NA%) |  |  |  |
| Missing | 108 | 207 |  |  |  |
| BMI (kg/m2) |  |  |  |  | 0.592 |
| <25 | 37/98 (37.8%) | 61/98 (62.2%) | — | — |  |
| [25 - 30[ | 39/114 (34.2%) | 75/114 (65.8%) | 0.86 | 0.49, 1.51 |  |
| >=30 | 32/103 (31.1%) | 71/103 (68.9%) | 0.74 | 0.41, 1.33 |  |
| Missing | 0 | 0 |  |  |  |
| T classification |  |  |  |  | 0.335 |
| T0-3 | 84/239 (35.1%) | 155/239 (64.9%) | — | — |  |
| T>3 | 19/66 (28.8%) | 47/66 (71.2%) | 0.75 | 0.40, 1.34 |  |
| Missing | 5 | 5 |  |  |  |
| N classification |  |  |  |  | 0.890 |
| N0 | 37/108 (34.3%) | 71/108 (65.7%) | — | — |  |
| N1 | 46/131 (35.1%) | 85/131 (64.9%) | 1.04 | 0.61, 1.78 |  |
| N2&N3 | 9/27 (33.3%) | 18/27 (66.7%) | 0.96 | 0.38, 2.30 |  |
| Missing | 16 | 33 |  |  |  |
| SBR Grade |  |  |  |  | 0.574 |
| SBR I & II | 47/142 (33.1%) | 95/142 (66.9%) | — | — |  |
| SBR III | 54/149 (36.2%) | 95/149 (63.8%) | 1.15 | 0.71, 1.87 |  |
| Missing | 7 | 17 |  |  |  |
| Presence of vascular emboli |  |  |  |  | 0.341 |
| Yes | 13/29 (44.8%) | 16/29 (55.2%) | — | — |  |
| No | 63/177 (35.6%) | 114/177 (64.4%) | 0.68 | 0.31, 1.53 |  |
| Missing | 32 | 77 |  |  |  |
| Hormonal receptors status |  |  |  |  | 0.559 |
| ER and/or PR + | 86/241 (35.7%) | 155/241 (64.3%) | — | — |  |
| ER and PR - | 19/60 (31.7%) | 41/60 (68.3%) | 0.84 | 0.45, 1.51 |  |
| Missing | 3 | 11 |  |  |  |
| Univariate analysis has been done using a logistic model. P-value is based on a global wald test from logistic model | | | | | |
| ^1^OR = Odds Ratio, CI = Confidence Interval | | | | | |

### Table 6.2.2 pCR result - Multivariate analysis - Full Analysis Set Population

No covariate with p-value<0.15

## 6.3 Predictive factors for PFS and pCR result

### Table 6.3.1 Correlation matrix - Full Analysis Set Population

| Variables | Age (years) | Age group (years) | BMI (kg/m2) | T classification | N classification | SBR Grade | Presence of vascular emboli | Hormonal receptors status | pCR results |
| --- | --- | --- | --- | --- | --- | --- | --- | --- | --- |
| Age (years) | ND |  |  |  |  |  |  |  |  |
| Age group (years) | ND | ND |  |  |  |  |  |  |  |
| BMI (kg/m2) |  |  | ND |  |  |  |  |  |  |
| T classification |  |  | 0.4219 | ND |  |  |  |  |  |
| N classification |  |  | 0.8293 | 0.839 | ND |  |  |  |  |
| SBR Grade |  |  | 0.8227 | 0.2315 | 0.6546 | ND |  |  |  |
| Presence of vascular emboli |  |  | 0.2573 | 0.9724 | 0.8862 | 0.0269 | ND |  |  |
| Hormonal receptors status |  |  | 0.2474 | 1 | 0.5987 | 0.7158 | 0.4005 | ND |  |
| pCR results |  |  | 0.6074 | 0.4123 | 0.9802 | 0.6601 | 0.4546 | 0.665 | ND |
| * pCR results = pCR if ypT0/Tis ypN0 is ticked Yes OR, Grade 1 or Grade 2 are ticked for Classification Chevallier OR, TA and NA are ticked for Classification Sataloff OR, RCB0 is ticked for Classification RCB | | | | | | | | | |
| ND: Not Done | | | | | | | | | |
| Between quantitative and qualitative variables: Anova have been used: the p-value displayed is the p-value of the Type 3 test of fixed effects. P-value is displayed in the above table. | | | | | | | | | |
| Between qualitative variables: Chi² test has been used when all expected counts are >= 5. Otherwise, the Fisher exact test has been used. P-value is displayed in the above table | | | | | | | | | |

### Figure 6.3.2 Correlation coefficient matrix - Full Analysis Set Population


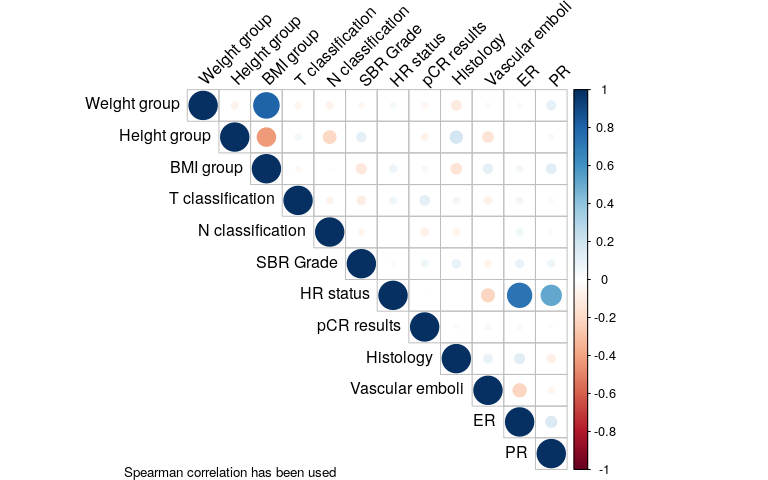

Supplement: S1 File — (ZIP) [file pdig.0000735.s001.zip › Suppl materials parm_statistical_report.docx]
