# Supplementary figures and images for: Comparison of anonymization techniques regarding statistical reproducibility
Source: PLOS Digit Health. 2025 Feb 3;4(2):e0000735. doi: 10.1371/journal.pdig.0000735 (PMC11790161; doi:10.1371/journal.pdig.0000735)

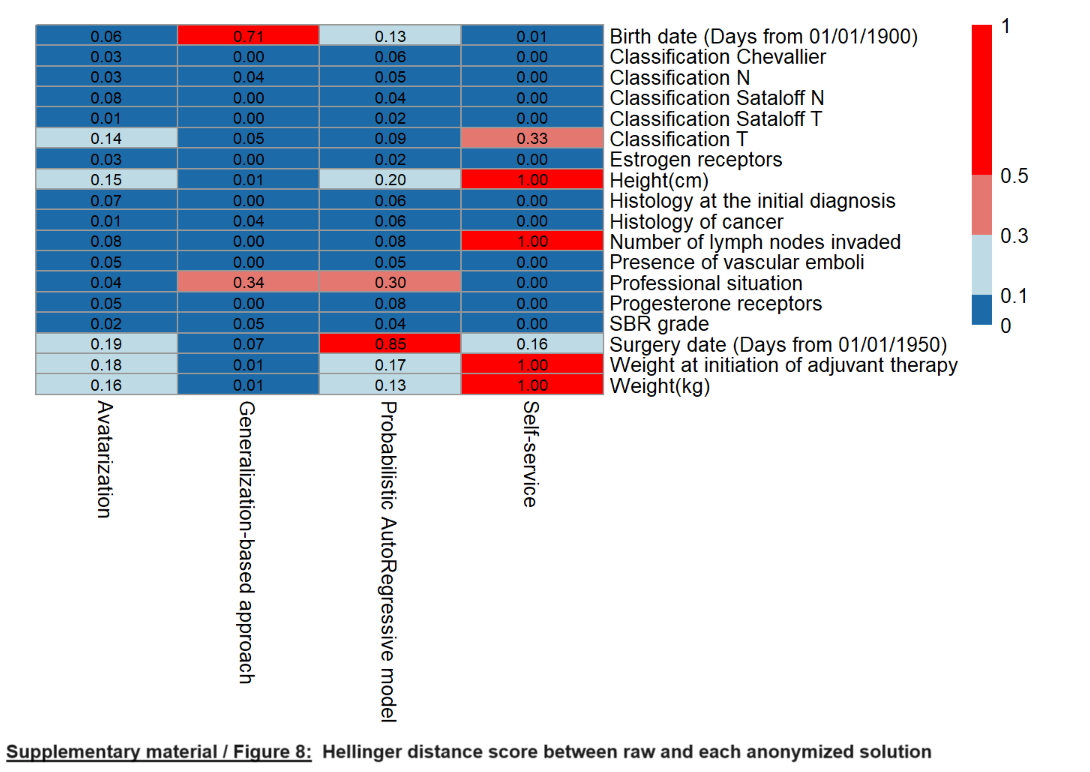

Supplement: S1 File — (ZIP) [file pdig.0000735.s001.zip › Figure 8 Hellinger distances Suppl material.tif]
